# Prediction of Metabolite Concentrations, Rate Constants and Post-Translational Regulation Using Maximum Entropy-Based Simulations with Application to Central Metabolism of *Neurospora crassa*

**DOI:** 10.3390/pr6060063

**Published:** 2018-05-28

**Authors:** William R. Cannon, Jeremy D. Zucker, Douglas J. Baxter, Neeraj Kumar, Scott E. Baker, Jennifer M. Hurley, Jay C. Dunlap

**Affiliations:** 1 Biological Sciences Division, Pacific Northwest National Laboratory, Richland, WA 99352, USA; 2 Research Computing Group, Pacific Northwest National Laboratory, Richland, WA 99352, USA; 3 Environmental Molecular Sciences Laboratory, Pacific Northwest National Laboratory, Richland, WA 99352, USA; 4 Department of Biological Sciences, Rensselaer Polytechnic Institute, Troy, NY 12180, USA; 5 Department of Molecular and Systems Biology, Geisel School of Medicine at Dartmouth, Hanover, NH 03755, USA

**Keywords:** maximum entropy production, mass action kinetics, statistical thermodynamics, metabolism

## Abstract

We report the application of a recently proposed approach for modeling biological systems using a maximum entropy production rate principle in lieu of having in vivo rate constants. The method is applied in four steps: (1) a new ordinary differential equation (ODE) based optimization approach based on Marcelin’s 1910 mass action equation is used to obtain the maximum entropy distribution; (2) the predicted metabolite concentrations are compared to those generally expected from experiments using a loss function from which post-translational regulation of enzymes is inferred; (3) the system is re-optimized with the inferred regulation from which rate constants are determined from the metabolite concentrations and reaction fluxes; and finally (4) a full ODE-based, mass action simulation with rate parameters and allosteric regulation is obtained. From the last step, the power characteristics and resistance of each reaction can be determined. The method is applied to the central metabolism of *Neurospora crassa* and the flow of material through the three competing pathways of upper glycolysis, the non-oxidative pentose phosphate pathway, and the oxidative pentose phosphate pathway are evaluated as a function of the NADP/NADPH ratio. It is predicted that regulation of phosphofructokinase (PFK) and flow through the pentose phosphate pathway are essential for preventing an extreme level of fructose 1,6-bisphophate accumulation. Such an extreme level of fructose 1,6-bisphophate would otherwise result in a glassy cytoplasm with limited diffusion, dramatically decreasing the entropy and energy production rate and, consequently, biological competitiveness.

## 1. Introduction

A grand challenge in biology is to predict the time-dependent behavior of a system. While there have been great successes on the atomistic level that have led to the development of multiscale modeling methods that address phenomena from the femtosecond timescale to the microsecond time scale [1-3], prediction of time-dependent behavior from milliseconds up has been hampered by the lack of rate parameters needed to solve the differential equations governing the behavior.

Many rate parameters have been measured for a few model organisms, but even for those organisms the rate parameters are determined in vitro and do not reflect the in vivo environment. A general solution to this challenge was proposed in 1985 by E. T. Jaynes [4]: “to predict the course of a time-dependent macroscopic process, choose that behavior that can happen in the greatest number of ways while agreeing with whatever information you have—macroscopic or microscopic, equilibrium or nonequilibrium.” The approach that Jaynes was advocating was that of maximum path entropy or maximum entropy production. If one applies constraints to the path, then the method is a variational method referred to as maximum caliber [4,5]. Maximum caliber maximizes a path entropy, subject to the imposed constraints. Jaynes, basing his insights on the writings of Gibbs, assured readers that, “in spite of the conceptual simplicity of the approach, its full mathematical expression does prove to be elegant and intricate after all”.

Entropy and maximum entropy are often confusing topics, so it is useful to first define the terms used herein and explicitly state what is meant by maximum entropy. Fundamentally, entropy itself is a measure of probability while maximum entropy is simply a characterization or description of a distribution. An increase in entropy is an increase in probability, but to be clear, one should always explicitly specify the probability distribution being considered.

Entropy production should not be confused with entropy change, although both are associated with changes of state [6]. An entropy change for chemical systems is often based on a uniform probability distribution as in the common notion for configurational entropy. Less common in the chemical literature but more frequent in the physics literature is the expression of entropy change between two states, *J* and *K* for instance, as a function of the system probability densities, Δ*S*(*K, J*) = − log(Pr(*J*)/Pr(*K*)) where Pr(*J*) and Pr(*K*) are probability density functions based on, for instance, the multinomial Boltzmann distribution [6]. In both cases, entropy change is a state function, meaning that the change in entropy due to a change in state does not depend on which process or path was followed from state to state.

Entropy production, on the other hand, is path-dependent. A maximum entropy production path is the thermodynamically optimal or most probable path. The probability density in the case of mass action chemical systems is the multinomial Boltzmann distribution. A change of state due to following a particular path is related to the thermodynamics odds of each respective reaction *i*, $K_{i}Q_{i}^{-1}$, where *K_i_* is the chemical equilibrium constant and *Q_i_* is the reaction quotient. Finding the optimal path for a system is a general problem [7], but is especially important for biology where efficiency and time-to-replication are critical for natural selection. Finally, maximum entropy production and maximum caliber are very similar concepts. A maximum entropy production path is the path that produces a maximal amount of entropy [8,9]. (A minimum entropy production path is a path that dissipates a minimal amount of heat or entropy to the environment. For the purpose of this paper, the two concepts are equivalent and ‘least heat’ can be used as a working definition of optimal.) A maximum caliber method specifically maximizes the entropy of the path subject to constraints on the system [10]. Either method may be either inferential (applied for the purpose of data analysis) or predictive (employed in a simulation). For the intents and purposes of this paper, we shall not make a distinction between the maximum entropy production and maximum caliber, and will use the term maximum entropy production.

A maximum entropy method is a general term that can refer either to methods that employ an entropy change, or to methods that employ entropy production; it can refer to either prediction through simulation or inference from data. Maximum entropy production specifically refers to a thermodynamically optimal path from one state to another and may be used in the context of either simulations, descriptions of processes, or for the purpose of inference [4]. Jaynes used the term MAXENT to emphasize the use of maximum entropy methods for inference. Regardless of whether maximum entropy is used in the context of inference or prediction, or an entropy change or entropy production, a maximum entropy distribution is the most probable distribution according to the probability density function and boundary conditions.

Maximum entropy production, or more precisely, maximum entropy production rate—the rate at which the maximum amount of entropy is produced—has a long history in physical biology. Lotka first wrote about the concept in 1922, explaining that “in the struggle for existence, the advantage must go to those organisms whose energy-capturing devices are most efficient in directing available energy into channels favorable to the preservation of the species” ([11,12]; reviewed in [13]). At face value, Lotka’s statement seems obvious in retrospect, but he went on to advocate that natural selection is based on the physical principles of thermodynamics. Surprisingly, in his report, Lotka states that Boltzmann had been talking about these concepts years earlier. More famously Schrodinger in his 1945 monograph *What is Life?* [14], used the concept of entropy to describe how order, in the form of high-energy compounds in the environment, drives organization within organisms. Prigogine, who received the Nobel prize in 1977 for his work, used the related concept of minimization of entropy production (defined above) to explain the emergence of self-organized systems from non-equilibrium conditions [15]. Recently, Dewar [16] has examined the maximum entropy production concept in detail, noting that when the predictions from maximum entropy approaches fail, it is not the principle that is inadequate but rather the model to which the principle is applied is usually insufficient for the level of prediction needed. While some view this to be a controversial statement, we have observed this to be true in our own work.

Previously, we developed and implemented a maximum entropy production approach to evaluate the dynamics of different versions of the tricarboxylic acid (TCA) cycle found in nature using stochastic kinetics [17] and have more recently generalized the concept to show how simulations can be carried out from knowledge of chemical potentials [18]. In the former study, the rate of the processes were assumed to all occur on the same timescale. In this study, we extend that work to central metabolism including glycolysis and the pentose phosphate pathway using deterministic kinetics. The challenge in both approaches is to find the maximum entropy or most probable distribution, according to a multinomial Boltzmann probability density, consistent with the non-equilibrium boundary conditions. Since rate parameters (and also transition probabilities) have inherent time dependence, a mathematical approach to finding the distribution must be capable of circumventing bottlenecks in phase space that prevent the system from locating the global minima. One solution to this challenge is to widely sample parameter space using ensemble modeling to find the parameters that agree with known behavior or observations [19]. The alternative, as suggested by Jaynes, is to “choose that behavior that can happen in the greatest number of ways”, which is to choose the most thermodynamically probable parameters.

In this report, we show that the most thermodynamically probable concentrations can be predicted using a modified version of Marcelin’s 1910 equation describing mass action dynamics using reaction affinities [20]. The first step is to obtain the maximum entropy distribution by finding a non-equilibrium steady state that is also a thermodynamic stable state, meaning that the net driving forces on all reactions are equal. When only mass action kinetics are considered in the model and other considerations such as diffusion are ignored, we find that the resulting maximum entropy distribution is appropriate only for the mass action model, but not for a more extensive model that would include diffusion. The discrepancy, as Dewar predicted [16], is not due to the application of the maximum entropy rate principle but rather to the incomplete nature of the model. However, comparison of the predicted metabolite concentrations to expected or observed concentrations allows one to infer points of regulation in the pathways using a loss function similar to that used in machine learning. Once regulation is accounted for, reasonable metabolite concentrations are predicted, as are reaction fluxes. From the concentrations and reaction fluxes, rate parameters are inferred, and full-scale mass action differential equations can be solved, resulting in the time-dependent trajectories of the biological pathways. In doing so, we have implemented Jaynes’ proposal of using a simple thermodynamic principle to infer the detailed dynamics of a system without the need to construct the detailed dynamics or parameters from the bottom up. Finally, we evaluate the dynamics of central metabolism of *Neurospora crassa*, a model organism for studying the multi-scale dynamics of circadian rhythms. We find that the pentose phosphate pathway can act in a cyclical manner to produce six NADPH for every glucose consumed instead of the generally expected two NADPH per glucose consumed. Together with recent findings that the oxidative pentose phosphate pathway and upper glycolysis are 180 degrees out of phase in the circadian cycle [21], suggests that under nitrogen-limiting conditions when the pentoses are not used for DNA synthesis, the cyclic action of the pentose phosphate pathway may be used to maximize NADPH production for lipid or carbohydrate production.

## 2. Theory and Methods

### Maximum Entropy Optimization Using the Marcelin Equation.

Consider a reversible chemical reaction with molecular species *i* ∈ {*A, B, C, D*} and unsigned stoichiometric coefficients *v_i,α_* for each molecular species *i* in reaction *α* ∈ {1, −1} with forward reaction *α* = 1 and the reverse reaction *α* = −1,

(shcheme 1) $$v_{A,1}n_{A}+v_{B,1}n_{B}\overset{rxn\;1}{\underset{rxn\;-1}{⇌}}v_{C,1}n_{C}+v_{D,1}n_{D}$$

where *n_i_* is the count or concentration of species *i*. The extent of each of the reactions is given by *ζ*_1_ and *ζ*_−1_, such that when *ζ*_1_ = 0, the system is in the state where neither of the *n_A_* and *n_B_* reactants have turned into products. When *ζ*_1_ = 1, the system is in the state where a stoichiometric amount of the *n_A_* and *n_B_* reactants have turned into products such that there are now *n_C_* + *v*_*C*,1_ and *n_D_* + *v*_*D*,1_ products and *n_A_* − *v*_*A*,1_ and *n_B_* − *v*_*B*,1_ reactants. The net extent of each reversible reaction is *ζ*_1_ − *ζ*_−1_ ≡ *ζ*_1,*net*_ and *ζ*_−1_ − *ζ*_1_ ≡ *ζ*_−1,*net*_. Consequently, $d\zeta_{1,net}∕dt={\dot{\zeta }}_{1,net}$ is the net flux through reaction 1. The mass action rate law for the reaction in Scheme 1 is,

(1) $${\dot{\zeta }}_{1,net}=\frac{d\zeta_{1,net}}{dt}=k_{1}{n_{A}}^{v_{A,1}}{n_{B}}^{v_{B,1}}-k_{-1}{n_{C}}^{v_{C,1}}{n_{D}}^{v_{D},1}.$$

where *k*_1_ and *k*_−1_ are the rate constants of the forward and the reverse reaction, respectively. Using the signed stoichiometric coefficients *γ*_*i*,1_ such that *γ*_*i*,1_ = −|*v*_*i*,1_| for reactants and *γ*_*i*,1_ =|*v*_*i*,1_| for products, the time-dependence of any molecular species *i* is,

(2) $$\gamma_{i,1}\frac{dn_{i}}{dt}=k_{1}{n_{A}}^{v_{A,1}}{n_{B}}^{v_{B,1}}-k_{-1}{n_{C}}^{v_{C,1}}{n_{D}}^{v_{D,1}}.$$

Although kinetics and thermodynamics are alternate formulations of the law of mass action, Equation (2) is a purely kinetic description in that it does not contain any thermodynamic functions. Thermodynamics can be introduced into Equation (2) by simply factoring out the opposing rate from each term,

$$\gamma_{i,1}\frac{dn_{i}}{dt}\;\;=k_{1}{n_{A}}^{v_{A,1}}{n_{B}}^{v_{B,1}}\left(\frac{k_{-1}{n_{C}}^{v_{C,1}}{n_{D}}^{v_{D,1}}}{k_{-1}{n_{C}}^{v_{C,1}}{n_{D}}^{v_{D,1}}}\right)-k_{-1}{n_{C}}^{v_{C,1}}{n_{D}}^{v_{D,1}}\left(\frac{k_{1}{n_{A}}^{v_{A,1}}{n_{B}}^{v_{B,1}}}{k_{1}{n_{A}}^{v_{A,1}}{n_{B}}^{v_{B,1}}}\right)\;\;=k_{-1}{n_{C}}^{v_{C,1}}{n_{D}}^{v_{D,1}}\left(\frac{k_{1}{n_{A}}^{v_{A,1}}{n_{B}}^{v_{B,1}}}{k_{-1}{n_{C}}^{v_{C,1}}{n_{D}}^{v_{D,1}}}\right)-k_{1}{n_{A}}^{v_{A,1}}{n_{B}}^{v_{B,1}}\left(\frac{k_{-1}{n_{C}}^{v_{C,1}}{n_{D}}^{v_{D,1}}}{k_{1}{n_{A}}^{v_{A,1}}{n_{B}}^{v_{B,1}}}\right)\;\;=k_{-1}{n_{C}}^{v_{C,1}}{n_{D}}^{v_{D,1}}\left(K_{1}\frac{{n_{A}}^{v_{A,1}}{n_{B}}^{v_{B,1}}}{{n_{C}}^{v_{C,1}}{n_{D}}^{v_{D,1}}}\right)-k_{1}{n_{A}}^{v_{A,1}}{n_{B}}^{v_{B,1}}\left(K_{-1}\frac{{n_{C}}^{v_{C,1}}{n_{D}}^{v_{D,1}}}{{n_{A}}^{v_{A,1}}{n_{B}}^{v_{B,1}}}\right),$$

or,

(3) $$={\dot{\zeta }}_{-1}(t,n_{i})e^{A_{1}(n_{i})∕RT}-{\dot{\zeta }}_{1}(t,n_{i})e^{A_{-1}(n_{i})∕RT}$$

Equation (3) is the equation for the Marcelin–de Donder representation of mass action kinetics [22,23]. The first term describes the time-dependent thermodynamic forces acting on reaction 1 and likewise the second term describes the time-dependent thermodynamic forces acting on the opposing reaction −1. The first term is a product of a purely thermodynamic component, *e*^*A*_1_(*n_i_*)/*RT*^, which is the exponential of the thermodynamic driving force *A*_1_(*n_i_*) on reaction 1, and a purely kinetic component, ${\dot{\zeta }}_{-1}(t,n_{i})$ which is the time derivative of the extent of the opposing reaction −1, ${\dot{\zeta }}_{-1}(t,n_{i})=d\zeta_{-1}∕dt$. The thermodynamic factor *e*^*A*_1_(*n_i_*)/*RT*^ is the odds ratio of the forward reaction to the reverse reaction. The odds are reciprocally related such that *e*^*A*_1_/*RT*^ = *e*^−*A*_−1_/*RT*^. The odds of reaction 1 then change on a time scale determined by ${\dot{\zeta }}_{-1}(t,n_{i})$. That is, the thermodynamic driving force on the forward reaction has a relaxation time that is the time for the reverse reaction to occur. Likewise, the second term describes the odds ratio and time dependence of the odds of the reverse (conjugate) reaction, reaction −1. According to this formulation, positive non-equilibrium forces (*A*_1_(*n_i_*) > 0) will be associated with slower changes in the odds of the forward reaction than the odds of the opposing reaction since the respective relaxation times of the odds are inversely related through the relation,

(4) $${\dot{\zeta }}_{-1}(t,n_{i})e^{A_{1}(n_{i})∕RT}={\dot{\zeta }}_{1}(t,n_{i}).$$

Equation (4) has the same mathematical form of a fluctuation theorem [24] but is an exact relationship,

$$e^{A_{1}(n_{i})∕RT}=\frac{{\dot{\zeta }}_{1}(t,n_{i})}{{\dot{\zeta }}_{-1}(t,n_{i})}.$$

Generalizing to a large system consisting of *Z* reactions, the time-dependence of chemical species *i* is given by,

(5) $$\frac{dn_{i}}{dt}=\sum_{\alpha }^{Z}\frac{1}{\gamma_{i,\alpha }}\left({\dot{\zeta }}_{-\alpha }(t,n_{i})e^{A_{\alpha }(n_{i})∕RT}-{\dot{\zeta }}_{\alpha }(t,n_{i})e^{-A_{\alpha }(n_{i})∕RT}\right).$$

A convenient thermodynamic optimization procedure can be obtained using the Marcelin formulation of Equation (5). The Marcelin Equation [20] is obtained by setting each of the functions ${\dot{\zeta }}_{\alpha }(t,n_{i})\neq 0\;\text{to}\;{\dot{\zeta }}_{\alpha }(t,n_{i})=c_{\alpha }$ where *c_α_* is a constant,

$$\frac{dn_{i}}{dt}=\sum_{\alpha }^{Z}c_{\alpha }\frac{1}{\gamma_{i,\alpha }}\left(e^{A_{\alpha }(n_{i})∕RT}-e^{-A_{\alpha }(n_{i})∕RT}\right).$$

That is, the Marcelin Equation sets each of the relaxation rates of the forward and reverse forces to the same rate. Assuming the same relaxation rate for each respective force for all reactions *α* such that *c*_*α*_ = *c* removes any kinetic bottlenecks in phase space of the system such that the relative dynamics are governed only by the thermodynamics,

(6) $$\frac{1}{c}\frac{dn_{i}}{dt}=\sum_{\alpha }^{Z}\frac{1}{\gamma_{i,\alpha }}\left(e^{A_{\alpha }(n_{i})∕RT}-e^{-A_{\alpha }(n_{i})∕RT}\right).$$

A simulation using Equation (6) will converge to a thermodynamically-optimal steady state, which will be the lowest free energy state given the boundary conditions. The reason for this is that the free energy of chemical systems is the negative log of the multinomial (discrete particle counts) or Dirichlet (continuous particle counts) distribution plus a constant [6]. The multinomial and Dirichlet distributions are members of the exponential family of distributions, which are log-concave when counts are always greater than or equal to zero. Since the free energy is the negative of the log of the distribution, the free energy in this case is a convex surface as a function of the counts. For this thermodynamically-optimal steady state, the net flux through a reaction *α* is given by,

(7) $${\dot{\zeta }}_{\alpha ,net}=c\left(e^{A_{\alpha }(n_{i})∕RT}-e^{-A_{\alpha }(n_{i})∕RT}\right).$$

Accordingly, at the thermodynamically-optimal steady state, the net flux through a reaction is proportional to the net thermodynamic odds. The thermodynamically-optimal steady state is one of many possible kinetic steady states, but its dynamics are such that the state is both kinetically and thermodynamically stable. That is, given a stoichiometric matrix **S** of *M* metabolites × *Z* reactions and a vector of the net reaction fluxes $\dot{\xi }$ of length *Z*, the usual steady state condition applies,

$$S\cdot \dot{\xi }=0.$$

The thermodynamic odds of a reaction in Equation (7) are such that $e^{A_{\alpha }∕RT}=K_{\alpha }Q_{\alpha }^{-1}$ where *K_α_* is the equilibrium constant and *Q_α_* is the reaction quotient for reaction *α*. Given *Z* by *Z* diagonal matrices of forward and reverse equilibrium constants, **K**^+^ and **K**^−^, and diagonal matrices of the respective forward and reverse reaction quotients, **Q**^+^ and **Q**^−^, the thermodynamically-optimal steady state is the product of the stoichiometric matrix and the net thermodynamic odds of each reaction,

$$S\cdot ((K^{+}Q^{-}-K^{-}Q^{+})\cdot 1)=0.$$

That is, the system is thermodynamically stable as well as kinetically stable. Furthermore, since the parameter *c* in Equation (7) is arbitrary, the maximum entropy production distribution and the maximum entropy production rate distribution are equivalent. Equation (7) is an important result as relative rate constants can be determined for each reaction *α*, as will be shown below.

### Agreement with Experimental Measurements.

It is possible that the thermodynamically-optimal steady state may not have metabolite levels at physiologically realistic values. For example, inference of a steady state model of central metabolism will predict concentrations of fructose 1,6-bisphosphate in excess of 1 M (see below). If the cell were to produce these large concentrations, the cytoplasm would become glassy and practically no diffusion would occur. Hence the entropy production rate would actually approach zero. The issue is that the model assumes that diffusion will remain sufficient regardless of solute concentrations. This is easily remedied without including diffusion in the model by comparing predicted concentrations or counts of metabolite *i*, ${\tilde{n}}_{i}$, to experimentally observed values *n_i_* and reducing the difference between predicted and observed values by optimizing a loss function for each reaction *α*. As a loss function, we have chosen the log ratio of the observed values to the predicted values of the *M*(*α*) reaction products of reaction *α*,

(8) $$L_{\alpha }=\log\prod_{i(\alpha )}^{M(\alpha )}\frac{{\tilde{n}}_{i(\alpha )}}{n_{i(\alpha )}}.$$

When the observed and predicted values agree, *L_α_* = 0, and when the predicted values are greater or less than the observed values *L_α_* > 0 or *L_α_* < 0, respectively. Unfortunately, experimental measurements of metabolite concentrations are hard to obtain; even when available, metabolomics data sets are sparse. Part of the reason is that the chemical properties of small molecules vary widely, so no single experimental design can discriminate and measure each molecular species. Sparsity is also due to the fact that biologically relevant metabolite concentrations may span several orders of magnitude, which is often greater than typical instrument dynamic ranges. Instead, one can simply use rule-of-thumb estimates for metabolite concentrations in place of experimentally observed values. Based on mass spectrometry estimates of absolute concentrations of metabolites [25], a reasonable rule-of-thumb for metabolites is that most metabolites will not exceed millimolar levels. A value of *L_α_* > 0 may indicate that the system needs to be modulated or regulated such that the metabolite concentrations stay within a physiological range. The desired regulation can be implemented using any appropriate function, such as a Hill equation, a logistic function or a hyperbolic function. Parameters for the regulation functions are then estimated and the simulations and analysis are repeated, adjusting parameters each time, until a physiological level of metabolite concentrations is achieved. This resulting steady state is thermodynamically optimal conditioned on the required regulation.

### Rate Constants.

From this steady state with reasonable metabolite concentrations and activity *λ_α_* of reaction *α*, the rate constants are inferred for each reaction as follows. The net flux ${\dot{\zeta }}_{\alpha ,net}$ is,

$${\dot{\zeta }}_{\alpha ,net}\;\;=\lambda_{\alpha }\cdot k_{\alpha }\prod_{i}^{\text{reactants}\;\alpha }n_{i}-\lambda_{\alpha }\cdot k_{-\alpha }\prod_{i}^{\text{reactants}\;-\alpha }n_{i}\;\;=\lambda_{\alpha }\cdot k_{\alpha }\prod_{i}^{\text{reactants}\;\alpha }n_{i}\left(1-\frac{\lambda_{\alpha }\cdot k_{-\alpha }\prod_{i}^{\text{reactants}\;-\alpha }n_{i}}{\lambda_{\alpha }\cdot k_{\alpha }\prod_{i}^{\text{reactants}\;\alpha }n_{i}}\right)\;\;=\lambda_{\alpha }\cdot k_{\alpha }\prod_{i}^{\text{reactants}\;\alpha }n_{i}(1-K_{-\alpha }Q_{-\alpha }^{-1}),$$

where again *λ_α_* is the activity of the enzyme catalyzing reaction *α* as a function of the regulation. For example, solving for the rate constants of reaction *α* = 1 from Scheme 1 gives,

(9) $$\;k_{1}=c\frac{{\dot{\zeta }}_{1,net}}{\lambda_{1}\cdot n_{A}n_{B}(1-K_{-1}Q_{-1}^{-1})}.\;k_{-1}=c\frac{K_{1}}{k_{1}},$$

since *λ*_1_ = 1 (no regulation) in Scheme 1. The usual mass action ODEs using rate constants and regulation are then solved during a simulation. The kinetically accessible energy surface is not necessarily convex because of the introduction of the rate constants—each reaction now has its own time dependence.

Finally, for the readers that are interested in such details, the inference of rate parameters in this manner meets the criteria that Jaynes laid out for MAXENT: *when we make inferences based on incomplete information, we should draw them from that probability distribution that has the maximum entropy permitted by the information we do have* [26]. Here, we have used the maximum entropy distribution from the maximum entropy production simulation to infer parameters that we otherwise know nothing about. However, if one has accurate metabolite measurements or fluxes of the metabolite population that is free in its biological solution (e.g., cytoplasm), then one can also use this information to infer experimentally-based in vivo rate constants as well [18].

### Power and Conductance.

For complex systems such as biological systems, the power is the change in free energy with respect to time,

$$P=-\frac{dG}{dt}.$$

For a reaction, the rate dependence is due to the net change in the extent of a reaction *ζ_α,net_* with time such that the rate ${\dot{\zeta }}_{\alpha ,net}=d\zeta_{\alpha ,net}∕dt$ is the usual mass action rate as exemplified by Equation (1). The power generated by a reaction can then be expressed in terms of the reaction affinity *A_α_* and the extent of the reaction *ζ_α_*,

$$P_{\alpha }\;\;=-\frac{dG}{d\zeta_{\alpha }}\frac{d\zeta_{\alpha }}{dt}\;\;=A_{\alpha }{\dot{\zeta }}_{\alpha }.$$

This relationship is useful for comparing different chemical processes, as will be seen below. Likewise, the resistance *R_α_* and conductance *C_α_* of reaction *α* can be calculated at steady state as,

$$R_{\alpha }=\frac{\Delta G_{\alpha }}{{\dot{\zeta }}_{\alpha ,net}},\;C_{\alpha }=R_{\alpha }^{-1}.$$

where Δ*G_α_* is the free energy change across the reaction, which is equal in value to the reaction affinity *A_α_* for a system at steady state with a large number of particles. These latter equations should not be taken to imply that there is a linear relationship between flux and a change in free energy (or flux and resistance). A reaction at steady state has high resistance if the change in free energy is large for the steady state flux relative to other reactions in the pathway. An example of this will be discussed in the results section, in which a regulated reaction has the same flux as other reactions at steady state, but the change in free energy for the reaction is large because the regulation decreases the activity of the corresponding enzyme.

### Implementation, Code, Metabolic Model and Parameters.

The equations described above for both the optimization and simulation were implemented in the language C in a software program called Boltzmann, which is available as open source code under a Berkeley Software Distribution (BSD) style license [27]. The ODE solver was an implementation of the MATLAB® ode23tb solver, a trapezoidal rule and the backward differentiation solver [28].

Chemical potentials were obtained from component contribution methods [29,30] and adjusted within Boltzmann to the dielectric response *ϵ*, ionic strength *I*, and pH of the cell cytoplasm, assumed to be *ϵ* = 0.78, *I* = 0.25, and pH = 7.0 [31].

The compartmentalized *Neurospora crassa* metabolic model originally from Dreyfuss et al. [32] was updated with new information and used for the optimizations and simulations. The updated genome-scale model is publicly available [33]. The model used in this work is a subnetwork of the genome-scale model that includes only central metabolism consisting of upper and lower glycolysis, the TCA cycle, and the pentose phosphate cycle and is available in the supplemental notebooks. In this reduced model, there are 20 variables (metabolite concentrations) and 20 equations (reaction equations).

In order to maintain a non-equilibrium state, boundary concentrations for the initial reactant glucose 6-phosphate and final product CO_2_ are set to non-equilibrium values of 2 mM and 0.1 mM, respectively. Likewise, the cofactors CoA, ATP, ADP, orthophosphate, NAD, NADH, NADP and NADPH are also fixed boundary species; these concentrations were taken from a mass spectrometry analysis of absolute metabolite concentrations from Bennett et al. [25] The redox pair employed to shuttle electrons into the mitochondrial respiratory chain were taken to have equal chemical potentials, as done in previous modeling of the TCA cycle [17].

Analysis of simulation data is included in supplementary information/computational notebooks using Python.

## 3. Results

A metabolic model of *Neurospora crassa* [32] was used along with chemical potentials to maximize the entropy production rate of central metabolism. The dynamics of the each species are governed by the thermodynamic forces acting on each reaction (Equation (6)) and the net flux through each reaction is determined by the thermodynamic odds of the reaction at steady state (Equation (7)).

**Figure 1.**
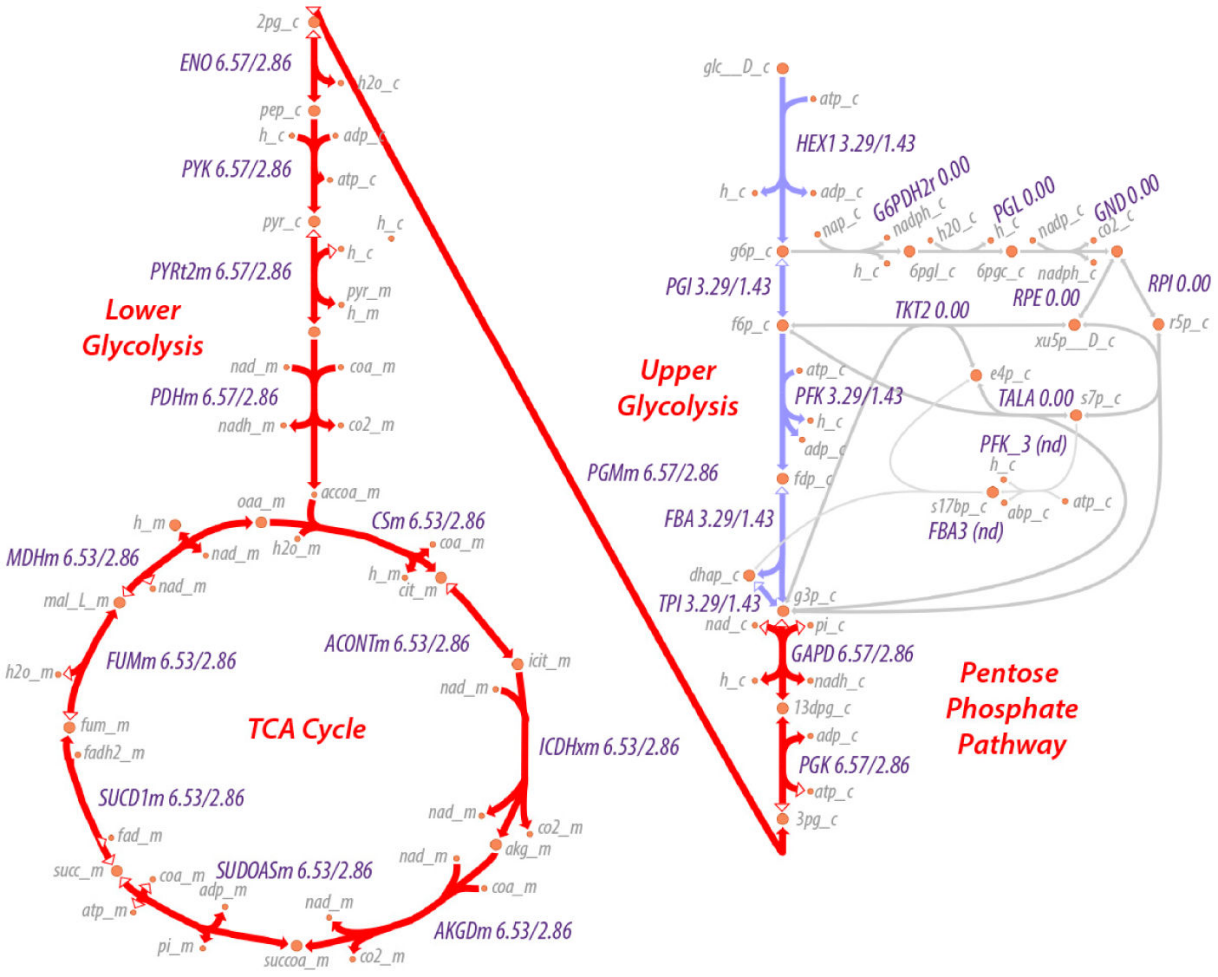
Map of net odds (Equation (7)) for reactions of glycolysis and the tricarboxylic acid (TCA) cycle. Values next to each reaction name indicate the net flux through the reaction, and two flux values are provided for each reaction. The first value (left) is the flux after maximum entropy optimization. The second value (right) is the flux after the same optimization but including regulation at PFK (by ATP) and PDHm (by acetyl-CoA). Reaction and metabolite abbreviations are derived from the BiGG database [34]; full common names are provided as supplementary Tables S1 and S2. The metabolic pathway visualizations here and in Figure 5 were created with Escher [35].

A map of the flux through the system is shown in Figure 1. As can be seen, the maximum entropy production rate optimization predicts that the fluxes ${\dot{\zeta }}_{\alpha ,net}$ in lower glycolysis and the TCA cycle $\left({\dot{\zeta }}_{\alpha ,net}\approx 6.53\right)$ are twice that of upper glycolysis $\left({\dot{\zeta }}_{\alpha ,net}\approx 3.29\right)$, as would be expected since an intermediate in upper glycolysis corresponds to two intermediates in lower glycolysis and the TCA cycle due to the splitting of fructose 1,6-bisphosphate by fructose 1,6-bisphosphate aldolase to effectively two molecules of glyceraldehyde-3-phosphate. The flux values are relative values but can be calibrated to absolute values by knowledge of just one absolute reaction flux.

The maximum entropy production optimization results in metabolite levels tending towards their most probable (Boltzmann) distribution such that,

$$\frac{n_{i}}{N_{T}}\propto \frac{e^{-\mu_{i}{}^{0}(\epsilon ,I,pH)∕RT}}{\sum_{j}^{M}e^{-\mu_{j}{}^{0}(\epsilon ,I,pH)∕RT}},$$

where $\mu_{i}^{0}(\epsilon ,I,pH)$ is the standard chemical potential for solute *i* in an aqueous solution with dielectric constant *ϵ*, ionic strength *I*, and constant pH. The values of *ϵ*, *I*, *pH* are chosen to match those in the cell environment, which in this case are assumed to be *ϵ* = 0.15, *I* = 78 and *pH* = 7.0. The non-equilibrium boundary conditions for the initial reactant glucose and final product CO_2_, and with those for the cofactors CoA, ATP, ADP, orthophosphate, NAD, NADH, NADP and NADPH prevent the intermediates of glycolysis and the TCA cycle from reaching equilibrium.

**Figure 2.**
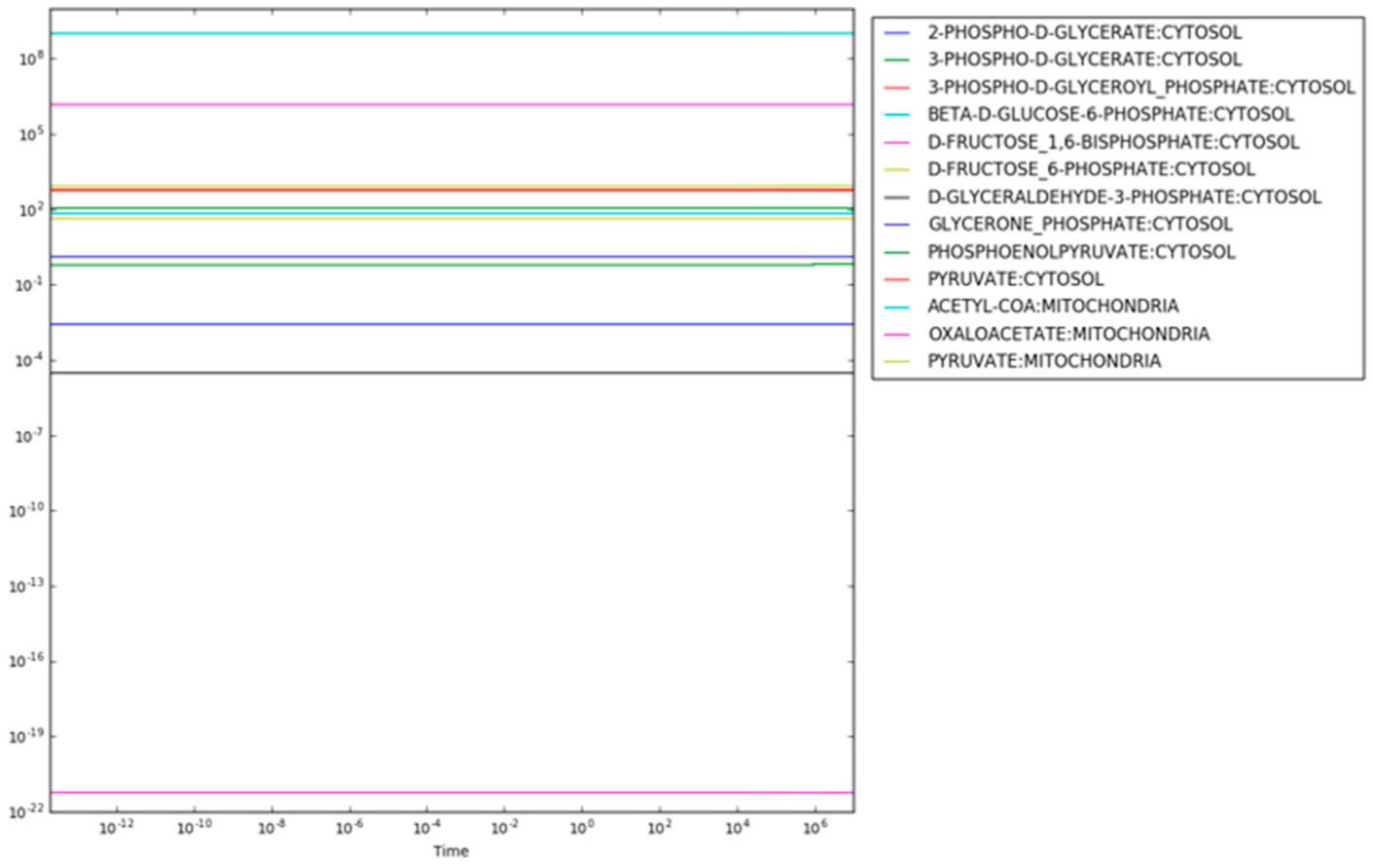
Concentrations as a function of time in maximum entropy optimization without regulation.

The resulting maximum entropy production rate concentrations are shown in Figure 2. There are two important aspects of the results shown in Figure 2. First, the consistency of the concentrations of each metabolite indicates that steady state has been reached (the time derivatives are provided in supplementary Notebook S1). Second, the concentrations of metabolites span a range from 10^−22^*M* for mitochondrial oxaloacetate at the low end to 10^6^*M* for fructose 1,6-bisphosphate and 10^9^*M* for mitochondrial acetyl CoA at the high end. While a concentration of 10^−22^*M* might be reasonable in a cell (effectively zero concentration in a typical cell with a volume of 10^−15^ − 10^−12^ liters), concentrations above 10^−3^*M* to 10^−2^*M* are usually not physiologically realistic [25]. For instance, if fructose 1,6-bisphosphate had a concentration much above 10^−3^*M*, the cytoplasm could become so glassy that diffusion of even small molecules would decrease below the level at which metabolism could operate.

However, the issue is not whether the maximum entropy production rate formulation is appropriate for biological systems, but rather that any model is always a reduced representation of a real system [16]. In a complete model of cellular metabolism, diffusion would be included and the maximum entropy production rate solution would balance the rate of diffusion with the tendency for any chemical species to move towards its thermodynamically optimal distribution.

This discrepancy between predicted metabolite concentrations and physiological expectations can be taken advantage of, however, to infer points of post-translational enzyme regulation. To do this, a loss function is formulated (Equation (8)), similar to that used in machine learning approaches, to identify nodes in the system (in this case, reactions or enzymes) that need to be adjusted or regulated in order to match observed results. We applied Equation (8) to the simulation predictions for each reaction using a rule of thumb that physiological concentrations should not exceed 10^−3^*M*. The results are shown in Table 1. Twelve reactions have product concentrations higher than expected. In choosing which reactions to apply regulation to, we take a parsimonious approach based on two principles: (1) regulation of upstream enzymes should have precedence over regulation of downstream enzymes; and (2) the reactions with the highest loss-function value should be evaluated before those with lower loss function values. Using these principles, we chose to apply regulation to only phosphofructokinase (PFK) and mitochondrial pyruvate dehydrogenase (PDHm), which are the two reactions with the highest loss-function values. These are also the two reactions that are most commonly regulated in glycolysis.

The loss function does not help to identify how these reactions/enzymes might be regulated. In experimental studies of *Neurospora crassa*, PFK in *Neurospora crassa* is inhibited by high concentrations of ATP [36]. Early studies on Neurospora PDHm indicated that PDHm is regulated by acetyl-CoA and covalently by phosphorylation [37,38]. We chose to regulate the PFK reaction by ATP concentration and the PDHm reaction by acetyl-CoA concentration using Hill equations.

**Figure 3.**
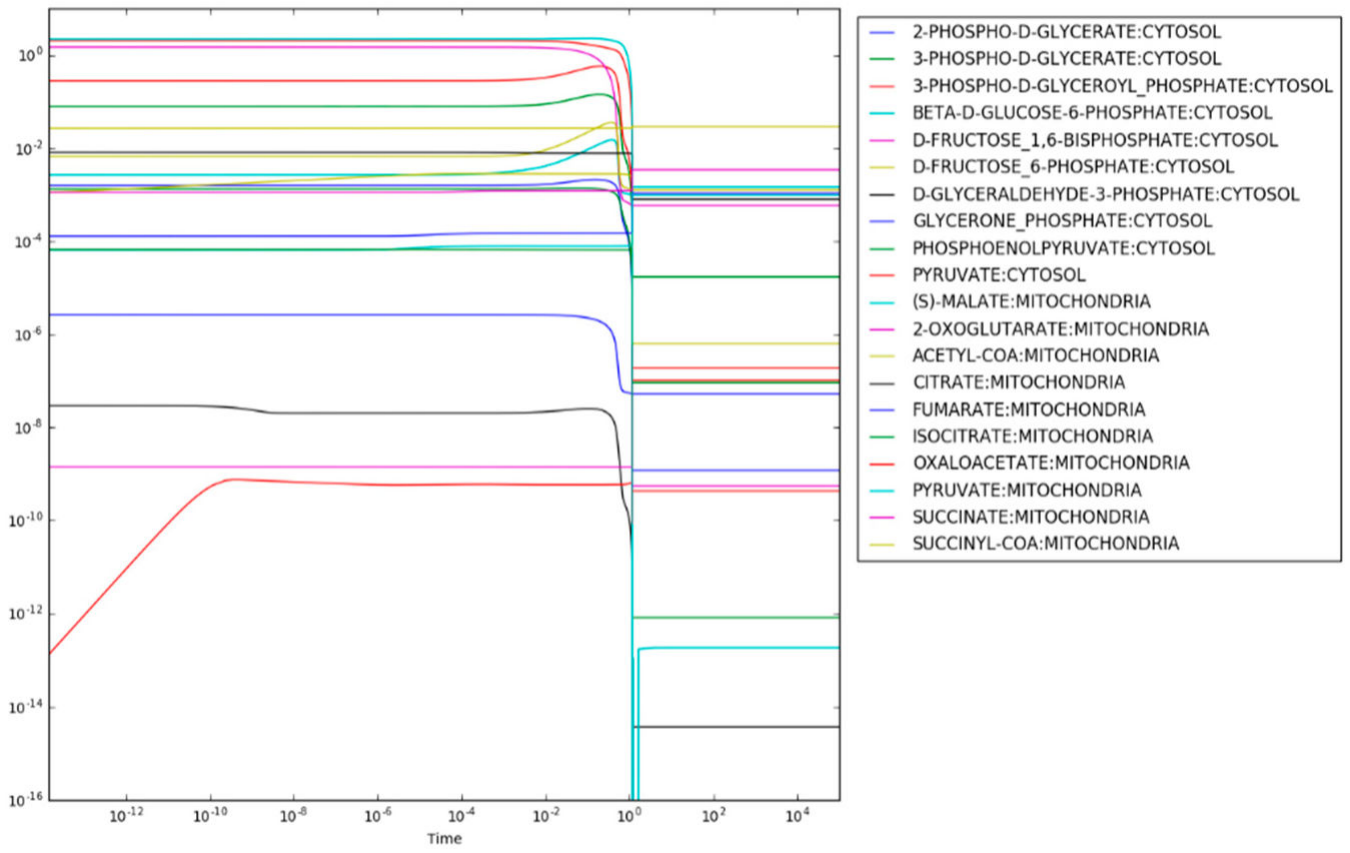
Concentrations as a function of time in maximum entropy optimization with regulation.

After regulation of PFK and PDHm are applied, the maximum entropy optimization produces the steady state concentrations shown in Figure 3. Most metabolite concentrations fall within the rule-of-thumb values (≤ 10^−3^*M*) and reanalysis of the loss function for each reaction finds that only the succinyl-CoA synthetase reaction resulting in the production of succinate has a loss-function value, *L* = 3.8 above the expected value of 0.0. Given that succinate is highly soluble, however, this mild increase of the loss function is reasonable. The predicted concentration of succinate is 3.4 mM. For comparison, the concentration of succinate in exponentially growing *E. coli* under similar conditions was estimated to be 0.57 mM [25].

In addition to changes in the metabolite concentrations, application of regulation to PFK and PDHm results in reduced flux through both glycolysis and the TCA cycle, as expected. In this case, the flux is reduced by just over 2-fold (6.53/2.86).

With reasonable steady-state metabolite concentrations in hand, rate constants can be inferred from the simulation using Equation (9). The resulting rate constants are provided in supplementary Notebook S2. The inferred rate constants are not generally comparable to rate parameters for the analogous reactions determined from in vitro enzyme kinetic studies, however, since in vitro studies characterize reactions using Michaelis–Menten equations and the simulation studies reported here do not explicitly model enzyme kinetics. However, the flux values from the simulation are consistent with inferred fluxes from metabolic flux analysis studies on the related fungus *Yarrowia lipolytica* [39], which has a very similar central metabolism based on genome analysis and modeling [32,40-42].

**Figure 4.**
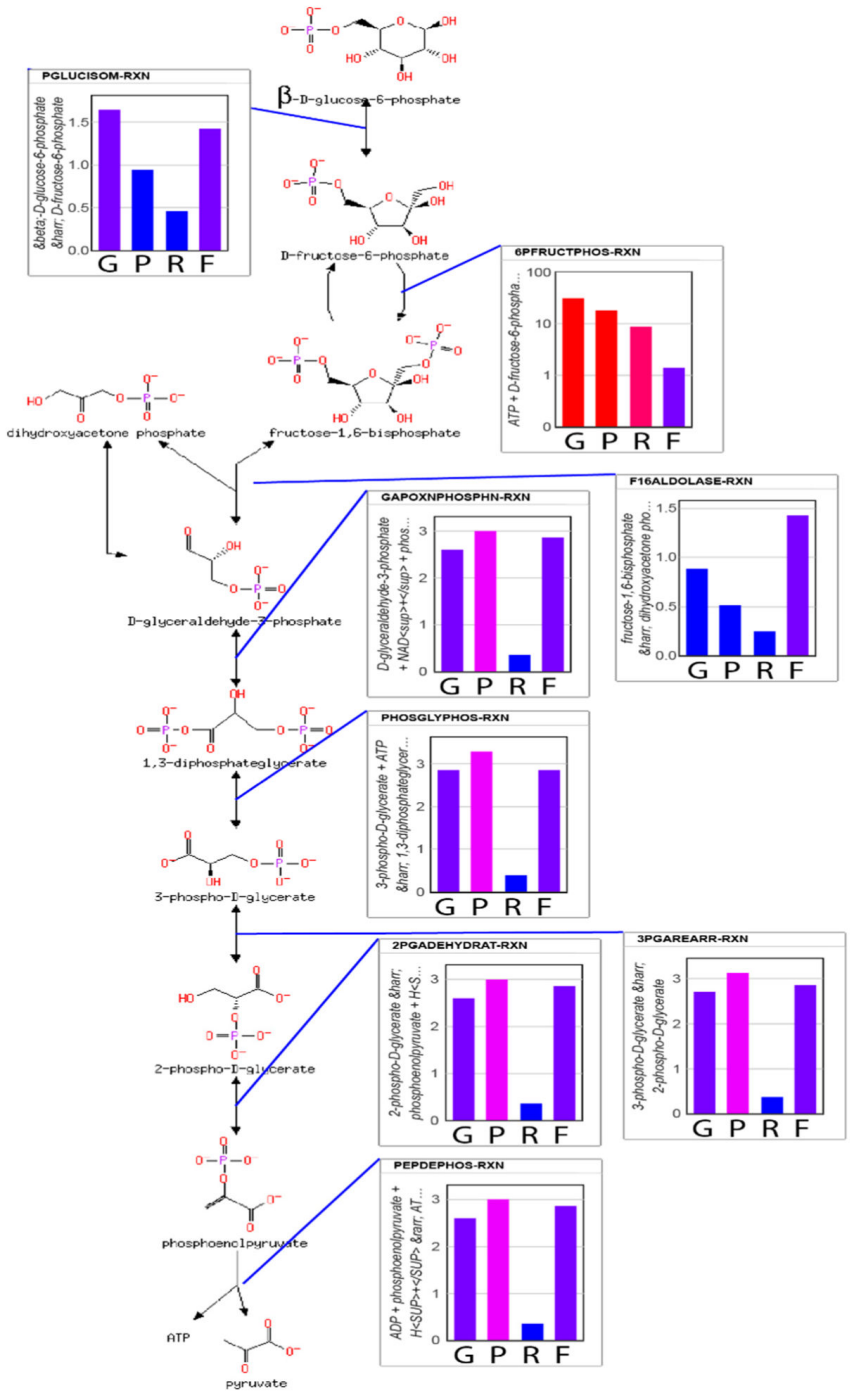
Energetics of glycolysis reactions. Columns from left to right indicate: (G) −Δ*G_rxn_*, (P) power, (R) resistance, and (F) flux. Red indicates high values and blue indicates low values. Values are in arbitrary, relative units but the specific values are provided in supplementary Notebook_S3. The phosphofructokinase reaction has dramatically different characteristics than the other reactions because feedback regulation of ATP turns it into a potentiometer. The metabolic pathway visualization was created with Pathway Tools [44].

From the predicted flux values and reaction-free energies, the power characteristics and resistance of each reaction can be estimated. Shown in Figure 4 are relative values of the (1) reaction free energies; (2) power at each reaction; (3) reaction resistance; and (4) reaction flux for each reaction of glycolysis. Since lower glycolysis and the TCA cycle (supplementary Notebook S3) have identical net fluxes at steady state and similar reaction-free energies, the power characteristics and conductances of these reactions are very similar. The power generated at the PFK reaction is 6-fold higher than the power generated at other reactions of glycolysis. However, while the phosphorylation of fructose 6-phosphate to the highly soluble fructose 1,6-bisphosphate contributes significantly to the overall free energy change of the combined glycolysis-TCA pathway, if the system were allowed to proceed to the maximum entropy distribution (that is, without regulation), all reactions would have very similar power characteristics. It is the applied regulation that keeps the thermodynamic driving force on the PFK reaction much higher than that for the other reactions. Consequently, the chemical resistance at PFK is much higher than for other reactions, as well. Due to the regulation, both the flux through upper glycolysis and the flux through lower glycolysis and the TCA cycle are reduced more than 2-fold. While it has long been stated that PFK is regulated to modulate the flow of material through glycolysis, the simulations suggest that the more nuanced explanation is that regulation acts as a potentiometer to modulate both the flow of material through the reaction and the accumulation of fructose 1,6-bisphosphate in the system. Regardless, flow of material through glycolysis to the TCA cycle is reduced significantly. However, if the causal explanation for the regulation were solely to reduce the flux through the pathway, this would unnecessarily reduce the ability of the organisms using this metabolism to compete since energy production would slowed down significantly, as well, which is counter to Lotka’s early conjecture.

Rather than simply not extracting this available energy from glucose at a high rate due to already high levels of ATP, it is reasonable to expect that the energy from glucose is utilized elsewhere. Proteomics studies on Neurospora show that the enzymes of upper glycolysis and the oxidative pentose phosphate pathway oscillate with the circadian cycle but are 180° out of phase with each other [21]. Consequently, simulations of glycolysis, the TCA cycle and the pentose phosphate pathway were carried out following the same steps as described above: (1) maximum entropy production rate optimization without regulation of the pentose phosphate pathway followed by (2) inference of regulation and (3) re-optimization with regulation to obtain steady state metabolite levels. Evaluation of the loss functions for each reaction in the pentose phosphate pathway predicted that glucose-6-phosphate dehydrogenase and phosphogluconolactonase, the first and second steps of the pentose phosphate pathway, should additionally be regulated. Glucose-6-phosphate dehydrogenases are well-known to be regulated by NADP/NADPH, either directly or indirectly, and by phosphorylation [43]. No literature was found regarding post-translational regulation of phosphogluconolactonase. Accordingly, we added regulation to the glucose-6-phosphate dehydrogenase reaction but not to the phosphogluconolactonase reaction. The glucose-6-phosphate dehydrogenase reaction was again regulated by a Hill equation based on the levels of NADPH. We evaluated the kinetics of the system (both oxidative and non-oxidative) under three conditions which differed by the NADP/NADPH ratio. An initial low ratio of NADP/NADPH was taken from an isotope labeling, mass spectrometry analysis of the exponential growth of *E. coli* in which NADP/NADPH = 2.1 · 10^−6^/1.2 · 10^−4^ = 0.0175 [25]. This ratio results in a mild driving force on the NADP/NADPH-dependent reactions of the oxidative branch of the pentose phosphate pathway (enzymes glucose-6-dehydrogenase and phosphogluconate dehydrogenase) of ~−2.7 KJ/mol. A moderate ratio of NADP/NADPH was taken to be NADP/NADPH = 1, resulting in driving forces on the latter reactions of ~−3.6 KJ/mol. A high ratio was taken to be the observed values for NAD/NADH in the *E. coli* study such that NADP/NADPH = 2.6 · 10^−3^/8.3 · 10^−5^ = 31, resulting in a driving force of ~−31.2 KJ/mol on these same dehydrogenase reactions.

**Figure 5.**
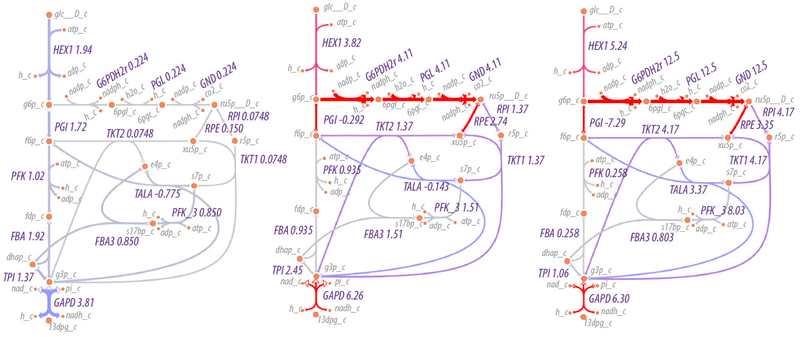
Reaction flux through upper glycolysis and the pentose phosphate pathway as a function of the NADP/NADPH ratio. (**Left**) Low values of the ratio combined with high values of ATP result in approximately equal flow of material through upper glycolysis and the non-oxidative branch of the pentose phosphate pathway, minimizing the production of fructose 1,6-bisphosphate; (**Middle**) a NADP/NADPH ratio of 1 results in flow through each of upper glycolysis, non-oxidative and oxidative pentose phosphate pathways, with the oxidative pentose phosphate pathway containing approximately 65% of the flow of material; (**Right**) a high value of the ratio results in the cycling of flow iteratively through the oxidative pentose phosphate pathway while flow through upper glycolysis is minimal.

As expected, the flow of material increases through the pentose phosphate pathway as the NADP/NADPH ratio increases, as shown in Figure 5. At relatively low to moderate ratios of NADP/NADPH, the flow of material is mostly through the non-oxidative branch of the pentose phosphate pathway, while at high ratios of NADP/NADPH, flow through the oxidative branch is maximized, with an average of three cycles through the oxidative pentose phosphate branch for each glucose utilized (the simulation conditions are under non-growth conditions, so pentose phosphates are not drawn off for biosynthetic purposes). That is, rather than acting as a linear pathway from glucose-6-phosphate to glyceraldehyde-3-phosphate, the pentose phosphate pathway acts cyclically to maximize production of NADPH. The overall reaction of the pentose phosphate pathway at high ratios of NADP/NADPH is,

$$\;\text{Glucose}\;6-\text{phosphate}+6\;{\text{NADP}}^{+}+3H_{2}O⇌\;\text{glyceraldehyde}\;3-\text{phosphate}+6\;\text{NADPH}+3{\text{CO}}_{2}.$$

## 4. Discussion

The maximum entropy principle can be used to predict concentrations, obtain optimal rate constants, and calculate the characteristics of biological circuits including energy, power, flux and resistance for individual reactions and entire pathways. In the model of central metabolism used here, enzyme kinetics are represented as the summary reaction of the catalytic process. Dobovišek et al. have shown that the maximum entropy production principle can be used to evaluate enzyme kinetics, as well [45,46]. In this study, for convenience the enzyme catalysts are not explicitly represented in the model except for regulation. It is entirely feasible to do so, however, even without the use of the assumptions built into Michaelis–Menten model that the system be at steady state and far from equilibrium.

The calculated rate constants are optimal for the growth conditions used in the simulation. Similarly, organisms will have rate constants that are optimal for the conditions to which they have adapted. Ideally, to compare predicted metabolite concentrations to those from a wet lab experiment, the organism should be cultured over many generations to ensure adaptation to the laboratory conditions.

One could calculate experimentally based in vivo rate constants for many reactions, as well, following the same procedure outlined above but using experimentally measured concentrations of metabolites free (unbound) in the cytoplasm [18]. But this population is challenging to measure [47] or even estimate as a trend [25]. If such metabolite measurements were available, the hypothesis of maximum entropy production for steady states of evolutionary optimized systems is testable [48].

Park et al. have recently argued that one can calculate free energies of reaction from the respective whole cell concentrations because most cellular metabolites are in free form [49]. The argument is that the total measured metabolite concentration is approximately 300 mM, whereas the protein concentration is approximately 7 mM, and that this suggests that most metabolites are free in solution. However, this argument can be turned around to argue the opposite as well. For example, if three metabolites have concentrations of 100 mM and other species are 1 nM–1 μM, then the pooled concentration of all metabolites may be ~300 mM, depending on the number of other metabolic species. Further suppose that the total pooled protein concentration was 7 mM. In this case, only the three former species may have a significant population free in the cytoplasm. In order to determine the concentration of each free or unbound metabolite experimentally, we need either new experimental isolation technologies or to use predictions such as those discussed herein, but on the genome scale, as prior information in the data analysis. Measured whole-cell population of metabolites can be used to set bounds on reaction-free energies, however.

Using measured whole-cell concentrations of metabolites as the upper bound for metabolites free in solution can also be employed to infer regulation rather than using a rule-of-thumb (e.g., that metabolite concentrations should not exceed 1 mM), as was done here. The experimentally determined concentrations would give more precise estimates of regulation, although the rule-of-thumb values worked well in this study for demonstration purposes.

It is assumed that any predicted concentrations far away from the data are due to the incompleteness of the model and not the maximum entropy production principle. In the cases studied here, it is clear that the predicted high levels of some metabolites would reduce the ability of molecules to diffuse in the cell. In cases such as this, regulation may be inferred by comparison to experimental data or expectations. But it can also be expected that branching reactions for metabolites that are not included in a model may impact the predictions. Branching per se at the point where the metabolite is an intermediate does not necessarily impact metabolite concentrations because the metabolite level is a function of the inward and outward flux producing and consuming the metabolite. The total inward and outward flux may not change when there is a branching reaction if the boundary conditions have not changed; in this case, the output flux splits but the total outward flux may be conserved.

Likewise, changing the boundary conditions would change the overall flux through all pathways. Figure 5 shows the effect on flux through the pentose phosphate pathway as a function of the boundary conditions for NADP and NADPH. The flux through the pentose phosphase pathway changes by 55-fold (12.5/0.224). However, the fluxes in this case are modulated by regulation at the first step in the pathway, the reaction for glucose 6-phosphate dehydrogenase. The corresponding simulations without regulation would change the flux by much more. Small changes in boundary conditions can result in significantly greater flux through pathways because the boundary conditions in this case include internal metabolites as well as external nutrients/waste products. Because the internal metabolites such as NADP/NADPH, NAD/NADH and ATP/(ADP+P_i_) may be used in many reactions, the impact on flux can be multiplicative.

The ability to completely characterize each individual reaction with regard to flux, energy dissipation, power and resistance can also lead to a more complete characterization of biological circuits, as is done for electrical circuits, and even allow for the use of sophisticated control theory analyses of the operations of a cell. Such developments could be extremely useful for synthetic biology, in that the addition to a cell of an engineered circuit often results in decreased growth and other unintended consequences. Being able to fully characterize the impact of the new circuit before implementing it in the laboratory could dramatically change the design process and success rate.

For example, in an effort to increase the production of fatty acids in *Yarrowia lipolytica*, Wasylenko et al. used 13C-metabolic flux analysis (MFA) in a control strain and an engineered strain to understand where NADPH was primarily being produced, which turned out to be the oxidative pentose phosphate pathway [39]. MFA is a valuable but relatively costly experimental analysis. In comparison, this study used predictive simulations and found that the oxidative branch can act in a cyclical manner to iteratively produce high levels of NADPH. Routinely carrying out modeling studies such as this is promising for bringing more predictive rational design into the development cycle.

Finally, there is a need for faster optimization methods to reach the steady state. While optimization using Equation (6) will find the optimal (least heat dissipation) steady state, the ODEs can become particularly stiff when regulation is not applied. In this case, it is possible for the range of concentrations to vary by many orders of magnitude, leading to very stiff ODEs and long time-to-solution. This may be important as these methods are used to model secondary metabolism as well as to scale up to a genome-scale model, which we plan to do for *Neurospora crassa*.

Scaling up to a genome-scale model will likely bring new challenges. The time scale of metabolism spans the range from milliseconds for individual reactions to on the order of an hour for secondary metabolites to reach steady state. The assumption in Equation (6) that *c_α_* is reaction-independent allows one to find the maximum entropy production distribution at a non-equilibrium steady state. However, one concern is that this would imply that all reactions in the eventual simulation would occur on the same timescale. If all the processes are represented at the same level, for instance that of elementary reactions, then the inferred rates will likely be sufficiently representative of the true processes. However, if one mixes elementary reactions and summary reactions (that represent larger processes) in the model, then it could be the case that there is not a realistic separation of time scales. Summary reactions representing larger processes, for instance, could be rescaled by *nc_α_* instead of *c_α_*, where *c_α_* represents the average transit time through a reaction at the faster scale and *n* is the number of elementary reactions represented by the summary reaction. Multi-scale simulations can be used in which fast and slow processes are run independently based on the assumption that the fast processes reach steady state quickly on the time scale of the slow processes. If the processes to be represented are scaled appropriately, for instance by *nc_α_*, the processes do not need to be run independently but can be run together using computational singular perturbation methods [50] or other approaches that automatically separate the fast and slow degrees of freedom [51]. Further developments in the methods for inferring regulation, however, will likely be required. Including biomass formation in the form of replication will be challenging, as it will require a chemical potential for biomass to be estimated.

## Supplementary Material

NotebookS1

NotebookS2

NotebookS3

TableS1

TableS2

## Figures and Tables

**Table 1.** Product of the reaction product concentrations for the optimization predictions and expected values, and resulting value of the loss function L (Equation (8)). Full common names are provided as supplementary Table S2.

Product of Concentrations			
Reaction	Predicted	Expected	*L*
CSm	4.85 × 10^−6^	1.00 × 10^−6^	1.58
SUCOASm	6.62 × 10^−9^	1.00 × 10^−9^	1.89
ENO	6.65 × 10^−1^	1.00 × 10^−3^	6.50
PGM	1.33	1.00 × 10^−3^	7.19
HEX1	4.26 × 10^−2^	1.00 × 10^−6^	10.66
PGI	4.58 × 10^1^	1.00 × 10^−3^	10.73
GAPD	4.82 × 10^−2^	1.00 × 10^−6^	10.78
PYRt2m	8.43 × 10^2^	1.00 × 10^−3^	13.64
PGK	1.09	1.00 × 10^−6^	13.90
PYK	5.84	1.00 × 10^−6^	15.58
PFK	9.21 × 10^2^	1.00 × 10^−6^	20.64
PDHm	8.66	1.00 × 10^−9^	22.88
