# Supplementary material for: Prediction of Metabolite Concentrations, Rate Constants and Post-Translational Regulation Using Maximum Entropy-Based Simulations with Application to Central Metabolism of Neurospora crassa
Source: Processes (Basel). Author manuscript; Available in PMC 2021 Apr 5. (PMC8020867; doi:10.3390/pr6060063)
Supplement: NotebookS1 [file NIHMS1061468-supplement-NotebookS1.pdf]

Table of Contents

1 Boltzmann Simulation
1.1 Pentose phosphate + Glycolysis + TCA cycle
2 Models and Simulations
2.1 Model Without Regulation
2.1.1 Run Deterministic Simulation
2.1.2 Derivatives from ODE thermodynamic optimization simulation
2.1.3 Thermodynamic Odds of Reaction from Deterministic Simulation
2.1.4 Calculate Net Odds
2.1.5 Map Net Likelihoods onto Metabolic Pathways
2.1.6 Find Active and Variable Metabolites
2.1.7 Time Derivatives of Metabolite Concentration: S · Net Odds = flux
2.1.8 Analyze Variable Metabolite Levels from Deterministic Simulation
2.2 Infer Regulated Reactions

In [37]:
```
import numpy as np
import pandas as pd
import matplotlib.pyplot as plt
import matplotlib
import subprocess
import re
import os
from IPython.core.display import display
pd.set_option('display.max_columns', None,'display.max_rows', None)
#from ipynb_latex_setup import *
%matplotlib inline
#%matplotlib pdf
#matplotlib.matplotlib_fname()
#import matplotlib.rcsetup as rcsetup
#print(rcsetup.all_backends)
T = 298.15
R = 8.314e-03
RT = R*T
N_avogadro = 6.022140857e+23
VolCell = 1.0e-15
Concentration2Count = N_avogadro * VolCell
concentration_increment = 1/(N_avogadro*VolCell)
```

Change directories to where the simulation data is:

In [38]:
```
# Comment/uncomment to change to your directory, if needed:
cwd = os.getcwd()
mydir = cwd+'/simulation_data'
if 'simulation_data' not in mydir:
  os.chdir(mydir)
```

Boltzmann Simulation

A beta version of the simulation code is available on GitHub at https://github.com/PNNL-CompBio/Boltzmann (https://github.com/PNNL-CompBio/Boltzmann).

Pentose phosphate + Glycolysis + TCA cycle

Model input file (Reactions):

In [39]:
```
cat neurospora_pentose_phos.glycolysis.tca.2.dat
```

```
REACTION ME1m
LEFT    (S)-MALATE + NAD+
RIGHT   pyruvate + NADH + CO2
LEFT_COMPARTMENT MITOCHONDRIA
RIGHT_COMPARTMENT MITOCHONDRIA
ENZYME_LEVEL    0.0
//
REACTION ME2m
LEFT    (S)-MALATE + NADP+
RIGHT   PYRUVATE + NADPH + CO2
LEFT_COMPARTMENT MITOCHONDRIA
RIGHT_COMPARTMENT MITOCHONDRIA
ENZYME_LEVEL    0.0
//
REACTION CSm
LEFT    OXALOACETATE + ACETYL-COA + H2O
RIGHT   CITRATE + COA
LEFT_COMPARTMENT MITOCHONDRIA
RIGHT_COMPARTMENT MITOCHONDRIA
COMMENT PH = 7.0, IONIC STRENGTH = 0.15 M
//
REACTION ACONTm
LEFT    CITRATE
RIGHT   ISOCITRATE
LEFT_COMPARTMENT MITOCHONDRIA
RIGHT_COMPARTMENT MITOCHONDRIA
COMMENT  PH = 7.0, IONIC STRENGTH = 0.15 M
COMMENT DOn't split into two reactions if max entropy is being used
- only one enzyme needs to be epressed
//
REACTION ICDHxm
LEFT    ISOCITRATE + NAD+
```

```
RIGHT   2-OXOGLUTARATE + NADH + CO2
LEFT_COMPARTMENT MITOCHONDRIA
RIGHT_COMPARTMENT MITOCHONDRIA
COMMENT PH = 7.0, IONIC STRENGTH = 0.15 M
//
REACTION AKGDm
LEFT    2-OXOGLUTARATE + COA + NAD+
RIGHT   SUCCINYL-COA + CO2 + NADH
LEFT_COMPARTMENT MITOCHONDRIA
RIGHT_COMPARTMENT MITOCHONDRIA
COMMENT PH = 7.0, IONIC STRENGTH = 0.15 M
//
REACTION SUCOASm
LEFT    SUCCINYL-COA + ADP + Orthophosphate
RIGHT   SUCCINATE + ATP + COA
LEFT_COMPARTMENT MITOCHONDRIA
RIGHT_COMPARTMENT MITOCHONDRIA
COMMENT PH = 7.0, IONIC STRENGTH = 0.15 M
//
REACTION SUCD1m
LEFT    SUCCINATE + redox1
RIGHT   FUMARATE + redox2
LEFT_COMPARTMENT MITOCHONDRIA
RIGHT_COMPARTMENT MITOCHONDRIA
COMMENT PH = 7.0, IONIC STRENGTH = 0.15 M
//
REACTION FUMm
LEFT    FUMARATE + H2O
RIGHT   (S)-MALATE
LEFT_COMPARTMENT MITOCHONDRIA
RIGHT_COMPARTMENT MITOCHONDRIA
COMMENT PH = 7.0, IONIC STRENGTH = 0.15 M
//
REACTION  MDHm
LEFT    (S)-MALATE + NAD+
RIGHT   OXALOACETATE + NADH
LEFT_COMPARTMENT MITOCHONDRIA
RIGHT_COMPARTMENT MITOCHONDRIA
COMMENT PH = 7.0, IONIC STRENGTH = 0.15 M
//
REACTION GAPD
LEFT    D-GLYCERALDEHYDE-3-PHOSPHATE + ORTHOPHOSPHATE + NAD+
RIGHT   3-Phospho-D-glyceroyl_phosphate + NADH
ENZYME_LEVEL    1.0
LEFT_COMPARTMENT CYTOSOL
RIGHT_COMPARTMENT CYTOSOL
PATHWAY GLUCONEOGENESIS, GLYCOLYSIS
COMMENTS  From Dennis
//
REACTION PGK
LEFT    3-Phospho-D-glyceroyl_phosphate + ADP
RIGHT   3-PHOSPHO-D-GLYCERATE + ATP
LEFT_COMPARTMENT CYTOSOL
```

```
RIGHT_COMPARTMENT CYTOSOL
ENZYME_LEVEL    1.0
PATHWAY GLUCONEOGENESIS, GLYCOLYSIS, CALVIN CYCLE
COMMENTS  From Dennis
//
REACTION TPI
LEFT    Glycerone_phosphate
RIGHT   D-GLYCERALDEHYDE-3-PHOSPHATE
LEFT_COMPARTMENT CYTOSOL
RIGHT_COMPARTMENT CYTOSOL
ENZYME_LEVEL    1.0
COMMENT From Equilibrator
PATHWAY CALVIN CYCLE, GLYCOLYSIS
//
REACTION  MDH
LEFT    (S)-MALATE + NAD+
RIGHT   OXALOACETATE + NADH
LEFT_COMPARTMENT CYTOSOL
RIGHT_COMPARTMENT CYTOSOL
ENZYME_LEVEL    0.0
COMMENT PH = 7.0, IONIC STRENGTH = 0.15 M
COMMENT Cytosolic
//
REACTION PEP_Carboxylase
LEFT    oxaloacetate + orthophosphate
RIGHT   phosphoenolpyruvate + CO2 + H2O
LEFT_COMPARTMENT CYTOSOL
RIGHT_COMPARTMENT CYTOSOL
ENZYME_LEVEL    0.0
PATHWAY GLUCONEOGENSESIS
COMMENTS
//
REACTION PPCK
LEFT    oxaloacetate + ATP
RIGHT   phosphoenolpyruvate + ADP + CO2
LEFT_COMPARTMENT CYTOSOL
RIGHT_COMPARTMENT CYTOSOL
ENZYME_LEVEL    0.0
PATHWAY GLUCONEOGENSESIS
COMMENTS
//
REACTION FBA
LEFT    D-FRUCTOSE_1,6-BISPHOSPHATE
RIGHT   Glycerone_phosphate + D-GLYCERALDEHYDE-3-PHOSPHATE
LEFT_COMPARTMENT CYTOSOL
RIGHT_COMPARTMENT CYTOSOL
ENZYME_LEVEL    1.0
PATHWAY GLUCONEOGENSESIS, GLYCOLYSIS, CALVIN CYCLE
COMMENTS  From Dennis
//
REACTION FBP
LEFT    D-FRUCTOSE_6-PHOSPHATE + Orthophosphate
RIGHT   H2O + D-FRUCTOSE_1,6-BISPHOSPHATE
```

```
LEFT_COMPARTMENT CYTOSOL
RIGHT_COMPARTMENT CYTOSOL
ENZYME_LEVEL    0.0
PATHWAY GLUCONEOGENESIS, CALVIN CYCLE
COMMENTS  From Dennis
//
REACTION TKT2
LEFT    D-FRUCTOSE_6-PHOSPHATE + D-GLYCERALDEHYDE-3-PHOSPHATE
RIGHT   D-ERYTHROSE-4-PHOSPHATE + D-XYLULOSE-5-PHOSPHATE
LEFT_COMPARTMENT CYTOSOL
RIGHT_COMPARTMENT CYTOSOL
ENZYME_LEVEL    0.0
PATHWAY CALVIN CYCLE, PENTOSE PHOSPHATE PATHWAY, RUBISCO SHUNT
COMMENT   From Equilibrator pH 7.5, IS 0.15
//
REACTION RPE
LEFT    D-XYLULOSE-5-PHOSPHATE
RIGHT   D-RIBULOSE-5-PHOSPHATE
LEFT_COMPARTMENT CYTOSOL
RIGHT_COMPARTMENT CYTOSOL
ENZYME_LEVEL    0.0
PATHWAY CALVIN CYCLE, PENTOSE PHOSPHATE PATHWAY, RUBISCO SHUNT
COMMENT  From Dennis
//
REACTION Xylulokinase
LEFT    D-XYLULOSE + ATP
RIGHT   D-XYLULOSE-5-PHOSPHATE + ADP
LEFT_COMPARTMENT CYTOSOL
RIGHT_COMPARTMENT CYTOSOL
ENZYME_LEVEL    0.0
PATHWAY CALVIN CYCLE, PENTOSE PHOSPHATE PATHWAY, RUBISCO SHUNT
COMMENT
//
REACTION PYK_org
LEFT    ADP + PHOSPHOENOLPYRUVATE
RIGHT   PYRUVATE + ATP
LEFT_COMPARTMENT CYTOSOL
RIGHT_COMPARTMENT CYTOSOL
ENZYME_LEVEL    0.0
PATHWAY GLYCOLYSIS, PYRUVATE METABOLISM, RUBISCO SHUNT
COMMENT  From Dennis
//
REACTION PYK
LEFT    ADP + PHOSPHOENOLPYRUVATE
RIGHT   PYRUVATE + ATP
LEFT_COMPARTMENT CYTOSOL
RIGHT_COMPARTMENT CYTOSOL
ENZYME_LEVEL    1.0
PATHWAY GLYCOLYSIS, PYRUVATE METABOLISM, RUBISCO SHUNT
COMMENT  From Dennis
//
REACTION RPI
LEFT    D-RIBOSE-5-PHOSPHATE
```

```
RIGHT   D-RIBULOSE-5-PHOSPHATE
LEFT_COMPARTMENT CYTOSOL
RIGHT_COMPARTMENT CYTOSOL
ENZYME_LEVEL    0.0
PATHWAY CALVIN CYCLE, PENTOSE PHOSPHATE PATHWAY, RUBISCO SHUNT
COMMENT  From Dennis
//
REACTION TKT1
LEFT    SEDOHEPTULOSE_7-PHOSPHATE + D-GLYCERALDEHYDE-3-PHOSPHATE
RIGHT   D-RIBOSE-5-PHOSPHATE + D-XYLULOSE-5-PHOSPHATE
LEFT_COMPARTMENT CYTOSOL
RIGHT_COMPARTMENT CYTOSOL
ENZYME_LEVEL    0.0
PATHWAY CALVIN CYCLE, PENTOSE PHOSPHATE PATHWAY, RUBISCO SHUNT
COMMENT  From Equilibrator pH 7.5, IS 0.15
//
REACTION TALA
LEFT    D-GLYCERALDEHYDE-3-PHOSPHATE + SEDOHEPTULOSE_7-PHOSPHATE
RIGHT   D-FRUCTOSE_6-PHOSPHATE + D-ERYTHROSE-4-PHOSPHATE
LEFT_COMPARTMENT CYTOSOL
RIGHT_COMPARTMENT CYTOSOL
ENZYME_LEVEL    0.0
PATHWAY  RUBISCO SHUNT, PENTOSE PHOSPHATE PATHWAY
COMMENT  From Equilibrator pH 7.5 IS 0.15
//
REACTION PGM
LEFT    3-PHOSPHO-D-GLYCERATE
RIGHT   2-PHOSPHO-D-GLYCERATE
LEFT_COMPARTMENT CYTOSOL
RIGHT_COMPARTMENT CYTOSOL
ENZYME_LEVEL    1.0
PATHWAY  GLUCONEOGENESIS, RUBISCO SHUNT, GLYCOLYSIS I
COMMENTS  From Dennis
//
REACTION  ENO
LEFT    2-PHOSPHO-D-GLYCERATE
RIGHT   PHOSPHOENOLPYRUVATE + H2O
LEFT_COMPARTMENT CYTOSOL
RIGHT_COMPARTMENT CYTOSOL
ENZYME_LEVEL    1.0
PATHWAY  GLUCONEOGENESIS, RUBISCO SHUNT, GLYCOLYSIS I
COMMENTS  From Dennis
//
# Pentose Phosphate reactions (oxidative branch):
REACTION GND
LEFT  NADPH + D-RIBULOSE-5-PHOSPHATE + CO2
RIGHT NADP+ + 6-PHOSPHO-D-GLUCONATE
LEFT_COMPARTMENT CYTOSOL
RIGHT_COMPARTMENT CYTOSOL
PATHWAY PENTOSE PHOSPHATE PATHWAY
ENZYME_LEVEL    0.0
//
REACTION  PGL
```

```
LEFT  6-PHOSPHO-D-GLUCONATE
RIGHT D-Glucono-1,5-lactone_6-phosphate + H2O
LEFT_COMPARTMENT CYTOSOL
RIGHT_COMPARTMENT CYTOSOL
COMMENT From Dennis
PATHWAY PENTOSE PHOSPHATE PATHWAY
ENZYME_LEVEL    0.0
//
REACTION HEX1
LEFT    BETA-D-GLUCOSE + ATP
RIGHT   BETA-D-GLUCOSE-6-PHOSPHATE + ADP
LEFT_COMPARTMENT CYTOSOL
RIGHT_COMPARTMENT CYTOSOL
NREGULATION ATP 2.80e-03 10
ENZYME_LEVEL    1.0
PATHWAY GLUCONEOGENESIS, (GLYCOLYSIS-YEAST), GLYCOLYSIS
COMMENTS  From Dennis
//
REACTION PGI
LEFT    BETA-D-GLUCOSE-6-PHOSPHATE
RIGHT   D-FRUCTOSE_6-PHOSPHATE
LEFT_COMPARTMENT CYTOSOL
RIGHT_COMPARTMENT CYTOSOL
ENZYME_LEVEL    1.0
PATHWAY GLUCONEOGENESIS, (GLYCOLYSIS-YEAST), GLYCOLYSIS
COMMENTS  From Dennis
//
# Gluconeogenesis reactions :
REACTION  G6PDH2r
LEFT    BETA-D-GLUCOSE-6-PHOSPHATE + NADP+
RIGHT   D-Glucono-1,5-lactone_6-phosphate + NADPH
LEFT_COMPARTMENT CYTOSOL
RIGHT_COMPARTMENT CYTOSOL
ENZYME_LEVEL    0.0
COMMENT  From Dennis
PATHWAY GLUCONEOGENESIS
//
# Glycolysis reactions :
REACTION  PFK
LEFT    D-FRUCTOSE_6-PHOSPHATE + ATP
RIGHT   ADP + D-FRUCTOSE_1,6-BISPHOSPHATE
LEFT_COMPARTMENT CYTOSOL
RIGHT_COMPARTMENT CYTOSOL
NREGULATION ATP 2.80e-03 10
ENZYME_LEVEL    1.0
PATHWAY GLYCOLYSIS
COMMNENT From Equilibrator
//
REACTION PYRt2m
LEFT    PYRUVATE
RIGHT   PYRUVATE
DGZERO  -5.94
COMMENT -5.94
```

```
DGZERO-UNITS    KJ/MOL
LEFT_COMPARTMENT CYTOSOL
RIGHT_COMPARTMENT MITOCHONDRIA
ENZYME_LEVEL    1.0
COMMENT PYRUVATE TRANSPORT INTO MITOCHONDRIA - Assuming free energy
difference = \Delta -RT \Delta pH = -RT log 10
//
# Acetyl CoA synthesis
REACTION PDHm
LEFT    COA + NAD+ + PYRUVATE
RIGHT   ACETYL-COA + CO2 + NADH
LEFT_COMPARTMENT  MITOCHONDRIA
RIGHT_COMPARTMENT MITOCHONDRIA
ENZYME_LEVEL    1.0
NREGULATION ACETYL-COA 1.00e-03 20
PATHWAY ACETYL COA BIOSYNTHESIS
COMMENT PYRUVATE DEHYDROGENASE SUMMARY REACTION
//
REACTION ICL
LEFT isocitrate
RIGHT   glyoxylate + succinate
LEFT_COMPARTMENT GLYOXYSOME
RIGHT_COMPARTMENT GLYOXYSOME
ENZYME_LEVEL    0.0
PATHWAY glyoxylate shunt
COMMENT
//
REACTION MAS
LEFT acetyl-CoA + glyoxylate + H2O
RIGHT   (S)-MALATE + COA
LEFT_COMPARTMENT GLYOXYSOME
RIGHT_COMPARTMENT GLYOXYSOME
ENZYME_LEVEL    0.0
PATHWAY glyoxylate shunt
COMMENT
//
REACTION PYRDC
LEFT pyruvate
RIGHT acetaldehyde + CO2
LEFT_COMPARTMENT CYTOSOL
RIGHT_COMPARTMENT CYTOSOL
ENZYME_LEVEL    0.0
PATHWAY fermentation
COMMENT
//
REACTION ALDD2y
LEFT acetaldehyde + NADP+ + H2O
RIGHT acetate + NADPH
LEFT_COMPARTMENT CYTOSOL
RIGHT_COMPARTMENT CYTOSOL
ENZYME_LEVEL    0.0
PATHWAY fermentation
COMMENT
```

```
//
REACTION ALCD2X_copy1
LEFT acetaldehyde + NADH
RIGHT ethanol + NAD+
LEFT_COMPARTMENT CYTOSOL
RIGHT_COMPARTMENT CYTOSOL
ENZYME_LEVEL    0.0
PATHWAY fermentation
COMMENT
//
REACTION LactateDehydrogenase
LEFT pyruvate + NADH
RIGHT lactate + NAD+
LEFT_COMPARTMENT CYTOSOL
RIGHT_COMPARTMENT CYTOSOL
ENZYME_LEVEL    0.0
PATHWAY fermentation
COMMENT
//
```

Boltzmann Input File:

In [40]:
```
cat neurospora_pentose_phos.glycolysis.tca_reg.in
```

```
RXN_FILE neurospora_pentose_phos.glycolysis.tca.2.dat
INIT_FILE neurospora_pentose_phos.glycolysis.tca.2_reg.rstrt
INIT_FILE yeast_centralMetab_concs2.in
INIT_FILE neurospora_pentose_phos.glycolysis.tca_concs.in
INIT_FILE neurospora_pentose_phos.glycolysis.tca.2_reg.rstrt
LOG_FILE neurospora_pentose_phos.glycolysis.tca.2_reg.log
OUT_FILE neurospora_pentose_phos.glycolysis.tca.2_reg.out
USE_DEQ 1
NO_ROUND_FROM_DEQ 1
ODE_T_FINAL 100000
DELTA_CONCS_CHOICE 13
DELTA_CONCS_CHOICE 8
DERIV_THRESH  5.0e-17
ODE_RXN_VIEW_FREQ 1
WARMUP_STEPS 00000000
RECORD_STEPS 000000
TEMP_KELVIN 298.15
PH 7.0
IONIC_STRENGTH 0.15
PRINT_OUTPUT 2
CONCS_OR_COUNTS 3
RXN_VIEW_FREQ 100
COUNT_VIEW_FREQ 100
LKLHD_VIEW_FREQ 100
USE_BULK_WATER 1
USE_REGULATION 1
USE_ENZYME_LEVELS 1
USE_PSEUDOISOMERS 1
USE_DGZERO 1
USE_ACTIVITIES 1
NUM_METABOLIC_GROUPS 1
```

Models and Simulations

Model Without Regulation

Run Deterministic Simulation

Uncomment the lines below to run Boltzmann from the Jupyter notebook. We prefer not to run from the notebook because it may take several runs before the system converges.

In [41]:
```
#args = ("boltzmann", "neurospora_pentose_phos.glycolysis.tca_no_reg.i
n")
#popen = subprocess.Popen(args, stdout=subprocess.PIPE)
#popen.wait()
#output = popen.stdout.read()
#print(output)
```

Derivatives from ODE thermodynamic optimization simulation

For the maximum entropy optimization without any regulation, multiple rounds of optimization were used to obtain sufficiently converged derivatives. The maximum entropy distribution has a wide range of metabolite concentrations, from 10^{-22} for oxaloacetate to $10^{6}M$ for fructose-1,6-bisphosphate and $10^{9}M$ for acetyl-CoA. Despite these large differences, derivatives remain stable until the the derivatives for acetyl-CoA go below 0.05. At this point the solver becomes unstable because even a small time step results in a large change the derivative. However, the derivatives are sufficiently converged at this point that the loss function can be applied to the metabolite levels to infer reactions that should be regulated. To put this another way, it does not matter for the purpose of infering regulation if the acetyl-CoA concentraion is $1.0 \cdot 10^{9}$ or $6.0 \cdot 10^{9}$, both are well beyond the expected millimolar level.

In [42]:
```
# Read boltzmann stochastic reaction likelihoods
ode_derivatives = pd.read_table('neurospora_pentose_phos.glycolysis.tc
a.2_no_reg.ode_dconcs',header=0,index_col=0)
# Get the absolute value of the derivatives:
abs_derivatives = np.abs(ode_derivatives.iloc[-1,:])
display(abs_derivatives.sort_values())
#plt.figure();ode_derivatives.iloc[-5000:-1500,:].plot(legend=False,lo
gx=True,logy=False,figsize=(10, 10))
plt.figure();ode_derivatives.plot(legend=False,logx=False,logy=False,f
igsize=(10, 10))
plt.yscale('symlog')
#plt.legend(bbox_to_anchor=(1.35, 1.05),bbox_transform=plt.gcf().trans
Figure)
```

```
(S)-MALATE:CYTOSOL                          0.000000e+00
NADP+:CYTOSOL                               0.000000e+00
NADPH:CYTOSOL                               0.000000e+00
ORTHOPHOSPHATE:CYTOSOL                      0.000000e+00
SEDOHEPTULOSE_7-PHOSPHATE:CYTOSOL           0.000000e+00
(S)-MALATE:GLYOXYSOME                       0.000000e+00
ACETYL-COA:GLYOXYSOME                       0.000000e+00
COA:GLYOXYSOME                              0.000000e+00
GLYOXYLATE:GLYOXYSOME                       0.000000e+00
ISOCITRATE:GLYOXYSOME                       0.000000e+00
SUCCINATE:GLYOXYSOME                        0.000000e+00
ADP:MITOCHONDRIA                            0.000000e+00
ATP:MITOCHONDRIA                            0.000000e+00
CO2:MITOCHONDRIA                            0.000000e+00
COA:MITOCHONDRIA                            0.000000e+00
NAD+:MITOCHONDRIA                           0.000000e+00
```

```
NADH:MITOCHONDRIA                                 0.000000e+00
NADP+:MITOCHONDRIA                                0.000000e+00
NADPH:MITOCHONDRIA                                0.000000e+00
ORTHOPHOSPHATE:MITOCHONDRIA                       0.000000e+00
REDOX1:MITOCHONDRIA                               0.000000e+00
REDOX2:MITOCHONDRIA                               0.000000e+00
NADH:CYTOSOL                                      0.000000e+00
NAD+:CYTOSOL                                      0.000000e+00
OXALOACETATE:CYTOSOL                              0.000000e+00
CO2:CYTOSOL                                       0.000000e+00
LACTATE:CYTOSOL                                   0.000000e+00
D-ERYTHROSE-4-PHOSPHATE:CYTOSOL                   0.000000e+00
ADP:CYTOSOL                                       0.000000e+00
D-GLUCONO-1,5-LACTONE_6-PHOSPHATE:CYTOSOL         0.000000e+00
ATP:CYTOSOL                                       0.000000e+00
D-RIBOSE-5-PHOSPHATE:CYTOSOL                      0.000000e+00
ACETATE:CYTOSOL                                   0.000000e+00
ACETALDEHYDE:CYTOSOL                              0.000000e+00
6-PHOSPHO-D-GLUCONATE:CYTOSOL                     0.000000e+00
D-XYLULOSE:CYTOSOL                                0.000000e+00
D-XYLULOSE-5-PHOSPHATE:CYTOSOL                    0.000000e+00
ETHANOL:CYTOSOL                                   0.000000e+00
D-RIBULOSE-5-PHOSPHATE:CYTOSOL                    0.000000e+00
BETA-D-GLUCOSE:CYTOSOL                            0.000000e+00
ISOCITRATE:MITOCHONDRIA                           8.881784e-16
(S)-MALATE:MITOCHONDRIA                           8.881784e-16
2-OXOGLUTARATE:MITOCHONDRIA                       8.881784e-16
FUMARATE:MITOCHONDRIA                             8.881784e-16
OXALOACETATE:MITOCHONDRIA                         2.664535e-15
SUCCINYL-COA:MITOCHONDRIA                         2.664535e-15
SUCCINATE:MITOCHONDRIA                            2.664535e-15
CITRATE:MITOCHONDRIA                              2.664535e-15
D-GLYCERALDEHYDE-3-PHOSPHATE:CYTOSOL              9.769963e-15
GLYCERONE_PHOSPHATE:CYTOSOL                       5.000445e-13
PHOSPHOENOLPYRUVATE:CYTOSOL                       5.385301e-10
2-PHOSPHO-D-GLYCERATE:CYTOSOL                     9.066543e-10
BETA-D-GLUCOSE-6-PHOSPHATE:CYTOSOL                9.005226e-09
D-FRUCTOSE_6-PHOSPHATE:CYTOSOL                    1.085331e-08
3-PHOSPHO-D-GLYCERATE:CYTOSOL                     6.297109e-08
3-PHOSPHO-D-GLYCEROYL_PHOSPHATE:CYTOSOL           2.462820e-07
PYRUVATE:CYTOSOL                                  5.712797e-07
PYRUVATE:MITOCHONDRIA                             8.997118e-07
D-FRUCTOSE_1,6-BISPHOSPHATE:CYTOSOL               5.837993e-04
ACETYL-COA:MITOCHONDRIA                           1.248513e+00
Name: 9999981.0, dtype: float64

<matplotlib.figure.Figure at 0x11cc30470>
```

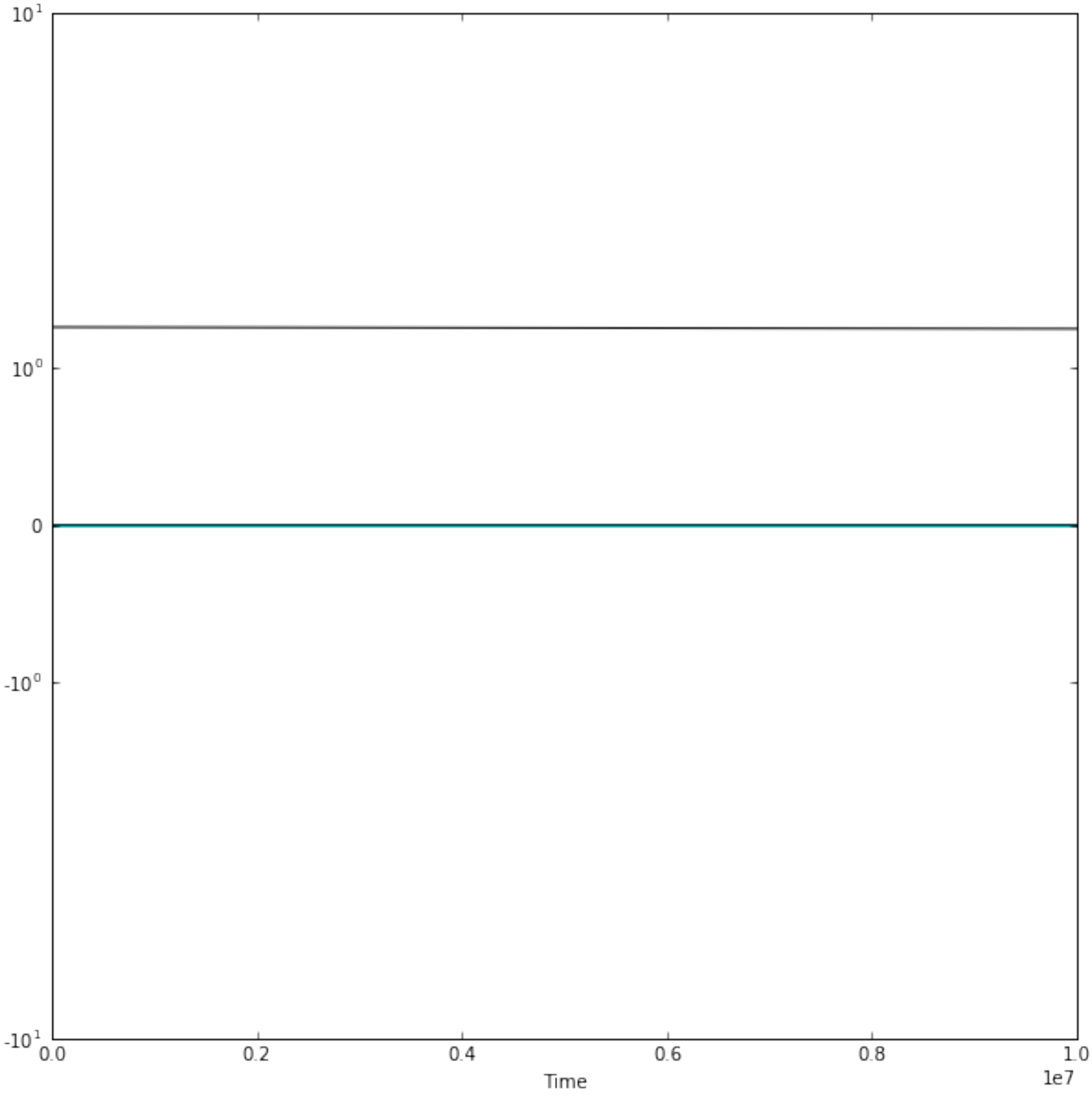

The values reported in the paper are those obtained just before the solver became unstable, at relative time 9.250000e+09.

In [43]:
```
#display(ode_derivatives.loc[9.250000e+09].sort_values())
```

Thermodynamic Odds of Reaction from Deterministic Simulation

The thermodynamic likelihoods are read in from the output files, where the likelihood is $e^{A_\alpha/RT} = K_\alpha Q_\alpha^{-1}$. A_α is the reaction affinity for reaction α, K_α is the equilibrium constant, and Q_α is the reaction quotient.

In [44]:

```
# Read boltzmann ODE reaction likelihoods
ode_likelihoods_timeseries = pd.read_table('neurospora_pentose_phos.gl
ycolysis.tca.2_no_reg.ode_lklhd',header=1, index_col = 0, quoting=2)

temp = [x for x in ode_likelihoods_timeseries.columns if 'f_' in x]
ode_fwd_likelihoods_timeseries = ode_likelihoods_timeseries[temp]
temp = [x for x in ode_likelihoods_timeseries.columns if 'r_' in x]
ode_rev_likelihoods_timeseries = ode_likelihoods_timeseries[temp]

ode_fwd_likelihoods_timeseries.columns = [x.split("f_")[-1] for x in o
de_fwd_likelihoods_timeseries.columns]
ode_rev_likelihoods_timeseries.columns = [x.split("r_")[-1] for x in o
de_rev_likelihoods_timeseries.columns]

plt.figure()
ode_fwd_likelihoods_timeseries.plot(legend=False,logx=False,figsize=(1
2, 12))
#plt.yscale('log')
#plt.yscale('symlog')
plt.legend(bbox_to_anchor=(0.02, 0.9),bbox_transform=plt.gcf().transFi
gure)
#display(ode_fwd_likelihoods_timeseries.loc[:9.250000e+09].mean())
display(ode_fwd_likelihoods_timeseries.mean())
```

```
ME1m                    0.000000
ME2m                    0.000000
CSm                     6.554156
ACONTm                  6.703329
ICDHxm                  6.582371
AKGDm                   6.582372
SUCOASm                 6.703335
SUCD1m                  6.703332
FUMm                    6.703327
MDHm                    6.554156
GAPD                    7.933921
PGK                     7.933929
TPI                     4.145176
MDH                     0.000000
PEP_Carboxylase         0.000000
PPCK                    0.000000
FBA                     4.145176
FBP                     0.000000
TKT2                    0.000000
RPE                     0.000000
Xylulokinase            0.000000
PYK_org                 0.000000
PYK                     7.933929
RPI                     0.000000
TKT1                    0.000000
TALA                    0.000000
PGM                     7.933930
ENO                     7.933930
GND                     0.000000
PGL                     0.000000
HEX1                    4.145744
PGI                     4.145744
G6PDH2r                 0.000000
PFK                     4.145744
PYRt2m                  7.933929
PDHm                    7.933923
ICL                     0.000000
MAS                     0.000000
PYRDC                   0.000000
ALDD2y                  0.000000
ALCD2X_copy1            0.000000
LactateDehydrogenase    0.000000
dtype: float64

<matplotlib.figure.Figure at 0x1050add68>
```

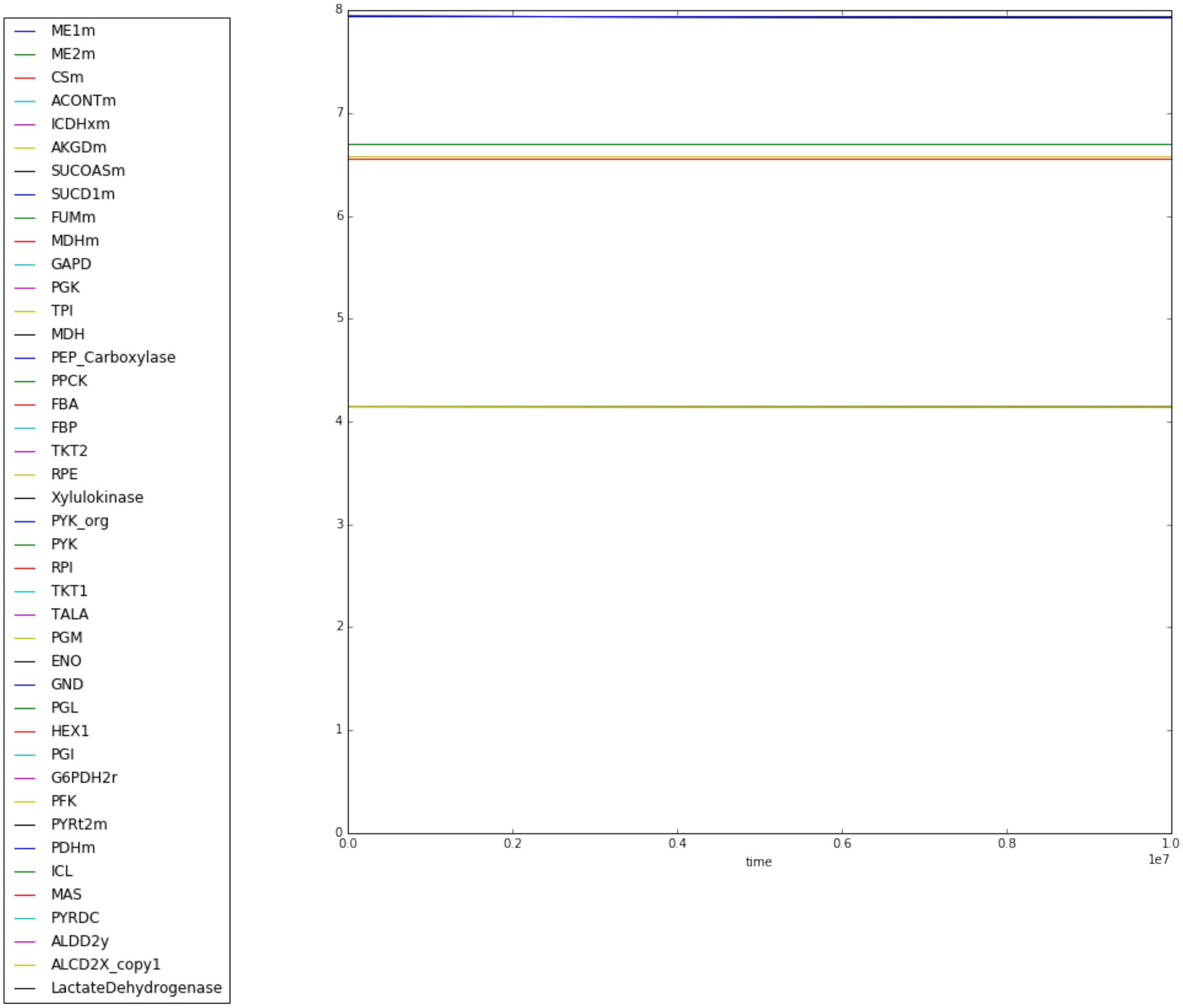

As mentioned above, when the the derivatives for acetyl-CoA go below 0.05, the solver becomes unstable because even a small time step results in a large change the derivative. However, the likelihoods are sufficiently converged at this point that good estimates of the steady state reaction fluxes can be obtained.

Calculate Net Odds

The net odds of a reaction are proportional to the reaction flux. To get the steady state values of the likelihoods, we'll use the average of the last 30 steps before the solver became unstable (relative time from 9.247000e+09 to 9.250000e+09).

In [45]:
```
#ode_likelihoods_steadystate = pd.DataFrame(data = ode_fwd_likelihoods
_timeseries.loc[9.247000e+09:9.250000e+09].mean(),
ode_likelihoods_steadystate = pd.DataFrame(data = ode_fwd_likelihoods_
timeseries.mean(),
                                           index = ode_fwd_likelihoods
_timeseries.columns,
                                          columns =['Forward'])
#ode_likelihoods_steadystate['Reverse'] = ode_rev_likelihoods_timeseri
es.loc[9.247000e+09:9.250000e+09].mean()
ode_likelihoods_steadystate['Reverse'] = ode_rev_likelihoods_timeserie
s.mean()
#ode_likelihoods_steadystate['Reverse'] = ode_likelihoods_steadystate[
'Forward'].rdiv(1)
ode_likelihoods_steadystate['For-Rev'] = ode_likelihoods_steadystate['
Forward'] - ode_likelihoods_steadystate['Reverse']
ode_likelihoods_steadystate['Rxn Probabilities'] = ode_likelihoods_ste
adystate['For-Rev']/np.sum(abs(ode_likelihoods_steadystate['For-Rev'])
)
display(ode_likelihoods_steadystate)
```

	Forward	Reverse	For-Rev	Rxn Probabilities
**ME1m**	0.000000	0.000000e+00	0.000000	0.000000
**ME2m**	0.000000	0.000000e+00	0.000000	0.000000
**CSm**	6.554156	5.290375e-14	6.554156	0.051767
**ACONTm**	6.703329	1.491725e-01	6.554156	0.051767
**ICDHxm**	6.582371	2.821428e-02	6.554157	0.051767
**AKGDm**	6.582372	2.821556e-02	6.554156	0.051767
**SUCOASm**	6.703335	1.491783e-01	6.554157	0.051767
**SUCD1m**	6.703332	1.491752e-01	6.554157	0.051767
**FUMm**	6.703327	1.491710e-01	6.554156	0.051767
**MDHm**	6.554156	5.290088e-14	6.554156	0.051767
**GAPD**	7.933921	1.260317e-01	7.807889	0.061669
**PGK**	7.933929	1.260406e-01	7.807889	0.061669
**TPI**	4.145176	2.412312e-01	3.903944	0.030834
**MDH**	0.000000	0.000000e+00	0.000000	0.000000
**PEP_Carboxylase**	0.000000	0.000000e+00	0.000000	0.000000
**PPCK**	0.000000	0.000000e+00	0.000000	0.000000
**FBA**	4.145176	2.412312e-01	3.903944	0.030834

**FBP**	0.000000	0.000000e+00	0.000000	0.000000
**TKT2**	0.000000	0.000000e+00	0.000000	0.000000
**RPE**	0.000000	0.000000e+00	0.000000	0.000000
**Xylulokinase**	0.000000	0.000000e+00	0.000000	0.000000
**PYK_org**	0.000000	0.000000e+00	0.000000	0.000000
**PYK**	7.933929	1.260406e-01	7.807889	0.061669
**RPI**	0.000000	0.000000e+00	0.000000	0.000000
**TKT1**	0.000000	0.000000e+00	0.000000	0.000000
**TALA**	0.000000	0.000000e+00	0.000000	0.000000
**PGM**	7.933930	1.260410e-01	7.807889	0.061669
**ENO**	7.933930	1.260410e-01	7.807889	0.061669
**GND**	0.000000	0.000000e+00	0.000000	0.000000
**PGL**	0.000000	0.000000e+00	0.000000	0.000000
**HEX1**	4.145744	2.412103e-01	3.904533	0.030839
**PGI**	4.145744	2.412112e-01	3.904533	0.030839
**G6PDH2r**	0.000000	0.000000e+00	0.000000	0.000000
**PFK**	4.145744	2.412105e-01	3.904533	0.030839
**PYRt2m**	7.933929	1.260410e-01	7.807888	0.061669
**PDHm**	7.933923	1.260362e-01	7.807887	0.061669
**ICL**	0.000000	0.000000e+00	0.000000	0.000000
**MAS**	0.000000	0.000000e+00	0.000000	0.000000
**PYRDC**	0.000000	0.000000e+00	0.000000	0.000000
**ALDD2y**	0.000000	0.000000e+00	0.000000	0.000000
**ALCD2X_copy1**	0.000000	0.000000e+00	0.000000	0.000000
**LactateDehydrogenase**	0.000000	0.000000e+00	0.000000	0.000000

Map Net Likelihoods onto Metabolic Pathways

In [46]:

```python
from escher import Builder

reaction_data = ode_likelihoods_steadystate['For-Rev'].to_dict()
#b = Builder(map_name="iMM904.PPP_TCA_Glycolysis.json",reaction_data=reaction_data)
b = Builder(map_name="iMM904.compact_Glycolysis_TCA_PPP.json",reaction_data=reaction_data)
#b.display_in_browser(menu='all')
b.display_in_notebook(menu='zoom')
```

Out[46]:

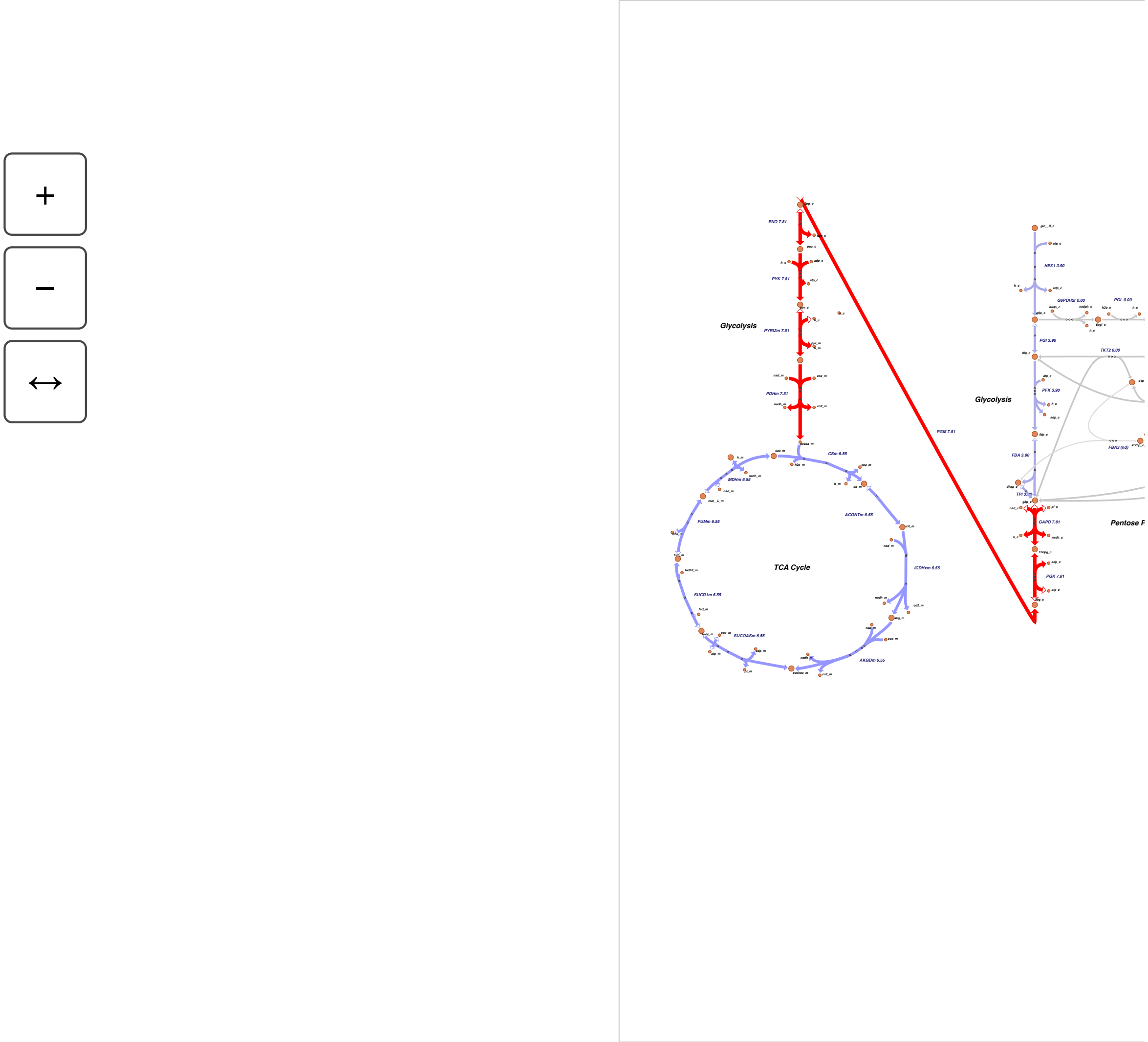

Find Active and Variable Metabolites

Some metabolites concentrations are parameters - those that form the boundary conditions such as cofactors, glucose and CO_2 - and others are free variables. Here we find the subset of metabolites that are free variables.

```
In [47]: # Find active reactions
# find chemicals that are in at least one reaction. Includes chemicals
that are fixed as well as
# variable ones:
S_active = pd.read_table('neurospora_pentose_phos.glycolysis.tca.2_no_
reg.amat',header=0, index_col = 0, quoting=2)
del S_active['forward reaction']

active_metabolites_idx = (S_active != 0).any(axis=0) #any searches dow
n the column
inactive_metabolites_idx = ~active_metabolites_idx

metabolites_status = pd.read_csv('neurospora_pentose_phos.glycolysis.t
ca.2_no_reg.rstrt',delimiter = '\t', index_col=0,skiprows = 2, header=
None,quoting=2,usecols=[0,2])
metabolites_status.rename(columns={2:'Value'},inplace=True)
metabolites_status.index = metabolites_status.index.str.strip()
metabolites_status['Variable?'] = metabolites_status == 'V'
metabolites_status.insert(1,'Active?',active_metabolites_idx.values)
del metabolites_status['Value']

metabolites_status['Variable & Active'] = metabolites_status['Variable
?']&metabolites_status['Active?']
variable_metabolite_idx = list(metabolites_status[metabolites_status['
Variable & Active']==True].index)
variable_metabolite_idx = list(map(str.strip, variable_metabolite_idx)
)
```

Time Derivatives of Metabolite Concentration: S · Net Odds = flux

This is a demonstration that the product of the stoichiometric matrix S and the net thermodynamic odds are close to zero, which is the steady state condition discussed in the manuscript. Here the stoichiometric matrix $S^{m,n}$ is defined as m reactions by n metabolites, which is the transpose of how it is used in the metabolic modeling literature.

In [48]:
```
S = pd.read_table('neurospora_pentose_phos.glycolysis.tca.2_no_reg.mat
',header=0, index_col = 0, quoting=2)
del S['forward reaction']

S = S.astype(np.float64)
# S.T.dot(ode_likelihoods_steadystate['For-Rev']) =  time derivative o
f concentrations
#                                                   0 at steady state
and maximum entropy.
# If boundary conditions prevent maximum entropy, then the flux !=0 fo
r boundary species.
derivatives2 = (S.T.dot(ode_likelihoods_steadystate['For-Rev'])).sort_
values()
display(derivatives2[variable_metabolite_idx].sort_values())
```

```
CITRATE:MITOCHONDRIA                         -4.990009e-07
SUCCINYL-COA:MITOCHONDRIA                    -2.600002e-07
ISOCITRATE:MITOCHONDRIA                      -2.200002e-07
SUCCINATE:MITOCHONDRIA                       -9.900094e-08
D-GLYCERALDEHYDE-3-PHOSPHATE:CYTOSOL         -2.457542e-08
BETA-D-GLUCOSE-6-PHOSPHATE:CYTOSOL           -1.108892e-08
2-PHOSPHO-D-GLYCERATE:CYTOSOL                -6.193806e-09
PHOSPHOENOLPYRUVATE:CYTOSOL                  -1.598404e-09
(S)-MALATE:MITOCHONDRIA                      -9.989307e-10
OXALOACETATE:MITOCHONDRIA                     0.000000e+00
GLYCERONE_PHOSPHATE:CYTOSOL                   1.098900e-09
D-FRUCTOSE_6-PHOSPHATE:CYTOSOL                5.944056e-08
3-PHOSPHO-D-GLYCERATE:CYTOSOL                 7.042957e-08
3-PHOSPHO-D-GLYCEROYL_PHOSPHATE:CYTOSOL       2.501498e-07
2-OXOGLUTARATE:MITOCHONDRIA                   2.800002e-07
PYRUVATE:CYTOSOL                              5.807193e-07
FUMARATE:MITOCHONDRIA                         7.990009e-07
PYRUVATE:MITOCHONDRIA                         8.989011e-07
D-FRUCTOSE_1,6-BISPHOSPHATE:CYTOSOL           5.887064e-04
ACETYL-COA:MITOCHONDRIA                       1.253731e+00
dtype: float64
```

Analyze Variable Metabolite Levels from Deterministic Simulation

In [49]:
```
# Read boltzmann stochastic reaction likelihoods
ode_metabolites = pd.read_table('neurospora_pentose_phos.glycolysis.tc
a.2_no_reg.ode_concs',header=0,index_col=0)

fig = plt.figure();
plot = ode_metabolites[variable_metabolite_idx].plot(legend=False,logy
=True,logx=True,figsize=(10, 10))
fig = plot.get_figure()
fig.savefig('metabolites_no_regulation.png')
plt.legend(bbox_to_anchor=(1.40, 0.90),bbox_transform=plt.gcf().transF
igure)
fig.savefig('metabolites_no_regulation_legend.png',bbox_inches='tight'
)
```

<matplotlib.figure.Figure at 0x105048e48>

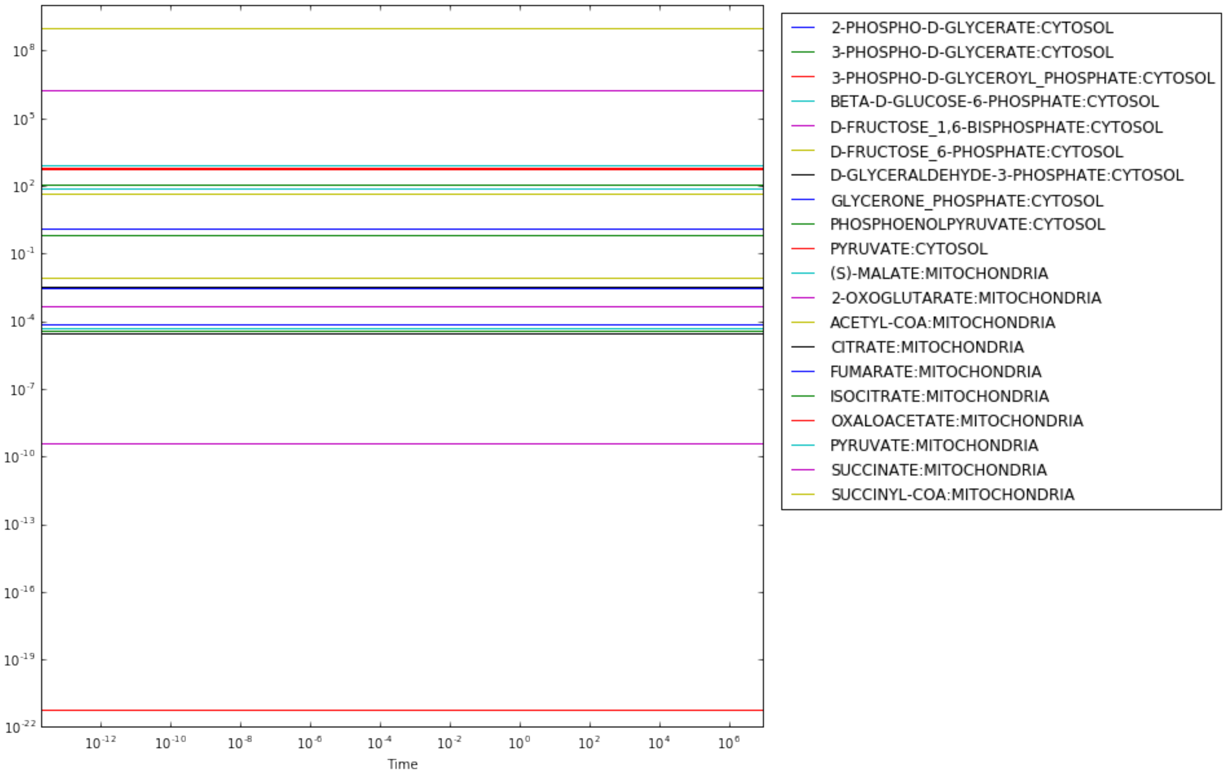

In [50]:
```
ode_metabolites_steadystate = \
  pd.DataFrame(data = ode_metabolites.iloc[-20:,:].mean(),columns=['OD
E'])
ode_metabolites_steadystate.sort_values(by=['ODE'])
```

Out[50]:

	ODE
**D-GLUCONO-1,5-LACTONE_6-PHOSPHATE:CYTOSOL**	0.000000e+00
**LACTATE:CYTOSOL**	0.000000e+00
**6-PHOSPHO-D-GLUCONATE:CYTOSOL**	0.000000e+00
**ACETALDEHYDE:CYTOSOL**	0.000000e+00

**ACETATE:CYTOSOL**	0.000000e+00
**ETHANOL:CYTOSOL**	0.000000e+00
**OXALOACETATE:MITOCHONDRIA**	5.723803e-22
**2-OXOGLUTARATE:MITOCHONDRIA**	3.787650e-10
**D-ERYTHROSE-4-PHOSPHATE:CYTOSOL**	5.149502e-08
**D-RIBULOSE-5-PHOSPHATE:CYTOSOL**	2.890365e-07
**D-RIBOSE-5-PHOSPHATE:CYTOSOL**	3.189369e-07
**GLYOXYLATE:GLYOXYSOME**	1.000000e-06
**NADP+:MITOCHONDRIA**	2.100000e-06
**NADP+:CYTOSOL**	2.100000e-06
**D-XYLULOSE-5-PHOSPHATE:CYTOSOL**	2.320598e-06
**D-GLYCERALDEHYDE-3-PHOSPHATE:CYTOSOL**	3.084260e-05
**ISOCITRATE:MITOCHONDRIA**	3.539343e-05
**(S)-MALATE:MITOCHONDRIA**	4.706638e-05
**FUMARATE:MITOCHONDRIA**	7.350155e-05
**NADH:MITOCHONDRIA**	8.300000e-05
**NADH:CYTOSOL**	8.300000e-05
**CO2:MITOCHONDRIA**	1.000000e-04
**CO2:CYTOSOL**	1.000000e-04
**NADPH:MITOCHONDRIA**	1.200000e-04
**NADPH:CYTOSOL**	1.200000e-04
**SEDOHEPTULOSE_7-PHOSPHATE:CYTOSOL**	3.035266e-04
**SUCCINATE:MITOCHONDRIA**	4.927172e-04
**ADP:CYTOSOL**	5.600000e-04
**ADP:MITOCHONDRIA**	5.600000e-04
**SUCCINATE:GLYOXYSOME**	5.700000e-04
**ISOCITRATE:GLYOXYSOME**	1.000000e-03
**OXALOACETATE:CYTOSOL**	1.000000e-03
**REDOX1:MITOCHONDRIA**	1.000000e-03
**REDOX2:MITOCHONDRIA**	1.000000e-03
**COA:MITOCHONDRIA**	1.400000e-03

**COA:GLYOXYSOME**	1.400000e-03
**(S)-MALATE:CYTOSOL**	1.700000e-03
**(S)-MALATE:GLYOXYSOME**	1.700000e-03
**BETA-D-GLUCOSE:CYTOSOL**	2.000000e-03
**NAD+:MITOCHONDRIA**	2.600000e-03
**NAD+:CYTOSOL**	2.600000e-03
**GLYCERONE_PHOSPHATE:CYTOSOL**	2.808746e-03
**ACETYL-COA:GLYOXYSOME**	2.863050e-03
**CITRATE:MITOCHONDRIA**	3.467543e-03
**D-XYLULOSE:CYTOSOL**	8.800000e-03
**SUCCINYL-COA:MITOCHONDRIA**	8.866397e-03
**ATP:CYTOSOL**	9.600000e-03
**ATP:MITOCHONDRIA**	9.600000e-03
**ORTHOPHOSPHATE:CYTOSOL**	2.000000e-02
**ORTHOPHOSPHATE:MITOCHONDRIA**	2.000000e-02
**PHOSPHOENOLPYRUVATE:CYTOSOL**	6.645813e-01
**2-PHOSPHO-D-GLYCERATE:CYTOSOL**	1.330016e+00
**D-FRUCTOSE_6-PHOSPHATE:CYTOSOL**	4.584099e+01
**BETA-D-GLUCOSE-6-PHOSPHATE:CYTOSOL**	7.607134e+01
**3-PHOSPHO-D-GLYCERATE:CYTOSOL**	1.138637e+02
**3-PHOSPHO-D-GLYCEROYL_PHOSPHATE:CYTOSOL**	5.803109e+02
**PYRUVATE:CYTOSOL**	6.084000e+02
**PYRUVATE:MITOCHONDRIA**	8.427092e+02
**D-FRUCTOSE_1,6-BISPHOSPHATE:CYTOSOL**	1.643838e+06
**ACETYL-COA:MITOCHONDRIA**	1.043645e+09

Infer Regulated Reactions

Regulated reactions are inferred using a loss function defined as the log ratio of the observed values $n_{i(\alpha)}$ to the predicted values $\tilde{n_{i(\alpha)}}$ of the $M(\alpha)$ reaction products of reaction α, [L{*\alpha }=\log \prod\limits*{i(\alpha)}^{M(\alpha)}{\frac{n*{i(\alpha)}}{\tilde{n}*{i(\alpha)}}}.]

In [51]:

```
ode_metabolites_steadystate['Expected'] = 1.0e-03

S = pd.read_table('neurospora_pentose_phos.glycolysis.tca.2_no_reg.mat
',header=0, index_col = 0, quoting=2)
P = (S>0)
del P['forward reaction']

P = P.astype(np.float64)
#display(R)
product_concentrations = P.multiply(ode_metabolites_steadystate['ODE']
,axis=1)
product_concentrations[product_concentrations == 0] = 1
product_concentrations_rxns = pd.DataFrame(data = product_concentratio
ns.T.product(), columns=['ODE'])
expect_product_concentrations = P.multiply(ode_metabolites_steadystate
['Expected'],axis=1)
expect_product_concentrations[expect_product_concentrations == 0] = 1
product_concentrations_rxns['Expected'] = (expect_product_concentratio
ns.T).product()

s_regulation = '$L_{reg}$'
product_concentrations_rxns[s_regulation] = \
    np.log(product_concentrations_rxns['ODE']/product_concentrations_r
xns['Expected'])

idx = (ode_likelihoods_steadystate['Reverse'] != 0) & (ode_likelihoods
_steadystate['Forward'] != 0) & \
            (product_concentrations_rxns[s_regulation]>1)
#display(product_concentrations_rxns[product_concentrations_rxns[s_reg
ulation]>1])
display(product_concentrations_rxns[idx].sort_values(by=s_regulation))
product_concentrations_rxns[idx].to_csv(path_or_buf='loss_no_regulatio
n.txt',sep='\t')
```

	ODE	Expected	L_{reg}
**reaction title**			
**CSm**	4.854560e-06	1.000000e-06	1.579919
**SUCOASm**	6.622119e-09	1.000000e-09	1.890415
**ENO**	6.645813e-01	1.000000e-03	6.499157
**PGM**	1.330016e+00	1.000000e-03	7.192947
**HEX1**	4.259995e-02	1.000000e-06	10.659608
**PGI**	4.584099e+01	1.000000e-03	10.732934
**GAPD**	4.816581e-02	1.000000e-06	10.782405
**PYRt2m**	8.427092e+02	1.000000e-03	13.644377
**PGK**	1.093091e+00	1.000000e-06	13.904520
**PYK**	5.840640e+00	1.000000e-06	15.580351
**PFK**	9.205491e+02	1.000000e-06	20.640481
**PDHm**	8.662250e+00	1.000000e-09	22.882240
