# Supplementary material for: Prediction of Metabolite Concentrations, Rate Constants and Post-Translational Regulation Using Maximum Entropy-Based Simulations with Application to Central Metabolism of Neurospora crassa
Source: Processes (Basel). Author manuscript; Available in PMC 2021 Apr 5. (PMC8020867; doi:10.3390/pr6060063)
Supplement: NotebookS2 [file NIHMS1061468-supplement-NotebookS2.pdf]

Table of Contents

1 Boltzmann
1.1 Pentose phosphate + Glycolysis + TCA cycle
1.2 Regulation
1.3 Turning on Regulation and Setting Enzyme Levels
2 Models and Simulations
2.1 Model With Regulation
2.1.1 Run Deterministic Simulation
2.1.2 Derivatives from ODE thermodynamic optimization simulation
2.1.3 Find Active and Variable Metabolites
2.1.4 Activity-scaled Odds of Reaction from Deterministic Simulation
2.1.5 Analyze Metabolite Levels from Deterministic Simulation
2.2 Infer Regulated Reactions
2.3 Calculate Rate Constants
2.3.1 Equalize reaction probabilities
2.3.2 Calculate the Unregulated Thermodynamic likelihood of the reactions
2.3.3 Calculate Rate Constants/Parameters
2.3.4 Cutoff for time derivative of concentrations

In [108]:
```
import numpy as np
import pandas as pd
import matplotlib.pyplot as plt
import matplotlib
import subprocess
import re
import os
from IPython.core.display import display
pd.set_option('display.max_columns', 7,'display.max_rows', None)
%matplotlib inline
N_avogadro = 6.022140857e+23
VolCell = 1.0e-15
Concentration2Count = N_avogadro * VolCell
concentration_increment = 1/(N_avogadro*VolCell)
```

Change directories to where the simulation data is:

In [109]:
```
# Uncomment to change to your directory, if needed:
cwd = os.getcwd()
if 'simulation_data' not in cwd:
  mydir = cwd+'/simulation_data'
  os.chdir(mydir)
```

Boltzmann

A beta version of the simulation code is available on GitHub at https://github.com/PNNL-CompBio/Boltzmann (https://github.com/PNNL-CompBio/Boltzmann).

Pentose phosphate + Glycolysis + TCA cycle

Reactions:

In [110]:
```
cat neurospora_pentose_phos.glycolysis.tca.2.dat
```

```
REACTION ME1m
LEFT    (S)-MALATE + NAD+
RIGHT   pyruvate + NADH + CO2
LEFT_COMPARTMENT MITOCHONDRIA
RIGHT_COMPARTMENT MITOCHONDRIA
ENZYME_LEVEL    0.0
//
REACTION ME2m
LEFT    (S)-MALATE + NADP+
RIGHT   PYRUVATE + NADPH + CO2
LEFT_COMPARTMENT MITOCHONDRIA
RIGHT_COMPARTMENT MITOCHONDRIA
ENZYME_LEVEL    0.0
//
REACTION CSm
LEFT    OXALOACETATE + ACETYL-COA + H2O
RIGHT   CITRATE + COA
LEFT_COMPARTMENT MITOCHONDRIA
RIGHT_COMPARTMENT MITOCHONDRIA
COMMENT PH = 7.0, IONIC STRENGTH = 0.15 M
//
REACTION ACONTm
LEFT    CITRATE
RIGHT   ISOCITRATE
LEFT_COMPARTMENT MITOCHONDRIA
RIGHT_COMPARTMENT MITOCHONDRIA
COMMENT  PH = 7.0, IONIC STRENGTH = 0.15 M
COMMENT DOn't split into two reactions if max entropy is being used
- only one enzyme needs to be epressed
//
REACTION ICDHxm
LEFT    ISOCITRATE + NAD+
RIGHT   2-OXOGLUTARATE + NADH + CO2
LEFT_COMPARTMENT MITOCHONDRIA
RIGHT_COMPARTMENT MITOCHONDRIA
COMMENT PH = 7.0, IONIC STRENGTH = 0.15 M
//
REACTION AKGDm
```

```
LEFT    2-OXOGLUTARATE + COA + NAD+
RIGHT   SUCCINYL-COA + CO2 + NADH
LEFT_COMPARTMENT MITOCHONDRIA
RIGHT_COMPARTMENT MITOCHONDRIA
COMMENT PH = 7.0, IONIC STRENGTH = 0.15 M
//
REACTION SUCOASm
LEFT    SUCCINYL-COA + ADP + Orthophosphate
RIGHT   SUCCINATE + ATP + COA
LEFT_COMPARTMENT MITOCHONDRIA
RIGHT_COMPARTMENT MITOCHONDRIA
COMMENT PH = 7.0, IONIC STRENGTH = 0.15 M
//
REACTION SUCD1m
LEFT    SUCCINATE + redox1
RIGHT   FUMARATE + redox2
LEFT_COMPARTMENT MITOCHONDRIA
RIGHT_COMPARTMENT MITOCHONDRIA
COMMENT PH = 7.0, IONIC STRENGTH = 0.15 M
//
REACTION FUMm
LEFT    FUMARATE + H2O
RIGHT   (S)-MALATE
LEFT_COMPARTMENT MITOCHONDRIA
RIGHT_COMPARTMENT MITOCHONDRIA
COMMENT PH = 7.0, IONIC STRENGTH = 0.15 M
//
REACTION  MDHm
LEFT    (S)-MALATE + NAD+
RIGHT   OXALOACETATE + NADH
LEFT_COMPARTMENT MITOCHONDRIA
RIGHT_COMPARTMENT MITOCHONDRIA
COMMENT PH = 7.0, IONIC STRENGTH = 0.15 M
//
REACTION GAPD
LEFT    D-GLYCERALDEHYDE-3-PHOSPHATE + ORTHOPHOSPHATE + NAD+
RIGHT   3-Phospho-D-glyceroyl_phosphate + NADH
ENZYME_LEVEL    1.0
LEFT_COMPARTMENT CYTOSOL
RIGHT_COMPARTMENT CYTOSOL
PATHWAY GLUCONEOGENESIS, GLYCOLYSIS
COMMENTS  From Dennis
//
REACTION PGK
LEFT    3-Phospho-D-glyceroyl_phosphate + ADP
RIGHT   3-PHOSPHO-D-GLYCERATE + ATP
LEFT_COMPARTMENT CYTOSOL
RIGHT_COMPARTMENT CYTOSOL
ENZYME_LEVEL    1.0
PATHWAY GLUCONEOGENESIS, GLYCOLYSIS, CALVIN CYCLE
COMMENTS  From Dennis
//
REACTION TPI
```

```
LEFT    Glycerone_phosphate
RIGHT   D-GLYCERALDEHYDE-3-PHOSPHATE
LEFT_COMPARTMENT CYTOSOL
RIGHT_COMPARTMENT CYTOSOL
ENZYME_LEVEL    1.0
COMMENT From Equilibrator
PATHWAY CALVIN CYCLE, GLYCOLYSIS
//
REACTION  MDH
LEFT    (S)-MALATE + NAD+
RIGHT   OXALOACETATE + NADH
LEFT_COMPARTMENT CYTOSOL
RIGHT_COMPARTMENT CYTOSOL
ENZYME_LEVEL    0.0
COMMENT PH = 7.0, IONIC STRENGTH = 0.15 M
COMMENT Cytosolic
//
REACTION PEP_Carboxylase
LEFT    oxaloacetate + orthophosphate
RIGHT   phosphoenolpyruvate + CO2 + H2O
LEFT_COMPARTMENT CYTOSOL
RIGHT_COMPARTMENT CYTOSOL
ENZYME_LEVEL    0.0
PATHWAY GLUCONEOGENSESIS
COMMENTS
//
REACTION PPCK
LEFT    oxaloacetate + ATP
RIGHT   phosphoenolpyruvate + ADP + CO2
LEFT_COMPARTMENT CYTOSOL
RIGHT_COMPARTMENT CYTOSOL
ENZYME_LEVEL    0.0
PATHWAY GLUCONEOGENSESIS
COMMENTS
//
REACTION FBA
LEFT    D-FRUCTOSE_1,6-BISPHOSPHATE
RIGHT   Glycerone_phosphate + D-GLYCERALDEHYDE-3-PHOSPHATE
LEFT_COMPARTMENT CYTOSOL
RIGHT_COMPARTMENT CYTOSOL
ENZYME_LEVEL    1.0
PATHWAY GLUCONEOGENSESIS, GLYCOLYSIS, CALVIN CYCLE
COMMENTS  From Dennis
//
REACTION FBP
LEFT    D-FRUCTOSE_6-PHOSPHATE + Orthophosphate
RIGHT   H2O + D-FRUCTOSE_1,6-BISPHOSPHATE
LEFT_COMPARTMENT CYTOSOL
RIGHT_COMPARTMENT CYTOSOL
ENZYME_LEVEL    0.0
PATHWAY GLUCONEOGENESIS, CALVIN CYCLE
COMMENTS  From Dennis
//
```

```
REACTION TKT2
LEFT    D-FRUCTOSE_6-PHOSPHATE + D-GLYCERALDEHYDE-3-PHOSPHATE
RIGHT   D-ERYTHROSE-4-PHOSPHATE + D-XYLULOSE-5-PHOSPHATE
LEFT_COMPARTMENT CYTOSOL
RIGHT_COMPARTMENT CYTOSOL
ENZYME_LEVEL    0.0
PATHWAY CALVIN CYCLE, PENTOSE PHOSPHATE PATHWAY, RUBISCO SHUNT
COMMENT   From Equilibrator pH 7.5, IS 0.15
//
REACTION RPE
LEFT    D-XYLULOSE-5-PHOSPHATE
RIGHT   D-RIBULOSE-5-PHOSPHATE
LEFT_COMPARTMENT CYTOSOL
RIGHT_COMPARTMENT CYTOSOL
ENZYME_LEVEL    0.0
PATHWAY CALVIN CYCLE, PENTOSE PHOSPHATE PATHWAY, RUBISCO SHUNT
COMMENT  From Dennis
//
REACTION Xylulokinase
LEFT    D-XYLULOSE + ATP
RIGHT   D-XYLULOSE-5-PHOSPHATE + ADP
LEFT_COMPARTMENT CYTOSOL
RIGHT_COMPARTMENT CYTOSOL
ENZYME_LEVEL    0.0
PATHWAY CALVIN CYCLE, PENTOSE PHOSPHATE PATHWAY, RUBISCO SHUNT
COMMENT
//
REACTION PYK_org
LEFT    ADP + PHOSPHOENOLPYRUVATE
RIGHT   PYRUVATE + ATP
LEFT_COMPARTMENT CYTOSOL
RIGHT_COMPARTMENT CYTOSOL
ENZYME_LEVEL    0.0
PATHWAY GLYCOLYSIS, PYRUVATE METABOLISM, RUBISCO SHUNT
COMMENT  From Dennis
//
REACTION PYK
LEFT    ADP + PHOSPHOENOLPYRUVATE
RIGHT   PYRUVATE + ATP
LEFT_COMPARTMENT CYTOSOL
RIGHT_COMPARTMENT CYTOSOL
ENZYME_LEVEL    1.0
PATHWAY GLYCOLYSIS, PYRUVATE METABOLISM, RUBISCO SHUNT
COMMENT  From Dennis
//
REACTION RPI
LEFT    D-RIBOSE-5-PHOSPHATE
RIGHT   D-RIBULOSE-5-PHOSPHATE
LEFT_COMPARTMENT CYTOSOL
RIGHT_COMPARTMENT CYTOSOL
ENZYME_LEVEL    0.0
PATHWAY CALVIN CYCLE, PENTOSE PHOSPHATE PATHWAY, RUBISCO SHUNT
COMMENT  From Dennis
```

```
//
REACTION TKT1
LEFT    SEDOHEPTULOSE_7-PHOSPHATE + D-GLYCERALDEHYDE-3-PHOSPHATE
RIGHT   D-RIBOSE-5-PHOSPHATE + D-XYLULOSE-5-PHOSPHATE
LEFT_COMPARTMENT CYTOSOL
RIGHT_COMPARTMENT CYTOSOL
ENZYME_LEVEL    0.0
PATHWAY CALVIN CYCLE, PENTOSE PHOSPHATE PATHWAY, RUBISCO SHUNT
COMMENT  From Equilibrator pH 7.5, IS 0.15
//
REACTION TALA
LEFT    D-GLYCERALDEHYDE-3-PHOSPHATE + SEDOHEPTULOSE_7-PHOSPHATE
RIGHT   D-FRUCTOSE_6-PHOSPHATE + D-ERYTHROSE-4-PHOSPHATE
LEFT_COMPARTMENT CYTOSOL
RIGHT_COMPARTMENT CYTOSOL
ENZYME_LEVEL    0.0
PATHWAY  RUBISCO SHUNT, PENTOSE PHOSPHATE PATHWAY
COMMENT  From Equilibrator pH 7.5 IS 0.15
//
REACTION PGM
LEFT    3-PHOSPHO-D-GLYCERATE
RIGHT   2-PHOSPHO-D-GLYCERATE
LEFT_COMPARTMENT CYTOSOL
RIGHT_COMPARTMENT CYTOSOL
ENZYME_LEVEL    1.0
PATHWAY  GLUCONEOGENESIS, RUBISCO SHUNT, GLYCOLYSIS I
COMMENTS  From Dennis
//
REACTION  ENO
LEFT    2-PHOSPHO-D-GLYCERATE
RIGHT   PHOSPHOENOLPYRUVATE + H2O
LEFT_COMPARTMENT CYTOSOL
RIGHT_COMPARTMENT CYTOSOL
ENZYME_LEVEL    1.0
PATHWAY  GLUCONEOGENESIS, RUBISCO SHUNT, GLYCOLYSIS I
COMMENTS  From Dennis
//
# Pentose Phosphate reactions (oxidative branch):
REACTION GND
LEFT  NADPH + D-RIBULOSE-5-PHOSPHATE + CO2
RIGHT NADP+ + 6-PHOSPHO-D-GLUCONATE
LEFT_COMPARTMENT CYTOSOL
RIGHT_COMPARTMENT CYTOSOL
PATHWAY PENTOSE PHOSPHATE PATHWAY
ENZYME_LEVEL    0.0
//
REACTION  PGL
LEFT  6-PHOSPHO-D-GLUCONATE
RIGHT D-Glucono-1,5-lactone_6-phosphate + H2O
LEFT_COMPARTMENT CYTOSOL
RIGHT_COMPARTMENT CYTOSOL
COMMENT From Dennis
PATHWAY PENTOSE PHOSPHATE PATHWAY
```

```
ENZYME_LEVEL    0.0
//
REACTION HEX1
LEFT    BETA-D-GLUCOSE + ATP
RIGHT   BETA-D-GLUCOSE-6-PHOSPHATE + ADP
LEFT_COMPARTMENT CYTOSOL
RIGHT_COMPARTMENT CYTOSOL
NREGULATION ATP 2.80e-03 10
ENZYME_LEVEL    1.0
PATHWAY GLUCONEOGENESIS, (GLYCOLYSIS-YEAST), GLYCOLYSIS
COMMENTS  From Dennis
//
REACTION PGI
LEFT    BETA-D-GLUCOSE-6-PHOSPHATE
RIGHT   D-FRUCTOSE_6-PHOSPHATE
LEFT_COMPARTMENT CYTOSOL
RIGHT_COMPARTMENT CYTOSOL
ENZYME_LEVEL    1.0
PATHWAY GLUCONEOGENESIS, (GLYCOLYSIS-YEAST), GLYCOLYSIS
COMMENTS  From Dennis
//
# Gluconeogenesis reactions :
REACTION  G6PDH2r
LEFT    BETA-D-GLUCOSE-6-PHOSPHATE + NADP+
RIGHT   D-Glucono-1,5-lactone_6-phosphate + NADPH
LEFT_COMPARTMENT CYTOSOL
RIGHT_COMPARTMENT CYTOSOL
ENZYME_LEVEL    0.0
COMMENT  From Dennis
PATHWAY GLUCONEOGENESIS
//
# Glycolysis reactions :
REACTION  PFK
LEFT    D-FRUCTOSE_6-PHOSPHATE + ATP
RIGHT   ADP + D-FRUCTOSE_1,6-BISPHOSPHATE
LEFT_COMPARTMENT CYTOSOL
RIGHT_COMPARTMENT CYTOSOL
NREGULATION ATP 2.80e-03 10
ENZYME_LEVEL    1.0
PATHWAY GLYCOLYSIS
COMMNENT From Equilibrator
//
REACTION PYRt2m
LEFT    PYRUVATE
RIGHT   PYRUVATE
DGZERO  -5.94
COMMENT -5.94
DGZERO-UNITS    KJ/MOL
LEFT_COMPARTMENT CYTOSOL
RIGHT_COMPARTMENT MITOCHONDRIA
ENZYME_LEVEL    1.0
COMMENT PYRUVATE TRANSPORT INTO MITOCHONDRIA - Assuming free energy
difference = \Delta -RT \Delta pH = -RT log 10
```

```
//
# Acetyl CoA synthesis
REACTION PDHm
LEFT    COA + NAD+ + PYRUVATE
RIGHT   ACETYL-COA + CO2 + NADH
LEFT_COMPARTMENT  MITOCHONDRIA
RIGHT_COMPARTMENT MITOCHONDRIA
ENZYME_LEVEL    1.0
NREGULATION ACETYL-COA 1.00e-03 20
PATHWAY ACETYL COA BIOSYNTHESIS
COMMENT PYRUVATE DEHYDROGENASE SUMMARY REACTION
//
REACTION ICL
LEFT isocitrate
RIGHT   glyoxylate + succinate
LEFT_COMPARTMENT GLYOXYSOME
RIGHT_COMPARTMENT GLYOXYSOME
ENZYME_LEVEL    0.0
PATHWAY glyoxylate shunt
COMMENT
//
REACTION MAS
LEFT acetyl-CoA + glyoxylate + H2O
RIGHT   (S)-MALATE + COA
LEFT_COMPARTMENT GLYOXYSOME
RIGHT_COMPARTMENT GLYOXYSOME
ENZYME_LEVEL    0.0
PATHWAY glyoxylate shunt
COMMENT
//
REACTION PYRDC
LEFT pyruvate
RIGHT acetaldehyde + CO2
LEFT_COMPARTMENT CYTOSOL
RIGHT_COMPARTMENT CYTOSOL
ENZYME_LEVEL    0.0
PATHWAY fermentation
COMMENT
//
REACTION ALDD2y
LEFT acetaldehyde + NADP+ + H2O
RIGHT acetate + NADPH
LEFT_COMPARTMENT CYTOSOL
RIGHT_COMPARTMENT CYTOSOL
ENZYME_LEVEL    0.0
PATHWAY fermentation
COMMENT
//
REACTION ALCD2X_copy1
LEFT acetaldehyde + NADH
RIGHT ethanol + NAD+
LEFT_COMPARTMENT CYTOSOL
RIGHT_COMPARTMENT CYTOSOL
```

```
ENZYME_LEVEL    0.0
PATHWAY fermentation
COMMENT
//
REACTION LactateDehydrogenase
LEFT pyruvate + NADH
RIGHT lactate + NAD+
LEFT_COMPARTMENT CYTOSOL
RIGHT_COMPARTMENT CYTOSOL
ENZYME_LEVEL    0.0
PATHWAY fermentation
COMMENT
//
```

Boltzmann Input File:

In [111]:
```
cat neurospora_pentose_phos.glycolysis.tca_reg.in
```

```
RXN_FILE neurospora_pentose_phos.glycolysis.tca.2.dat
INIT_FILE neurospora_pentose_phos.glycolysis.tca.2_reg.rstrt
INIT_FILE yeast_centralMetab_concs2.in
INIT_FILE neurospora_pentose_phos.glycolysis.tca_concs.in
INIT_FILE neurospora_pentose_phos.glycolysis.tca.2_reg.rstrt
LOG_FILE neurospora_pentose_phos.glycolysis.tca.2_reg.log
OUT_FILE neurospora_pentose_phos.glycolysis.tca.2_reg.out
USE_DEQ 1
NO_ROUND_FROM_DEQ 1
ODE_T_FINAL 100000
DELTA_CONCS_CHOICE 13
DELTA_CONCS_CHOICE 8
DERIV_THRESH  5.0e-17
ODE_RXN_VIEW_FREQ 1
WARMUP_STEPS 00000000
RECORD_STEPS 000000
TEMP_KELVIN 298.15
PH 7.0
IONIC_STRENGTH 0.15
PRINT_OUTPUT 2
CONCS_OR_COUNTS 3
RXN_VIEW_FREQ 100
COUNT_VIEW_FREQ 100
LKLHD_VIEW_FREQ 100
USE_BULK_WATER 1
USE_REGULATION 1
USE_ENZYME_LEVELS 1
USE_PSEUDOISOMERS 1
USE_DGZERO 1
USE_ACTIVITIES 1
NUM_METABOLIC_GROUPS 1
```

Regulation

Notice that in the PDH reaction there is a line:

NREGULATION ACETYL-COA 1.00e-03 20.0

The keyword NREGULATION is for negative regulation using a Hill Function with constant = 6.1e-04 and an exponent of 20.0

$$Activity = \frac{K^n}{K^n + X^n}$$

where K is the constant similar to the Michaelis constant, n is the exponent that controls the steepness of the Hill function and X is the concentration of the species that regulates the activity. The Hill function is used to update the activity of the enzyme as follows:

In [112]:
```
constant = 6.1e-04
exponent = 10
constant_pow_exponent = np.exp(exponent*np.log(constant))

concentration = np.arange(1.0e-4, 1.0e-3, 1.0e-5)
concentration_pow_exponent = np.exp(np.multiply(exponent,np.log(concen
tration)))

activity = np.divide(constant_pow_exponent,(constant_pow_exponent + co
ncentration_pow_exponent))
plt.plot(concentration,activity)
plt.show()
```

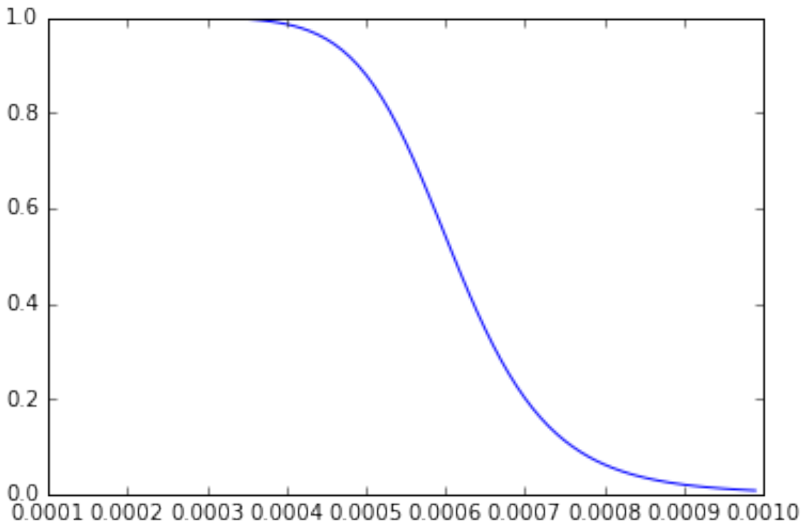

Likewise, positive regulation is done using the comparable Hill Function,

$$Activity = \frac{X^n}{K^n + X^n}$$

. Positive regulation is indicated in the Boltzmann .dat file with keyword PREGULATION.

In [113]:
```
constant = 6.1e-04
exponent = 10
constant_pow_exponent = np.exp(exponent*np.log(constant))

concentration = np.arange(1.0e-4, 1.0e-3, 1.0e-5)
concentration_pow_exponent = np.exp(np.multiply(exponent,np.log(concen
tration)))

activity = np.divide(concentration_pow_exponent,(constant_pow_exponent
+ concentration_pow_exponent))
plt.plot(concentration,activity)
```

Out[113]: [<matplotlib.lines.Line2D at 0x10fd54518>]

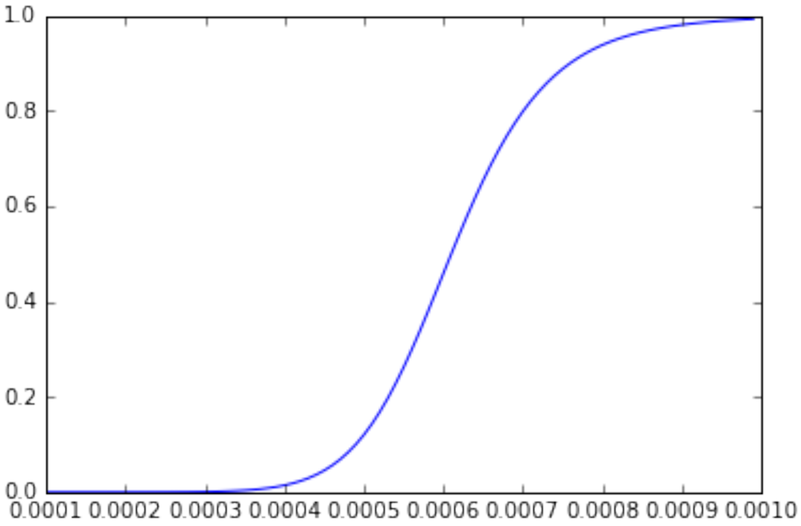

Turning on Regulation and Setting Enzyme Levels

To use regulation in the simulation, this flag must be set in the .in file:

USE_REGULATION 1

If USE_REGULATION is set to 0, then the regulation information in the .dat file will be ignored.

Reactions can be turned on (1.0) or off (0.0) by setting the ENZYME_LEVEL feature in the .dat file for a reaction:

USE_ENZYME_LEVELS 1.0

or

USE_ENZYME_LEVELS 0.0

In addition, USE_ACTIVITY 1" must be set in the .in file.

Models and Simulations

Model With Regulation

Run Deterministic Simulation

Uncomment the lines below to run from the notebook.

In [114]:
```
#args = ("boltzmann", "neurospora_pentose_phos.glycolysis.tca_reg.in")
#
#popen = subprocess.Popen(args, stdout=subprocess.PIPE)
#popen.wait()
#output = popen.stdout.read()
#print(output)
```

Derivatives from ODE thermodynamic optimization simulation

In [115]:
```
# Read boltzmann stochastic reaction likelihoods
ode_derivatives = pd.read_table('neurospora_pentose_phos.glycolysis.tc
a.2_reg.ode_dconcs',header=0,index_col=0)

abs_ode_derivatives = np.abs(ode_derivatives)
derivatives = np.abs(ode_derivatives.iloc[-1,:])
temp = derivatives.sort_values()
display(temp)
plt.figure();abs_ode_derivatives.plot(legend=False,logx=True,logy=Fals
e,figsize=(10, 10))
plt.yscale('symlog')
#plt.legend(bbox_to_anchor=(1.35, 1.05),bbox_transform=plt.gcf().trans
Figure)
```

```
(S)-MALATE:CYTOSOL                              0.000000e+00
NADPH:CYTOSOL                                   0.000000e+00
ORTHOPHOSPHATE:CYTOSOL                          0.000000e+00
SUCCINATE:MITOCHONDRIA                          0.000000e+00
SEDOHEPTULOSE_7-PHOSPHATE:CYTOSOL               0.000000e+00
(S)-MALATE:GLYOXYSOME                           0.000000e+00
ACETYL-COA:GLYOXYSOME                           0.000000e+00
COA:GLYOXYSOME                                  0.000000e+00
GLYOXYLATE:GLYOXYSOME                           0.000000e+00
ISOCITRATE:GLYOXYSOME                           0.000000e+00
SUCCINATE:GLYOXYSOME                            0.000000e+00
NADP+:CYTOSOL                                   0.000000e+00
ADP:MITOCHONDRIA                                0.000000e+00
CO2:MITOCHONDRIA                                0.000000e+00
```

```
COA:MITOCHONDRIA                                  0.000000e+00
NAD+:MITOCHONDRIA                                 0.000000e+00
NADH:MITOCHONDRIA                                 0.000000e+00
NADP+:MITOCHONDRIA                                0.000000e+00
NADPH:MITOCHONDRIA                                0.000000e+00
ORTHOPHOSPHATE:MITOCHONDRIA                       0.000000e+00
OXALOACETATE:MITOCHONDRIA                         0.000000e+00
REDOX1:MITOCHONDRIA                               0.000000e+00
REDOX2:MITOCHONDRIA                               0.000000e+00
ATP:MITOCHONDRIA                                  0.000000e+00
NADH:CYTOSOL                                      0.000000e+00
OXALOACETATE:CYTOSOL                              0.000000e+00
LACTATE:CYTOSOL                                   0.000000e+00
2-PHOSPHO-D-GLYCERATE:CYTOSOL                     0.000000e+00
6-PHOSPHO-D-GLUCONATE:CYTOSOL                     0.000000e+00
ACETALDEHYDE:CYTOSOL                              0.000000e+00
ACETATE:CYTOSOL                                   0.000000e+00
ADP:CYTOSOL                                       0.000000e+00
ATP:CYTOSOL                                       0.000000e+00
BETA-D-GLUCOSE:CYTOSOL                            0.000000e+00
CO2:CYTOSOL                                       0.000000e+00
NAD+:CYTOSOL                                      0.000000e+00
D-ERYTHROSE-4-PHOSPHATE:CYTOSOL                   0.000000e+00
D-RIBOSE-5-PHOSPHATE:CYTOSOL                      0.000000e+00
ETHANOL:CYTOSOL                                   0.000000e+00
D-RIBULOSE-5-PHOSPHATE:CYTOSOL                    0.000000e+00
D-XYLULOSE-5-PHOSPHATE:CYTOSOL                    0.000000e+00
D-GLUCONO-1,5-LACTONE_6-PHOSPHATE:CYTOSOL         0.000000e+00
D-XYLULOSE:CYTOSOL                                0.000000e+00
D-FRUCTOSE_6-PHOSPHATE:CYTOSOL                    2.220446e-16
D-GLYCERALDEHYDE-3-PHOSPHATE:CYTOSOL              2.220446e-16
3-PHOSPHO-D-GLYCERATE:CYTOSOL                     4.440892e-16
BETA-D-GLUCOSE-6-PHOSPHATE:CYTOSOL                4.440892e-16
PHOSPHOENOLPYRUVATE:CYTOSOL                       4.440892e-16
D-FRUCTOSE_1,6-BISPHOSPHATE:CYTOSOL               6.661338e-16
GLYCERONE_PHOSPHATE:CYTOSOL                       6.661338e-16
3-PHOSPHO-D-GLYCEROYL_PHOSPHATE:CYTOSOL           8.881784e-16
SUCCINYL-COA:MITOCHONDRIA                         8.881784e-16
2-OXOGLUTARATE:MITOCHONDRIA                       8.881784e-16
PYRUVATE:CYTOSOL                                  8.881784e-16
FUMARATE:MITOCHONDRIA                             1.332268e-15
CITRATE:MITOCHONDRIA                              1.332268e-15
(S)-MALATE:MITOCHONDRIA                           1.332268e-15
ISOCITRATE:MITOCHONDRIA                           1.332268e-15
ACETYL-COA:MITOCHONDRIA                           6.661338e-15
PYRUVATE:MITOCHONDRIA                             6.661338e-15
Name: 99999.64, dtype: float64
```

```
<matplotlib.figure.Figure at 0x10b6de0b8>
```

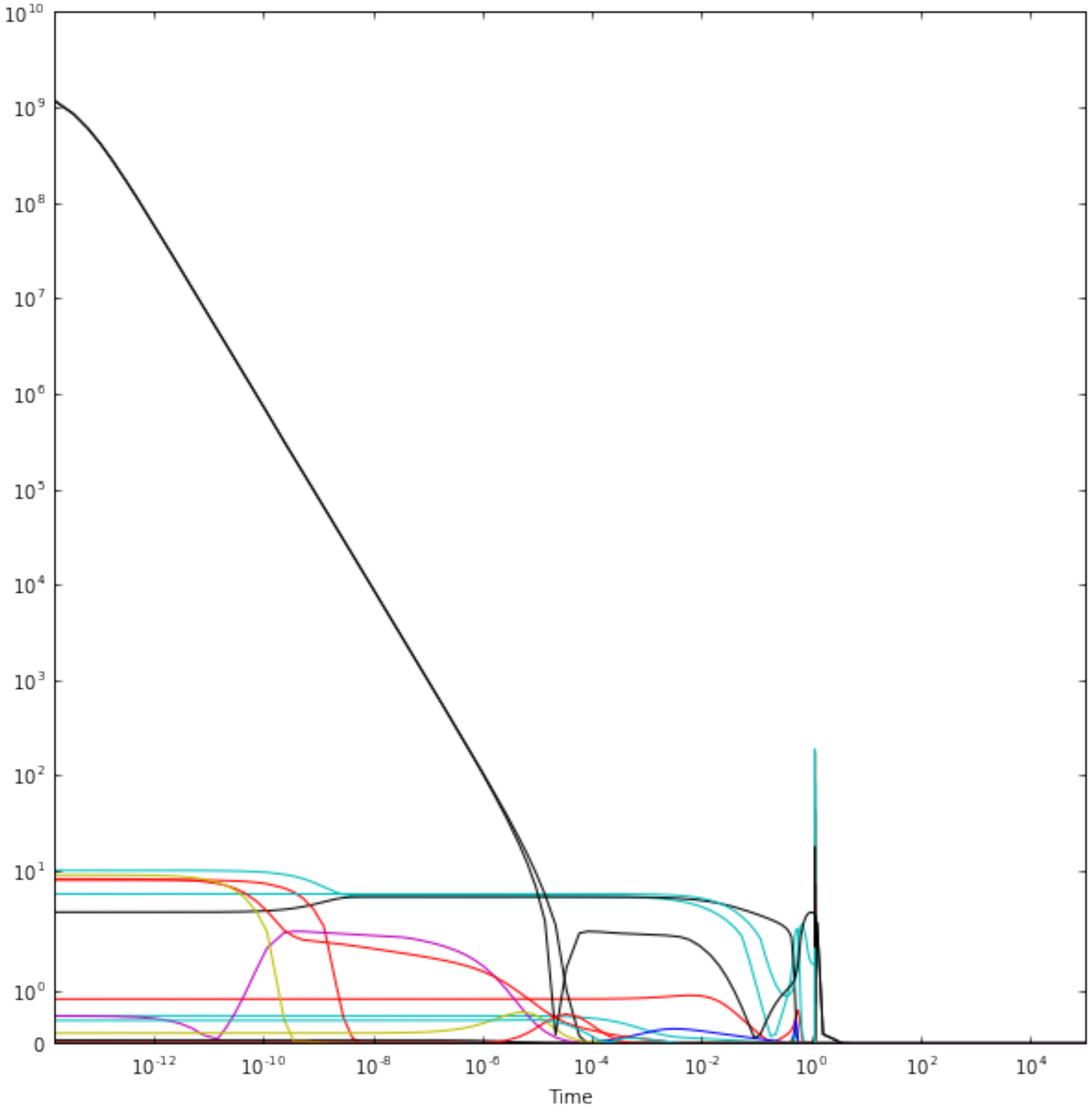

Find Active and Variable Metabolites

In [116]:

```
# Find active reactions
# find chemicals that are in at least one reaction. Includes chemicals that are fixed as well as
# variable ones:
S_active = pd.read_table('neurospora_pentose_phos.glycolysis.tca.2_reg.amat',header=0, index_col = 0, quoting=2)
del S_active['forward reaction']

active_metabolites_idx = (S_active != 0).any(axis=0) #any searches down the column
inactive_metabolites_idx = ~active_metabolites_idx

metabolites_status = pd.read_csv('neurospora_pentose_phos.glycolysis.tca.2_reg.rstrt',delimiter = '\t', index_col=0,skiprows = 2, header=None,quoting=2,usecols=[0,2])
metabolites_status.rename(columns={2:'Value'},inplace=True)
metabolites_status.index = metabolites_status.index.str.strip()
metabolites_status['Variable?'] = metabolites_status == 'V'
metabolites_status.insert(1,'Active?',active_metabolites_idx.values)
del metabolites_status['Value']

metabolites_status['Variable & Active'] = metabolites_status['Variable?']&metabolites_status['Active?']
variable_metabolite_idx = list(metabolites_status[metabolites_status['Variable & Active']==True].index)
variable_metabolite_idx = list(map(str.strip, variable_metabolite_idx))
```

Activity-scaled Odds of Reaction from Deterministic Simulation

The activity-scaled thermodynamic odds are read in from the output files, where the odds of a reaction α is $\lambda_\alpha \cdot e^{A_\alpha/RT} = \lambda_\alpha \cdot K_\alpha Q_\alpha^{-1}$. A_α is the reaction affinity for reaction α, λ_α is the activity, K_α is the equilibrium constant, and Q_α is the reaction quotient.

In [117]:
```
# Read boltzmann ODE reaction likelihoods
ode_likelihoods_timeseries = pd.read_table('neurospora_pentose_phos.gl
ycolysis.tca.2_reg.ode_lklhd',header=1, index_col = 0, quoting=2)

temp = [x for x in ode_likelihoods_timeseries.columns if 'f_' in x]
ode_fwd_likelihoods_timeseries = ode_likelihoods_timeseries[temp]
temp = [x for x in ode_likelihoods_timeseries.columns if 'r_' in x]
ode_rev_likelihoods_timeseries = ode_likelihoods_timeseries[temp]

ode_fwd_likelihoods_timeseries.columns = [x.split("f_")[-1] for x in o
de_fwd_likelihoods_timeseries.columns]
ode_rev_likelihoods_timeseries.columns = [x.split("r_")[-1] for x in o
de_rev_likelihoods_timeseries.columns]
#fwd_column = ode_likelihoods_timeseries.iloc[:,fwd_column_idx].column
s
#rev_column = ode_likelihoods_timeseries.iloc[:,rev_column_idx].column
s

plt.figure()
#ode_fwd_likelihoods_timeseries.plot(legend=True,logx=False,figsize=(1
2, 12))
ode_fwd_likelihoods_timeseries.iloc[-1000:].plot(legend=False,logx=Tru
e,figsize=(12, 12))
plt.yscale('symlog')
#plt.legend(bbox_to_anchor=(1.02, 0.9),bbox_transform=plt.gcf().transF
igure)
display(ode_fwd_likelihoods_timeseries.iloc[-30:,].mean())
```

```
ME1m                    0.000000
ME2m                    0.000000
CSm                     3.167760
ACONTm                  3.172948
ICDHxm                  2.943128
AKGDm                   2.943136
SUCOASm                 3.172974
SUCD1m                  3.172974
FUMm                    3.172975
MDHm                    3.168494
GAPD                    2.857815
PGK                     3.165480
TPI                     1.428909
MDH                     0.000000
PEP_Carboxylase         0.000000
PPCK                    0.000000
FBA                     1.428909
FBP                     0.000000
TKT2                    0.000000
RPE                     0.000000
Xylulokinase            0.000000
PYK_org                 0.000000
PYK                     2.857850
RPI                     0.000000
TKT1                    0.000000
TALA                    0.000000
PGM                     2.995291
ENO                     2.857887
GND                     0.000000
PGL                     0.000000
HEX1                    1.428907
PGI                     1.943454
G6PDH2r                 0.000000
PFK                     1.428907
PYRt2m                  2.857822
PDHm                    2.857853
ICL                     0.000000
MAS                     0.000000
PYRDC                   0.000000
ALDD2y                  0.000000
ALCD2X_copy1            0.000000
LactateDehydrogenase    0.000000
dtype: float64

<matplotlib.figure.Figure at 0x10eda9e10>
```

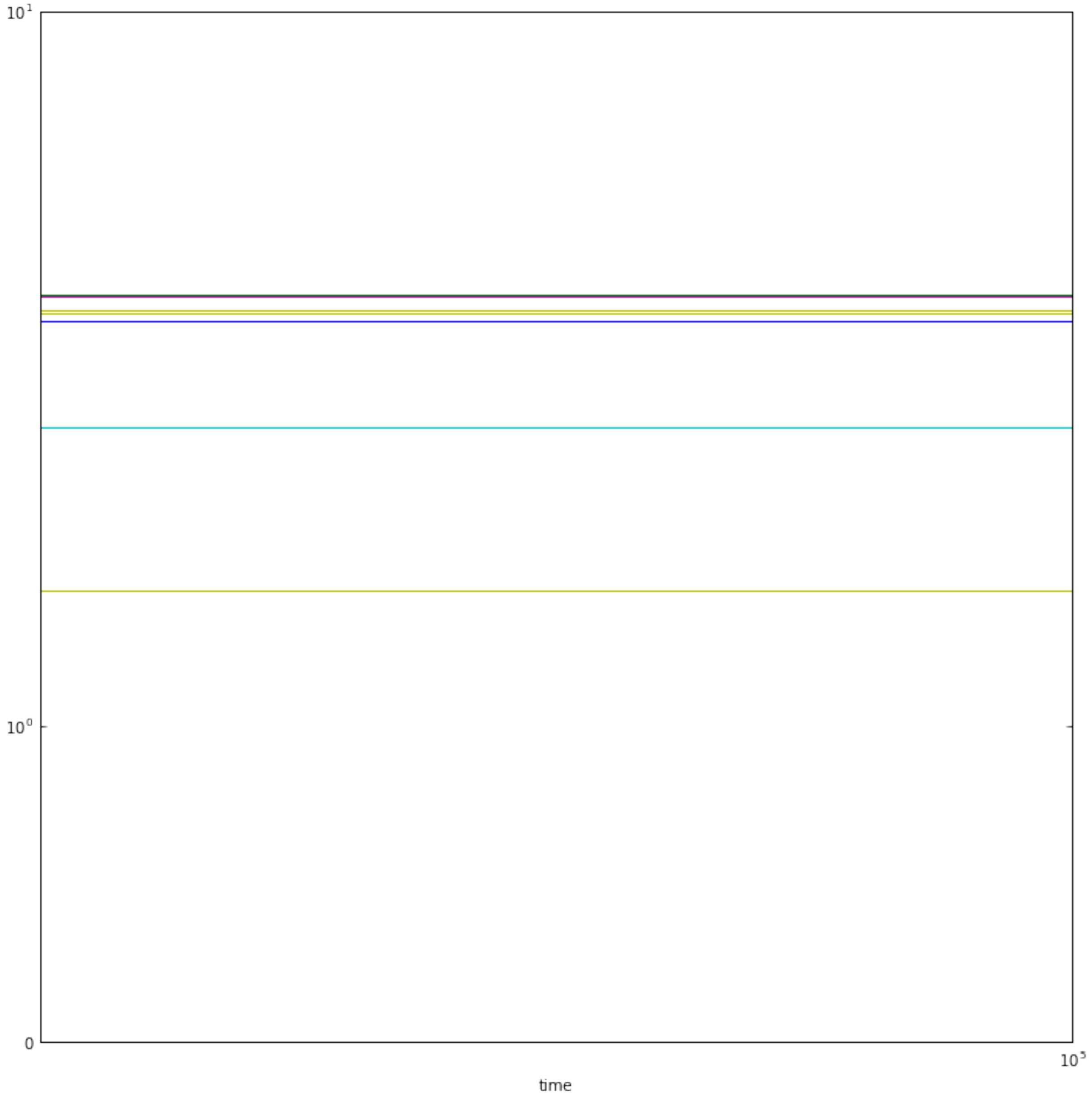

In [118]:

```
ode_likelihoods_steadystate = pd.DataFrame(data = ode_fwd_likelihoods_
timeseries.iloc[-20:].mean(),
                                          index = ode_fwd_likelihoods
_timeseries.columns,
                                         columns =['Forward'])
ode_likelihoods_steadystate['Reverse'] = ode_rev_likelihoods_timeserie
s.iloc[-20:].mean()
ode_likelihoods_steadystate['For-Rev'] = ode_likelihoods_steadystate['
Forward'] - ode_likelihoods_steadystate['Reverse']
ode_likelihoods_steadystate['Rxn Probabilities'] = ode_likelihoods_ste
adystate['For-Rev']/np.sum(abs(ode_likelihoods_steadystate['For-Rev'])
)
ode_likelihoods_steadystate
```

Out[118]:

	Forward	Reverse	For-Rev	Rxn Probabilities
**ME1m**	0.000000	0.000000e+00	0.000000	0.000000

**ME2m**	0.000000	0.000000e+00	0.000000	0.000000
**CSm**	3.167760	3.099452e-01	2.857815	0.057143
**ACONTm**	3.172948	3.151333e-01	2.857815	0.057143
**ICDHxm**	2.943128	8.531371e-02	2.857814	0.057143
**AKGDm**	2.943136	8.532161e-02	2.857814	0.057143
**SUCOASm**	3.172974	3.151601e-01	2.857814	0.057143
**SUCD1m**	3.172974	3.151600e-01	2.857814	0.057143
**FUMm**	3.172975	3.151608e-01	2.857814	0.057143
**MDHm**	3.168494	3.106795e-01	2.857815	0.057143
**GAPD**	2.857815	7.730613e-07	2.857814	0.057143
**PGK**	3.165480	3.076658e-01	2.857814	0.057143
**TPI**	1.428909	1.511438e-06	1.428907	0.028571
**MDH**	0.000000	0.000000e+00	0.000000	0.000000
**PEP_Carboxylase**	0.000000	0.000000e+00	0.000000	0.000000
**PPCK**	0.000000	0.000000e+00	0.000000	0.000000
**FBA**	1.428909	1.511434e-06	1.428907	0.028571
**FBP**	0.000000	0.000000e+00	0.000000	0.000000
**TKT2**	0.000000	0.000000e+00	0.000000	0.000000
**RPE**	0.000000	0.000000e+00	0.000000	0.000000
**Xylulokinase**	0.000000	0.000000e+00	0.000000	0.000000
**PYK_org**	0.000000	0.000000e+00	0.000000	0.000000
**PYK**	2.857850	3.583541e-05	2.857814	0.057143
**RPI**	0.000000	0.000000e+00	0.000000	0.000000
**TKT1**	0.000000	0.000000e+00	0.000000	0.000000
**TALA**	0.000000	0.000000e+00	0.000000	0.000000
**PGM**	2.995291	1.374770e-01	2.857814	0.057143
**ENO**	2.857887	7.274111e-05	2.857814	0.057143
**GND**	0.000000	0.000000e+00	0.000000	0.000000
**PGL**	0.000000	0.000000e+00	0.000000	0.000000
**HEX1**	1.428907	1.389075e-11	1.428907	0.028571
**PGI**	1.943454	5.145464e-01	1.428908	0.028571

**G6PDH2r**	0.000000	0.000000e+00	0.000000	0.000000
**PFK**	1.428907	1.389073e-11	1.428907	0.028571
**PYRt2m**	2.857822	8.046789e-06	2.857814	0.057143
**PDHm**	2.857853	3.886308e-05	2.857814	0.057143
**ICL**	0.000000	0.000000e+00	0.000000	0.000000
**MAS**	0.000000	0.000000e+00	0.000000	0.000000
**PYRDC**	0.000000	0.000000e+00	0.000000	0.000000
**ALDD2y**	0.000000	0.000000e+00	0.000000	0.000000
**ALCD2X_copy1**	0.000000	0.000000e+00	0.000000	0.000000
**LactateDehydrogenase**	0.000000	0.000000e+00	0.000000	0.000000

In [119]:

```
from escher import Builder

reaction_data = ode_likelihoods_steadystate['For-Rev'].to_dict()
b = Builder(map_name="iMM904.compact_Glycolysis_TCA_PPP.json",reaction
_data=reaction_data)
#b.display_in_browser(menu='all')
b.display_in_notebook(menu='zoom')
```

Out[119]:

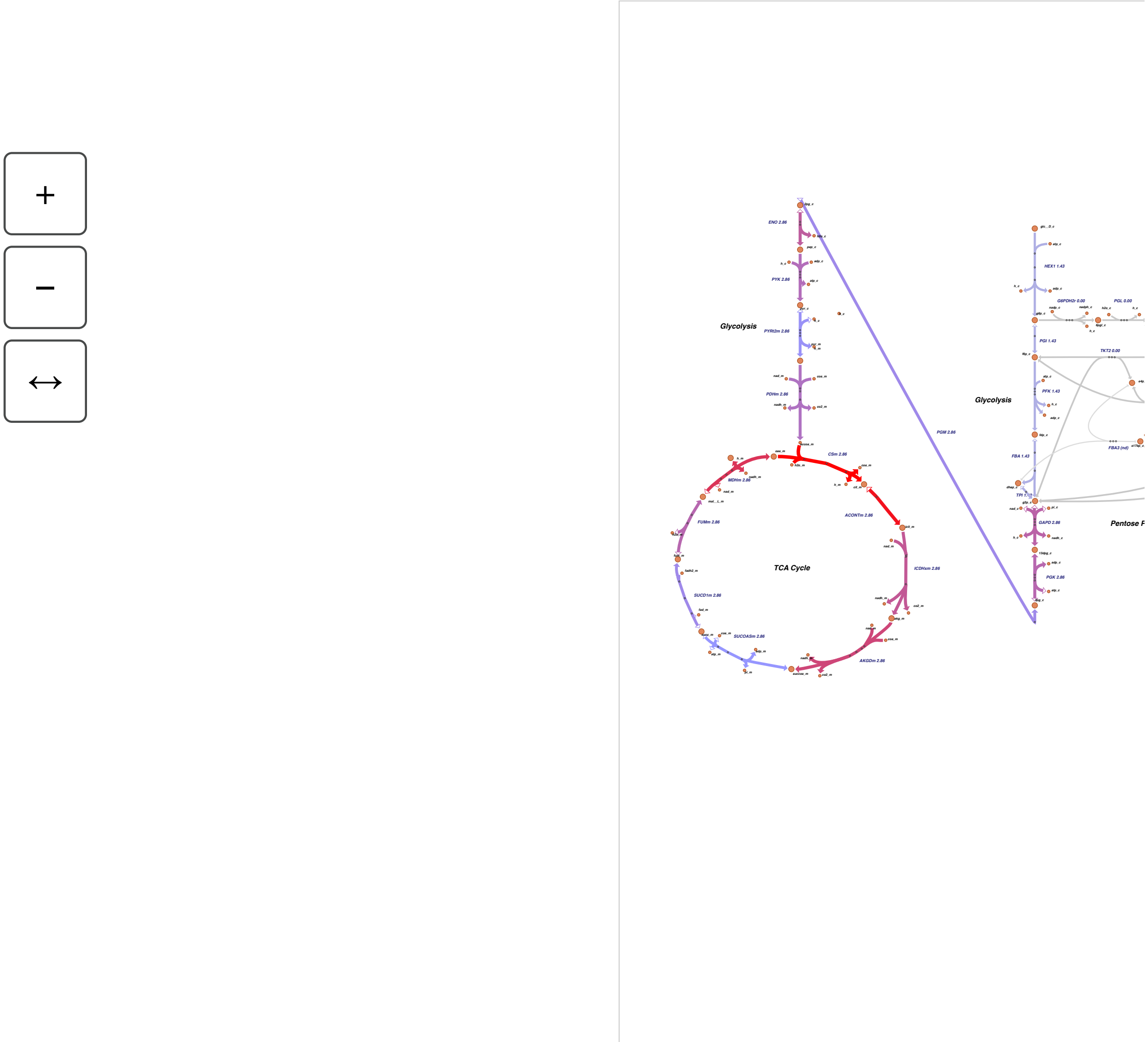

Analyze Metabolite Levels from Deterministic Simulation

In [120]:

```
# Read boltzmann stochastic reaction likelihoods
ode_metabolites = pd.read_table('neurospora_pentose_phos.glycolysis.tc
a.2_reg.ode_concs',header=0,index_col=0)

fig = plt.figure();
plot = ode_metabolites[variable_metabolite_idx].plot(legend=False,logy
=True,logx=True,figsize=(10, 10))
fig = plot.get_figure()
fig.savefig('metabolites_regulation.png')
plt.legend(bbox_to_anchor=(1.40, 0.90),bbox_transform=plt.gcf().transF
igure)
fig.savefig('metabolites_regulation_legend.png',bbox_inches='tight')
#plt.legend(bbox_to_anchor=(1.35, 0.95),bbox_transform=plt.gcf().trans
Figure)
```

```
<matplotlib.figure.Figure at 0x10b6e5780>
```

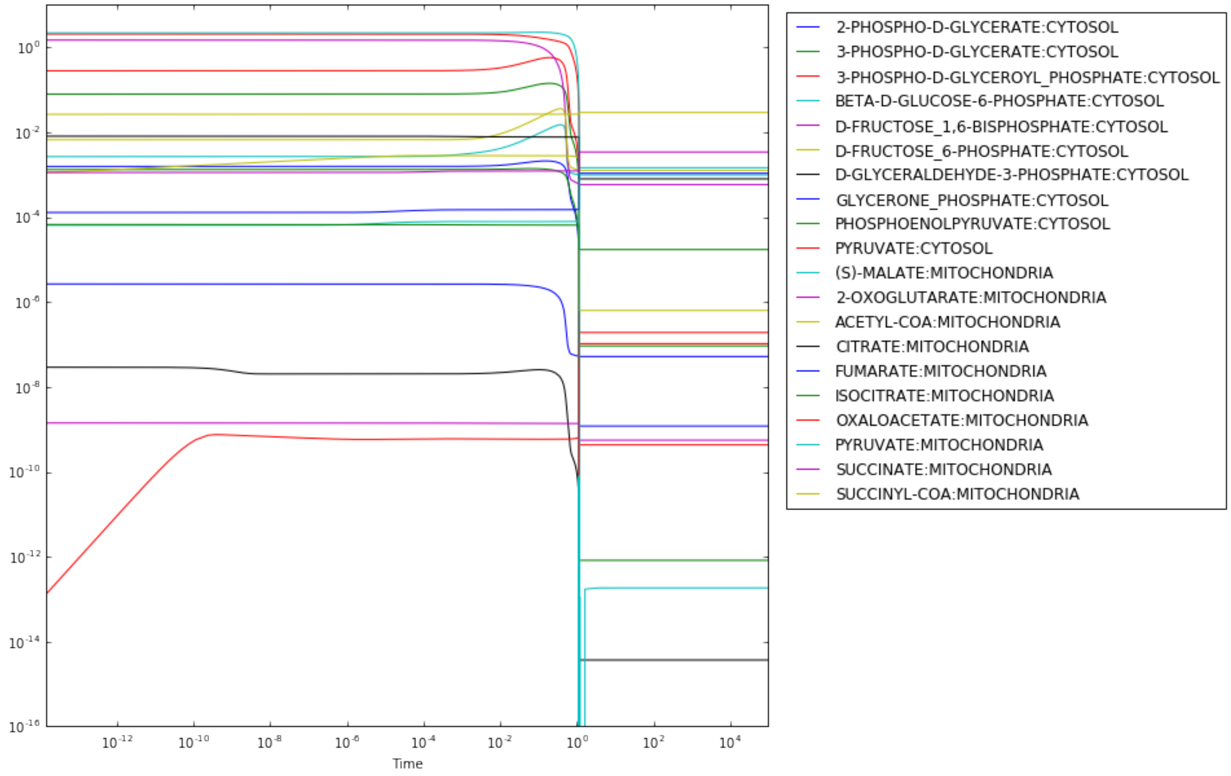

In [121]:
```
ode_metabolites_steadystate = \
  pd.DataFrame(data = ode_metabolites.iloc[-30:,:].mean(),columns=['OD
E'])
#display(ode_metabolites_steadystate[ode_metabolites_steadystate['ODE'
] > 1.0e-03])
ode_metabolites_steadystate['Counts'] = ode_metabolites_steadystate['O
DE']*Concentration2Count
display(ode_metabolites_steadystate.loc[variable_metabolite_idx,'ODE']
)
#conc_file = open('steady_state_concentrations.txt', 'w')
#print(ode_metabolites_steadystate,file=conc_file)
##for y in ode_metabolites_steadystate.index:
##    print(y,ode_metabolites.loc[y,:],file=conc_file)
#    #print(reaction_df[y])
#conc_file.close()
```

```
2-PHOSPHO-D-GLYCERATE:CYTOSOL               1.198412e-09
3-PHOSPHO-D-GLYCERATE:CYTOSOL               9.246162e-08
3-PHOSPHO-D-GLYCEROYL_PHOSPHATE:CYTOSOL     1.915114e-07
BETA-D-GLUCOSE-6-PHOSPHATE:CYTOSOL          9.827222e-04
D-FRUCTOSE_1,6-BISPHOSPHATE:CYTOSOL         5.848599e-04
D-FRUCTOSE_6-PHOSPHATE:CYTOSOL              1.262518e-03
D-GLYCERALDEHYDE-3-PHOSPHATE:CYTOSOL        3.700467e-15
GLYCERONE_PHOSPHATE:CYTOSOL                 5.215586e-08
PHOSPHOENOLPYRUVATE:CYTOSOL                 8.239296e-13
PYRUVATE:CYTOSOL                            4.321473e-10
(S)-MALATE:MITOCHONDRIA                     1.459268e-03
2-OXOGLUTARATE:MITOCHONDRIA                 5.568325e-10
ACETYL-COA:MITOCHONDRIA                     6.337797e-07
CITRATE:MITOCHONDRIA                        7.979930e-04
FUMARATE:MITOCHONDRIA                       1.078654e-03
ISOCITRATE:MITOCHONDRIA                     1.720703e-05
OXALOACETATE:MITOCHONDRIA                   1.048365e-07
PYRUVATE:MITOCHONDRIA                       1.849386e-13
SUCCINATE:MITOCHONDRIA                      3.422553e-03
SUCCINYL-COA:MITOCHONDRIA                   2.915238e-02
Name: ODE, dtype: float64
```

Infer Regulated Reactions

In [122]:

```
ode_metabolites_steadystate['Expected'] = 1.0e-03

S = pd.read_table('neurospora_pentose_phos.glycolysis.tca.2_reg.mat',h
eader=0, index_col = 0, quoting=2)
P = (S>0)
del P['forward reaction']

P = P.astype(np.float64)
#display(R)
product_concentrations = P.multiply(ode_metabolites_steadystate['ODE']
,axis=1)
product_concentrations[product_concentrations == 0] = 1
product_concentrations_rxns = pd.DataFrame(data = product_concentratio
ns.T.product(), columns=['ODE'])

expect_product_concentrations = P.multiply(ode_metabolites_steadystate
['Expected'],axis=1)
expect_product_concentrations[expect_product_concentrations == 0] = 1
product_concentrations_rxns['Expected'] = (expect_product_concentratio
ns.T).product()

s_regulation = '$\Delta S_{reg}$'
product_concentrations_rxns[s_regulation] = \
    np.log(product_concentrations_rxns['ODE']/product_concentrations_r
xns['Expected'])

idx = (ode_likelihoods_steadystate['Reverse'] != 0) & (ode_likelihoods
_steadystate['Forward'] != 0) & \
            (product_concentrations_rxns[s_regulation]>1)

display(product_concentrations_rxns[idx])
```

	ODE	Expected	ΔS_{reg}
**reaction title**			
**SUCOASm**	4.599911e-08	1.000000e-09	3.828622

Calculate Rate Constants

For a simple reaction,

$$A + B \rightleftharpoons C + D,$$

with forward rate constant k_{-1}, reverse rate constant k_{-1} and equilibrium constant $K_1 = \frac{k_1[A][B]}{k_{-1}[C][D]}$, the net flux $J_{1,net}$ is,

$$\begin{aligned} J_{1,net} &= \alpha \cdot k_1[A][B] - \alpha \cdot k_{-1}[C][D]] \\ &= \alpha \cdot k_1[A][B](1 - K_{-1}Q_{-1}^{-1}), \end{aligned}$$

where α is the activity of the enzyme as a function of regulation at any level. Solving for the rate constant k_1,

$$k_1 = \frac{J_{1,net}}{\alpha \cdot [A][B](1 - K_{-1}Q_{-1}^{-1})}.$$

Since $p_{1,net} \propto J_{1,net}$,

$$k_1 \propto \frac{p_{1,net}}{\alpha \cdot [A][B](1 - K_{-1}Q_{-1}^{-1})}.$$

The value of $K_{-1}Q_{-1}^{-1}$ should be the instantaneous value equal to $e^{A/RT}$ rather than equal to $e^{-\Delta G/RT}$. That is, in the reaction quotient the concentrations of the products should not be incremented by the stochiometric coefficients. The likelihoods printed out by Boltzmann are such that the product concentrations used to calculate the likelihood are incremented by the stoichiometric coefficients.

Note that the equations above do not require steady state dynamics.

Equalize reaction probabilities

In [123]:
```
x1 = 0.057143
x2 = 0.028571
delta = 0.000001
idx1 = (ode_likelihoods_steadystate['Rxn Probabilities'] > x1-delta) &
\
           (ode_likelihoods_steadystate['Rxn Probabilities'] < x1+delt
a)

idx2 = (ode_likelihoods_steadystate['Rxn Probabilities'] > x2-delta) &
\
           (ode_likelihoods_steadystate['Rxn Probabilities'] < x2+delt
a)
total = idx1.astype(np.float64).sum()*x1 + idx2.astype(np.float64).sum
()*x2
display(total)
ode_likelihoods_steadystate['Rxn Probabilities'][idx1] = x1/total
ode_likelihoods_steadystate['Rxn Probabilities'][idx2] = x2/total
display(ode_likelihoods_steadystate)
```

1.0

	Forward	Reverse	For-Rev	Rxn Probabilities
**ME1m**	0.000000	0.000000e+00	0.000000	0.000000
**ME2m**	0.000000	0.000000e+00	0.000000	0.000000
**CSm**	3.167760	3.099452e-01	2.857815	0.057143
**ACONTm**	3.172948	3.151333e-01	2.857815	0.057143
**ICDHxm**	2.943128	8.531371e-02	2.857814	0.057143
**AKGDm**	2.943136	8.532161e-02	2.857814	0.057143
**SUCOASm**	3.172974	3.151601e-01	2.857814	0.057143
**SUCD1m**	3.172974	3.151600e-01	2.857814	0.057143
**FUMm**	3.172975	3.151608e-01	2.857814	0.057143
**MDHm**	3.168494	3.106795e-01	2.857815	0.057143
**GAPD**	2.857815	7.730613e-07	2.857814	0.057143
**PGK**	3.165480	3.076658e-01	2.857814	0.057143
**TPI**	1.428909	1.511438e-06	1.428907	0.028571
**MDH**	0.000000	0.000000e+00	0.000000	0.000000
**PEP_Carboxylase**	0.000000	0.000000e+00	0.000000	0.000000
**PPCK**	0.000000	0.000000e+00	0.000000	0.000000
**FBA**	1.428909	1.511434e-06	1.428907	0.028571
**FBP**	0.000000	0.000000e+00	0.000000	0.000000
**TKT2**	0.000000	0.000000e+00	0.000000	0.000000
**RPE**	0.000000	0.000000e+00	0.000000	0.000000
**Xylulokinase**	0.000000	0.000000e+00	0.000000	0.000000
**PYK_org**	0.000000	0.000000e+00	0.000000	0.000000
**PYK**	2.857850	3.583541e-05	2.857814	0.057143
**RPI**	0.000000	0.000000e+00	0.000000	0.000000
**TKT1**	0.000000	0.000000e+00	0.000000	0.000000
**TALA**	0.000000	0.000000e+00	0.000000	0.000000
**PGM**	2.995291	1.374770e-01	2.857814	0.057143
**ENO**	2.857887	7.274111e-05	2.857814	0.057143
**GND**	0.000000	0.000000e+00	0.000000	0.000000

**PGL**	0.000000	0.000000e+00	0.000000	0.000000
**HEX1**	1.428907	1.389075e-11	1.428907	0.028571
**PGI**	1.943454	5.145464e-01	1.428908	0.028571
**G6PDH2r**	0.000000	0.000000e+00	0.000000	0.000000
**PFK**	1.428907	1.389073e-11	1.428907	0.028571
**PYRt2m**	2.857822	8.046789e-06	2.857814	0.057143
**PDHm**	2.857853	3.886308e-05	2.857814	0.057143
**ICL**	0.000000	0.000000e+00	0.000000	0.000000
**MAS**	0.000000	0.000000e+00	0.000000	0.000000
**PYRDC**	0.000000	0.000000e+00	0.000000	0.000000
**ALDD2y**	0.000000	0.000000e+00	0.000000	0.000000
**ALCD2X_copy1**	0.000000	0.000000e+00	0.000000	0.000000
**LactateDehydrogenase**	0.000000	0.000000e+00	0.000000	0.000000

Calculate the Unregulated Thermodynamic likelihood of the reactions

In order to calculate rate constants, it is necessary to calculate $e^{A_i/RT} = K_i Q_i^{-1}$, where A is the reaction affinity, K_i is the equilibrium constant, and Q_i is the reaction quotient for reaction i.

In [124]:
```
S = pd.read_table('neurospora_pentose_phos.glycolysis.tca.2_reg.mat',h
eader=0, index_col = 0, quoting=2)
#display(S)
R = (S<0)
P = S>0
del R['forward reaction']
del P['forward reaction']
del S['forward reaction']

R_values = np.abs(S[R])
R_values[R_values.isnull()] = 0
P_values = np.abs(S[P])
P_values[P_values.isnull()] = 0

#S['H2O:CYTOSOL'] =0
#S['H2O:GLYOXYSOME'] =0
#S['H2O:MITOCHONDRIA'] =0

volume_coeff = S.sum(axis=1)

R = R.astype(np.float64)
```

```
P = P.astype(np.float64)

steadyState_counts = ode_metabolites_steadystate['ODE']*Concentration2
Count

#reactant_counts = ode_metabolites_steadystate['ODE']*Concentration2Co
unt
reactant_counts = R.multiply(steadyState_counts,axis=1)
reactant_counts = reactant_counts.pow(R_values)
reactant_concs =  R.multiply(ode_metabolites_steadystate['ODE'])
reactant_concs = reactant_concs.pow(R_values)
product_counts = P.multiply(steadyState_counts,axis=1)
#display(reactant_counts)
product_concs = P.multiply(ode_metabolites_steadystate['ODE'])

# To the products, increment the current values by the stoichiometric
coefficient if comparing to the Boltzman
# likelihoods. But if using the computed likelihoods to calculate rate
constants, then don't increment.
product_counts_incremented = product_counts + P.multiply(1)
product_concs_incremented = product_concs + P.multiply(concentration_i
ncrement)
# For calculation of the reaction quotient, need to take each concentr
ation to the power of its stoichimetry:
product_counts = product_counts.pow(P_values)
product_counts_incremented = product_counts_incremented.pow(P_values)
product_concs = product_concs.pow(P_values)
product_concs_incremented = product_concs_incremented.pow(P_values)

#Need to increment reactant counts/concs for calculating likelihood of
reverse reaction!

# Take the product of counts of all reactants for a reaction
reactant_counts_rxns = (reactant_counts[reactant_counts != 0].T).produ
ct()
reactant_concs_rxns = (reactant_concs[reactant_concs != 0].T).product(
)

# Take the product of counts of all products for a reaction
product_counts_rxns = (product_counts[product_counts != 0].T).product(
)
product_concs_rxns = (product_concs[product_concs != 0].T).product()
product_counts_incremented_rxns = (product_counts_incremented[product_
counts_incremented != 0].T).product()
product_concs_incremented_rxns = (product_concs_incremented[product_co
ncs_incremented != 0].T).product()
#denominator = reactant_counts_rxns.multiply(1-ode_likelihoods_steadys
tate['Reverse'])

K_eq = pd.read_table('neurospora_pentose_phos.glycolysis.tca.2_reg.dg0
ke',header=0, index_col = 0, quoting=2)

# Calculate rate constants
```

```
result = pd.DataFrame()
result['Boltzmann Ke'] = K_eq['Ke']
# Notice that Computed L_fwd uses incremented product counts. That is
so we can compare to Boltzmann values
# when calculating the activities. That is, these need to use the same
formula.
result['Computed L_fwd'] = result['Boltzmann Ke'].multiply(reactant_co
unts_rxns.divide(product_counts_incremented_rxns))
# In contrast to Computed L_fwd, Computed L_rev does not use increment
ed product counts when calculating the value.
# This is because we need Computed L_rev to look like the exponent of
the reaction affinity, not the free
# energy change. See markup above.
result['Computed L_rev'] = (product_counts_rxns.divide(reactant_counts
_rxns)).divide(result['Boltzmann Ke'])

result['Boltzmann L_fwd'] = ode_likelihoods_steadystate['Forward']
result['Boltzmann L_rev'] = ode_likelihoods_steadystate['Reverse']
result['steady state activity'] = result['Boltzmann L_fwd'].divide(res
ult['Computed L_fwd'])
result['Adjusted L_fwd'] = result['Computed L_fwd'].multiply(result['s
teady state activity'])
display(result)
```

	**Boltzmann Ke**	**Computed L_fwd**	**Computed L_rev**	**Boltzmann L_fwd**	**Bol**
**Rxn_title**					
**ME1m**	5.807996e-02	2.654559e+01	4.194902e-06	0.000000	0.0000
**ME2m**	5.733443e-02	1.463953e-02	7.606588e-03	0.000000	0.0000
**CSm**	5.326355e+07	3.167761e+00	3.156793e-01	3.167760	3.0994
**ACONTm**	6.842451e-02	3.172948e+00	3.151339e-01	3.172948	3.1513
**ICDHxm**	7.291438e-01	2.943128e+00	8.532202e-02	2.943128	8.5313
**AKGDm**	3.513601e+05	2.943136e+00	3.397611e-01	2.943136	8.5321
**SUCOASm**	4.470177e-01	3.172974e+00	3.151612e-01	3.172974	3.1516
**SUCD1m**	1.000000e+00	3.172975e+00	3.151606e-01	3.172974	3.1516
**FUMm**	4.292596e+00	3.172975e+00	3.151613e-01	3.172975	3.1516
**MDHm**	7.381907e-06	3.168492e+00	3.106802e-01	3.168494	3.1067
**GAPD**	3.954160e-01	2.857816e+00	3.469027e-01	2.857815	7.7306
**PGK**	2.666982e+01	3.165480e+00	3.103344e-01	3.165480	3.0766
**TPI**	4.549371e-02	1.428908e+00	1.559560e-06	1.428909	1.5114

**MDH**	7.381907e-06	3.931003e-04	2.543825e+03	0.000000	0.0000
**PEP_Carboxylase**	2.755360e-08	3.316932e+00	1.495140e-04	0.000000	0.0000
**PPCK**	6.322968e-02	1.083379e+01	4.577589e-05	0.000000	0.0000
**FBA**	1.314826e-04	1.428909e+00	1.511438e-06	1.428909	1.5114
**FBP**	3.776842e-03	9.819610e+04	1.018368e-05	0.000000	0.0000
**TKT2**	1.503324e-01	5.689675e-06	1.701447e+05	0.000000	0.0000
**RPE**	1.368145e-01	1.092173e+00	9.103757e-01	0.000000	0.0000
**Xylulokinase**	2.485987e+05	1.614927e+10	6.187785e-11	0.000000	0.0000
**PYK_org**	1.244333e+05	2.857849e+00	7.225839e-02	0.000000	0.0000
**PYK**	1.244333e+05	2.857849e+00	7.225839e-02	2.857850	3.5835
**RPI**	8.306288e-01	9.113205e-01	1.091041e+00	0.000000	0.0000
**TKT1**	1.076262e+00	1.623681e-06	6.122565e+05	0.000000	0.0000
**TALA**	3.264645e+01	5.463896e-07	1.773020e+06	0.000000	0.0000
**PGM**	9.261562e-02	2.995291e+00	1.399460e-01	2.995291	1.3747
**ENO**	3.961900e+00	2.857888e+00	1.735323e-04	2.857887	7.2741
**GND**	3.711786e+02	2.221550e+11	4.497805e-12	0.000000	0.0000
**PGL**	1.337354e-07	1.337354e-07	7.477452e+06	0.000000	0.0000
**HEX1**	9.193007e+03	3.207288e+05	3.117884e-06	1.428907	1.3890
**PGI**	2.496787e+00	1.943453e+00	5.145473e-01	1.943454	5.1454
**G6PDH2r**	1.737796e+00	1.799751e+04	5.556246e-05	0.000000	0.0000
**PFK**	8.667054e+03	3.207288e+05	3.117881e-06	1.428907	1.3890
**PYRt2m**	1.098249e+01	2.857822e+00	3.896682e-05	2.857822	8.0467
**PDHm**	2.239131e+04	2.857852e+00	3.489860e-01	2.857853	3.8863
**ICL**	1.055610e+00	3.070124e-03	3.251788e+02	0.000000	0.0000
**MAS**	1.914360e+08	2.302898e+05	4.342346e-06	0.000000	0.0000
**PYRDC**	9.866757e+01	4.263822e-04	2.345275e+03	0.000000	0.0000
**ALDD2y**	3.587265e+09	6.277627e+07	1.592937e-08	0.000000	0.0000
**ALCD2X_copy1**	8.922578e+03	2.848360e+02	3.510790e-03	0.000000	0.0000
**LactateDehydrogenase**	3.030051e+03	2.517312e+01	3.972488e-02	0.000000	0.0000

Calculate Rate Constants/Parameters

The program Boltzmann can formulate the ODEs for the time dependence of metabolites using either metabolite levels in counts or concentrations. The rate parameters will differ depending on whether counts or concentrations are used. Below both types of rate parameters are calculated, but in the end, the rate parameters using counts are output to a file to be used as input for Boltzmann.

In [125]:

```
# Use concs or counts below.
denominator_concs = reactant_concs_rxns.multiply(1-result['Computed L_rev'])
denominator_concs = denominator_concs.multiply(result['steady state activity'])
denominator_counts = reactant_counts_rxns.multiply(1-result['Computed L_rev'])
denominator_counts = denominator_counts.multiply(result['steady state activity']) #
result['Fwd Rate Constants Counts'] = ode_likelihoods_steadystate['Rxn Probabilities'].divide(denominator_counts)
result['Fwd Rate Constants Concs'] = ode_likelihoods_steadystate['Rxn Probabilities'].divide(denominator_counts)
idx = result['Fwd Rate Constants Counts'].isnull()
result['Fwd Rate Constants Counts'].loc[idx] = 0
result['Fwd Rate Constants Concs'].loc[idx] = 0
result['Rev Rate Constants Counts'] = result['Fwd Rate Constants Counts'].divide(K_eq['Ke'])
# The equilibrium constant in Boltzmann is formulated in terms of counts. If concentrations are used instead
# then the equilibrium constant needs to be multiplied by the Volume of the compartment raised to the
# negative sum of the stoichiometric coefficients.
result['Rev Rate Constants Concs'] = result['Fwd Rate Constants Concs'].divide(K_eq['Ke']*np.power(VolCell,volume_coeff))
fwd_rates = (result['Fwd Rate Constants Counts'].multiply(reactant_counts_rxns)).multiply(result['steady state activity'])
rev_rates = (result['Rev Rate Constants Counts'].multiply(product_counts_rxns)).multiply(result['steady state activity'])

#fwd_rates = (result['Fwd Rate Constants Concs'].multiply(reactant_concs_rxns)).multiply(result['steady state activity'])
#rev_rates = (result['Rev Rate Constants Concs'].multiply(product_concs_rxns)).multiply(result['steady state activity'])

result['Computed Ke'] = result['Fwd Rate Constants Counts']/result['Rev Rate Constants Counts']
result['Fwd Rate'] = fwd_rates
result['Rev Rate'] = rev_rates
result['Rxn Flux'] = fwd_rates-rev_rates
display(result)
```

	Boltzmann Ke	Computed L_fwd	Computed L_rev	...	Fwd Rate	[illegible]
**Rxn_title**						
**ME1m**	5.807996e-02	2.654559e+01	4.194902e-06	...	0.000000	0.000
**ME2m**	5.733443e-02	1.463953e-02	7.606588e-03	...	0.000000	0.000
**CSm**	5.326355e+07	3.167761e+00	3.156793e-01	...	0.083503	2.636
**ACONTm**	6.842451e-02	3.172948e+00	3.151339e-01	...	0.083437	2.629
**ICDHxm**	7.291438e-01	2.943128e+00	8.532202e-02	...	0.062473	5.330
**AKGDm**	3.513601e+05	2.943136e+00	3.397611e-01	...	0.086549	2.940
**SUCOASm**	4.470177e-01	3.172974e+00	3.151612e-01	...	0.083440	2.629
**SUCD1m**	1.000000e+00	3.172975e+00	3.151606e-01	...	0.083440	2.629
**FUMm**	4.292596e+00	3.172975e+00	3.151613e-01	...	0.083440	2.629
**MDHm**	7.381907e-06	3.168492e+00	3.106802e-01	...	0.082898	2.575
**GAPD**	3.954160e-01	2.857816e+00	3.469027e-01	...	0.087495	3.035
**PGK**	2.666982e+01	3.165480e+00	3.103344e-01	...	0.082856	2.571
**TPI**	4.549371e-02	1.428908e+00	1.559560e-06	...	0.028571	4.455
**MDH**	7.381907e-06	3.931003e-04	2.543825e+03	...	0.000000	0.000
**PEP_Carboxylase**	2.755360e-08	3.316932e+00	1.495140e-04	...	0.000000	0.000
**PPCK**	6.322968e-02	1.083379e+01	4.577589e-05	...	0.000000	0.000
**FBA**	1.314826e-04	1.428909e+00	1.511438e-06	...	0.028571	4.318
**FBP**	3.776842e-03	9.819610e+04	1.018368e-05	...	0.000000	0.000
**TKT2**	1.503324e-01	5.689675e-06	1.701447e+05	...	0.000000	0.000
**RPE**	1.368145e-01	1.092173e+00	9.103757e-01	...	0.000000	0.000
**Xylulokinase**	2.485987e+05	1.614927e+10	6.187785e-11	...	0.000000	0.000
**PYK_org**	1.244333e+05	2.857849e+00	7.225839e-02	...	0.000000	0.000
**PYK**	1.244333e+05	2.857849e+00	7.225839e-02	...	0.061594	4.450
**RPI**	8.306288e-01	9.113205e-01	1.091041e+00	...	0.000000	0.000
**TKT1**	1.076262e+00	1.623681e-06	6.122565e+05	...	0.000000	0.000
**TALA**	3.264645e+01	5.463896e-07	1.773020e+06	...	0.000000	0.000
**PGM**	9.261562e-02	2.995291e+00	1.399460e-01	...	0.066441	9.298
**ENO**	3.961900e+00	2.857888e+00	1.735323e-04	...	0.057153	9.917
**GND**	3.711786e+02	2.221550e+11	4.497805e-12	...	0.000000	0.000

**PGL**	1.337354e-07	1.337354e-07	7.477452e+06	...	0.000000	0.000
**HEX1**	9.193007e+03	3.207288e+05	3.117884e-06	...	0.028571	8.908
**PGI**	2.496787e+00	1.943453e+00	5.145473e-01	...	0.058854	3.028
**G6PDH2r**	1.737796e+00	1.799751e+04	5.556246e-05	...	0.000000	0.000
**PFK**	8.667054e+03	3.207288e+05	3.117881e-06	...	0.028571	8.908
**PYRt2m**	1.098249e+01	2.857822e+00	3.896682e-05	...	0.057145	2.226
**PDHm**	2.239131e+04	2.857852e+00	3.489860e-01	...	0.087775	3.063
**ICL**	1.055610e+00	3.070124e-03	3.251788e+02	...	0.000000	0.000
**MAS**	1.914360e+08	2.302898e+05	4.342346e-06	...	0.000000	0.000
**PYRDC**	9.866757e+01	4.263822e-04	2.345275e+03	...	0.000000	0.000
**ALDD2y**	3.587265e+09	6.277627e+07	1.592937e-08	...	0.000000	0.000
**ALCD2X_copy1**	8.922578e+03	2.848360e+02	3.510790e-03	...	0.000000	0.000
**LactateDehydrogenase**	3.030051e+03	2.517312e+01	3.972488e-02	...	0.000000	0.000

42 rows × 15 columns

Cutoff for time derivative of concentrations

The simulation program needs to know when a derivative should be considered to be effectively zero. This is determined by the accuracy of the estimates of the rate constants above. We can calculate this accuracy by looking at the difference between the net fluxes of all reactions. This gives a set of m reactions by m reactions differences. Then for each reaction, find the other reaction that has the largest difference. This gives a set of m reactions by 1 differences. Then for each of the reactions, find the largest global difference. This is then the global cutoff for when the derivatives should be considered to be effectively zero.

In [126]:

```
diff = pd.DataFrame(columns = result.index)
for col in diff.columns:
   diff[col] = result['Rxn Flux']
#Compare all reaction fluxes against all reaction fluxes
diff = diff - diff.T
# eliminate teh reactions that have large differences because they are
on different sides of a branch
diff[diff.abs() > 0.001] =0
display(diff.max())
display('Max = ',diff.max().max())
```

```
Rxn_title
ME1m                     0.000000e+00
ME2m                     0.000000e+00
CSm                      2.775558e-17
ACONTm                   0.000000e+00
ICDHxm                   2.081668e-17
AKGDm                    1.387779e-17
SUCOASm                  1.387779e-17
SUCD1m                   2.081668e-17
FUMm                     2.081668e-17
MDHm                     2.081668e-17
GAPD                     0.000000e+00
PGK                      6.938894e-18
TPI                      0.000000e+00
MDH                      0.000000e+00
PEP_Carboxylase          0.000000e+00
PPCK                     0.000000e+00
FBA                      0.000000e+00
FBP                      0.000000e+00
TKT2                     0.000000e+00
RPE                      0.000000e+00
Xylulokinase             0.000000e+00
PYK_org                  0.000000e+00
PYK                      1.387779e-17
RPI                      0.000000e+00
TKT1                     0.000000e+00
TALA                     0.000000e+00
PGM                      2.081668e-17
ENO                      6.938894e-18
GND                      0.000000e+00
PGL                      0.000000e+00
HEX1                     0.000000e+00
PGI                      6.938894e-18
G6PDH2r                  0.000000e+00
PFK                      3.469447e-18
PYRt2m                   6.938894e-18
PDHm                     2.081668e-17
ICL                      0.000000e+00
MAS                      0.000000e+00
PYRDC                    0.000000e+00
ALDD2y                   0.000000e+00
ALCD2X_copy1             0.000000e+00
LactateDehydrogenase     0.000000e+00
dtype: float64

'Max = '

2.7755575615628914e-17
```

In [127]:
```
reaction_df = pd.DataFrame()

filename = '/Users/d3k137/docs/projects/boltzmann/code/06212017/run/pe
ntose_phos.glycolysis.tca/neurospora_pentose_phos.glycolysis.tca.2.dat
'
with open(filename,'r') as f:
  for line in f:
     #print(line)
     if re.match('^REACTION', line):
        temp = re.split('\s',line,1)
        rxn_name = temp[1].strip()
        if not rxn_name:
          print("Error: Reaction name not found:\n", line)
     elif re.match('^COMMENT',line):
        continue
     elif re.match(r'//',line):
        continue
     elif re.match('^#',line):
        continue
     else:
        #print(line)
        rxn_pair = re.split('\s',line,1)
        reaction_df.loc[rxn_pair[0],rxn_name] = rxn_pair[1].strip()

     # end if
  # end for loop
# end with loop
display(reaction_df)
```

	ME1m	ME2m	CSm	...	A
**LEFT**	(S)-MALATE + NAD+	(S)-MALATE + NADP+	OXALOACETATE + ACETYL-COA + H2O	...	acetal + NAD H2O
**RIGHT**	pyruvate + NADH + CO2	PYRUVATE + NADPH + CO2	CITRATE + COA	...	acetat NADP
**LEFT_COMPARTMENT**	MITOCHONDRIA	MITOCHONDRIA	MITOCHONDRIA	...	CYTO
**RIGHT_COMPARTMENT**	MITOCHONDRIA	MITOCHONDRIA	MITOCHONDRIA	...	CYTO
**ENZYME_LEVEL**	0.0	0.0	NaN	...	0.0
**PATHWAY**	NaN	NaN	NaN	...	fermer
**NREGULATION**	NaN	NaN	NaN	...	NaN
**COMMNENT**	NaN	NaN	NaN	...	NaN
**DGZERO**	NaN	NaN	NaN	...	NaN
**DGZERO-UNITS**	NaN	NaN	NaN	...	NaN

10 rows × 42 columns

In [128]:

```
reaction_file = open('neurospora_pentose_phos.glycolysis.tca.2_rate.da
t', 'w')
for y in reaction_df:
    print("REACTION\t",y,file=reaction_file)
    #print(reaction_df[y])
    for x in reaction_df[y].index:
      if pd.notnull(reaction_df.loc[x,y]):
        print(x, reaction_df.loc[x,y],file=reaction_file)
    print("k_FORWARD",result.loc[y,'Fwd Rate Constants Counts'],file=r
eaction_file)
    print("k_REVERSE",result.loc[y,'Rev Rate Constants Counts'],file=r
eaction_file)
    print("//",file=reaction_file)
reaction_file.close()
```
