# Supplementary material for: Prediction of Metabolite Concentrations, Rate Constants and Post-Translational Regulation Using Maximum Entropy-Based Simulations with Application to Central Metabolism of Neurospora crassa
Source: Processes (Basel). Author manuscript; Available in PMC 2021 Apr 5. (PMC8020867; doi:10.3390/pr6060063)
Supplement: NotebookS3 [file NIHMS1061468-supplement-NotebookS3.pdf]

Table of Contents

1 Boltzmann
1.1 Pentose phosphate + Glycolysis + TCA cycle
1.2 Turning on Regulation and Setting Enzyme Levels
2 Models and Simulations
2.1 Model With Regulation
2.1.1 Run Deterministic Simulation
2.1.2 Derivatives from ODE kinetic rate law simulation
2.1.3 Forward and Reverse Rates from Deterministic Simulation
2.2 Analyze Metabolite Levels from Deterministic Simulation
2.3 Reaction Rates from Kinetic Rate Law
2.4 Calculate Power
2.4.1 Power Characteristics
2.5 Calculate Resistance and Conductance

In [14]:
```
import numpy as np
import pandas as pd
import matplotlib.pyplot as plt
import matplotlib
import subprocess
import re
import os
from IPython.core.display import display
pd.set_option('display.max_columns', 7,'display.max_rows', None)
#from ipynb_latex_setup import *
%matplotlib inline
N_avogadro = 6.022140857e+23
VolCell = 1.0e-15
Concentration2Count = N_avogadro * VolCell
concentration_increment = 1/(N_avogadro*VolCell)
RT = 8.3144598e-03 * 298.15
```

Change directories to where the simulation data is:

In [15]:
```
# Comment/uncomment to change to your directory, if needed:
cwd = os.getcwd()
if 'simulation_data' not in cwd:
  mydir = cwd+'/simulation_data'
  os.chdir(mydir)
```

Boltzmann

A beta version of the simulation code is available on GitHub at https://github.com/PNNL-CompBio/Boltzmann (https://github.com/PNNL-CompBio/Boltzmann).

Pentose phosphate + Glycolysis + TCA cycle

Reactions:

In [16]:
```
cat neurospora_pentose_phos.glycolysis.tca.2_rate.dat
```

```
REACTION         ME1m
LEFT (S)-MALATE + NAD+
RIGHT pyruvate + NADH + CO2
LEFT_COMPARTMENT MITOCHONDRIA
RIGHT_COMPARTMENT MITOCHONDRIA
ENZYME_LEVEL 0.0
k_FORWARD 0.0
k_REVERSE 0.0
//
REACTION         ME2m
LEFT (S)-MALATE + NADP+
RIGHT PYRUVATE + NADPH + CO2
LEFT_COMPARTMENT MITOCHONDRIA
RIGHT_COMPARTMENT MITOCHONDRIA
ENZYME_LEVEL 0.0
k_FORWARD 0.0
k_REVERSE 0.0
//
REACTION         CSm
LEFT OXALOACETATE + ACETYL-COA + H2O
RIGHT CITRATE + COA
LEFT_COMPARTMENT MITOCHONDRIA
RIGHT_COMPARTMENT MITOCHONDRIA
k_FORWARD 3.46538006973e-06
k_REVERSE 6.50610045655e-14
//
REACTION         ACONTm
LEFT CITRATE
RIGHT ISOCITRATE
LEFT_COMPARTMENT MITOCHONDRIA
RIGHT_COMPARTMENT MITOCHONDRIA
k_FORWARD 1.73623049906e-07
k_REVERSE 2.53743943371e-06
//
REACTION         ICDHxm
LEFT ISOCITRATE + NAD+
RIGHT 2-OXOGLUTARATE + NADH + CO2
LEFT_COMPARTMENT MITOCHONDRIA
```

```
RIGHT_COMPARTMENT MITOCHONDRIA
k_FORWARD 3.85046925276e-12
k_REVERSE 5.28080915281e-12
//
REACTION         AKGDm
LEFT 2-OXOGLUTARATE + COA + NAD+
RIGHT SUCCINYL-COA + CO2 + NADH
LEFT_COMPARTMENT MITOCHONDRIA
RIGHT_COMPARTMENT MITOCHONDRIA
k_FORWARD 1.95516451027e-13
k_REVERSE 5.56456043321e-19
//
REACTION         SUCOASm
LEFT SUCCINYL-COA + ADP + Orthophosphate
RIGHT SUCCINATE + ATP + COA
LEFT_COMPARTMENT MITOCHONDRIA
RIGHT_COMPARTMENT MITOCHONDRIA
k_FORWARD 1.17011859566e-21
k_REVERSE 2.61761132872e-21
//
REACTION         SUCD1m
LEFT SUCCINATE + redox1
RIGHT FUMARATE + redox2
LEFT_COMPARTMENT MITOCHONDRIA
RIGHT_COMPARTMENT MITOCHONDRIA
k_FORWARD 6.72237050766e-14
k_REVERSE 6.72237050766e-14
//
REACTION         FUMm
LEFT FUMARATE + H2O
RIGHT (S)-MALATE
LEFT_COMPARTMENT MITOCHONDRIA
RIGHT_COMPARTMENT MITOCHONDRIA
k_FORWARD 1.28452229613e-07
k_REVERSE 2.99241367259e-08
//
REACTION         MDHm
LEFT (S)-MALATE + NAD+
RIGHT OXALOACETATE + NADH
LEFT_COMPARTMENT MITOCHONDRIA
RIGHT_COMPARTMENT MITOCHONDRIA
k_FORWARD 6.02465046406e-14
k_REVERSE 8.16137410571e-09
//
REACTION         GAPD
LEFT D-GLYCERALDEHYDE-3-PHOSPHATE + ORTHOPHOSPHATE + NAD+
RIGHT 3-Phospho-D-glyceroyl_phosphate + NADH
LEFT_COMPARTMENT CYTOSOL
RIGHT_COMPARTMENT CYTOSOL
ENZYME_LEVEL 1.0
PATHWAY GLUCONEOGENESIS, GLYCOLYSIS
k_FORWARD 2.08196126641e-09
k_REVERSE 5.26524284909e-09
```

```
//
REACTION        PGK
LEFT 3-Phospho-D-glyceroyl_phosphate + ADP
RIGHT 3-PHOSPHO-D-GLYCERATE + ATP
LEFT_COMPARTMENT CYTOSOL
RIGHT_COMPARTMENT CYTOSOL
ENZYME_LEVEL 1.0
PATHWAY GLUCONEOGENESIS, GLYCOLYSIS, CALVIN CYCLE
k_FORWARD 2.13029627082e-09
k_REVERSE 7.987666474e-11
//
REACTION        TPI
LEFT Glycerone_phosphate
RIGHT D-GLYCERALDEHYDE-3-PHOSPHATE
LEFT_COMPARTMENT CYTOSOL
RIGHT_COMPARTMENT CYTOSOL
ENZYME_LEVEL 1.0
PATHWAY CALVIN CYCLE, GLYCOLYSIS
k_FORWARD 0.000909644994161
k_REVERSE 0.0199949618125
//
REACTION        MDH
LEFT (S)-MALATE + NAD+
RIGHT OXALOACETATE + NADH
LEFT_COMPARTMENT CYTOSOL
RIGHT_COMPARTMENT CYTOSOL
ENZYME_LEVEL 0.0
k_FORWARD 0.0
k_REVERSE 0.0
//
REACTION        PEP_Carboxylase
LEFT oxaloacetate + orthophosphate
RIGHT phosphoenolpyruvate + CO2 + H2O
LEFT_COMPARTMENT CYTOSOL
RIGHT_COMPARTMENT CYTOSOL
ENZYME_LEVEL 0.0
PATHWAY GLUCONEOGENSESIS
k_FORWARD 0.0
k_REVERSE 0.0
//
REACTION        PPCK
LEFT oxaloacetate + ATP
RIGHT phosphoenolpyruvate + ADP + CO2
LEFT_COMPARTMENT CYTOSOL
RIGHT_COMPARTMENT CYTOSOL
ENZYME_LEVEL 0.0
PATHWAY GLUCONEOGENSESIS
k_FORWARD 0.0
k_REVERSE 0.0
//
REACTION        FBA
LEFT D-FRUCTOSE_1,6-BISPHOSPHATE
RIGHT Glycerone_phosphate + D-GLYCERALDEHYDE-3-PHOSPHATE
```

```
LEFT_COMPARTMENT CYTOSOL
RIGHT_COMPARTMENT CYTOSOL
ENZYME_LEVEL 1.0
PATHWAY GLUCONEOGENSESIS, GLYCOLYSIS, CALVIN CYCLE
k_FORWARD 8.11191324392e-08
k_REVERSE 0.000616957167254
//
REACTION         FBP
LEFT D-FRUCTOSE_6-PHOSPHATE + Orthophosphate
RIGHT H2O + D-FRUCTOSE_1,6-BISPHOSPHATE
LEFT_COMPARTMENT CYTOSOL
RIGHT_COMPARTMENT CYTOSOL
ENZYME_LEVEL 0.0
PATHWAY GLUCONEOGENESIS, CALVIN CYCLE
k_FORWARD 0.0
k_REVERSE 0.0
//
REACTION         TKT2
LEFT D-FRUCTOSE_6-PHOSPHATE + D-GLYCERALDEHYDE-3-PHOSPHATE
RIGHT D-ERYTHROSE-4-PHOSPHATE + D-XYLULOSE-5-PHOSPHATE
LEFT_COMPARTMENT CYTOSOL
RIGHT_COMPARTMENT CYTOSOL
ENZYME_LEVEL 0.0
PATHWAY CALVIN CYCLE, PENTOSE PHOSPHATE PATHWAY, RUBISCO SHUNT
k_FORWARD 0.0
k_REVERSE 0.0
//
REACTION         RPE
LEFT D-XYLULOSE-5-PHOSPHATE
RIGHT D-RIBULOSE-5-PHOSPHATE
LEFT_COMPARTMENT CYTOSOL
RIGHT_COMPARTMENT CYTOSOL
ENZYME_LEVEL 0.0
PATHWAY CALVIN CYCLE, PENTOSE PHOSPHATE PATHWAY, RUBISCO SHUNT
k_FORWARD 0.0
k_REVERSE 0.0
//
REACTION         Xylulokinase
LEFT D-XYLULOSE + ATP
RIGHT D-XYLULOSE-5-PHOSPHATE + ADP
LEFT_COMPARTMENT CYTOSOL
RIGHT_COMPARTMENT CYTOSOL
ENZYME_LEVEL 0.0
PATHWAY CALVIN CYCLE, PENTOSE PHOSPHATE PATHWAY, RUBISCO SHUNT
k_FORWARD 0.0
k_REVERSE 0.0
//
REACTION         PYK_org
LEFT ADP + PHOSPHOENOLPYRUVATE
RIGHT PYRUVATE + ATP
LEFT_COMPARTMENT CYTOSOL
RIGHT_COMPARTMENT CYTOSOL
ENZYME_LEVEL 0.0
```

```
PATHWAY GLYCOLYSIS, PYRUVATE METABOLISM, RUBISCO SHUNT
k_FORWARD 0.0
k_REVERSE 0.0
//
REACTION         PYK
LEFT ADP + PHOSPHOENOLPYRUVATE
RIGHT PYRUVATE + ATP
LEFT_COMPARTMENT CYTOSOL
RIGHT_COMPARTMENT CYTOSOL
ENZYME_LEVEL 1.0
PATHWAY GLYCOLYSIS, PYRUVATE METABOLISM, RUBISCO SHUNT
k_FORWARD 0.000368091614665
k_REVERSE 2.95814395877e-09
//
REACTION         RPI
LEFT D-RIBOSE-5-PHOSPHATE
RIGHT D-RIBULOSE-5-PHOSPHATE
LEFT_COMPARTMENT CYTOSOL
RIGHT_COMPARTMENT CYTOSOL
ENZYME_LEVEL 0.0
PATHWAY CALVIN CYCLE, PENTOSE PHOSPHATE PATHWAY, RUBISCO SHUNT
k_FORWARD 0.0
k_REVERSE 0.0
//
REACTION         TKT1
LEFT SEDOHEPTULOSE_7-PHOSPHATE + D-GLYCERALDEHYDE-3-PHOSPHATE
RIGHT D-RIBOSE-5-PHOSPHATE + D-XYLULOSE-5-PHOSPHATE
LEFT_COMPARTMENT CYTOSOL
RIGHT_COMPARTMENT CYTOSOL
ENZYME_LEVEL 0.0
PATHWAY CALVIN CYCLE, PENTOSE PHOSPHATE PATHWAY, RUBISCO SHUNT
k_FORWARD 0.0
k_REVERSE 0.0
//
REACTION         TALA
LEFT D-GLYCERALDEHYDE-3-PHOSPHATE + SEDOHEPTULOSE_7-PHOSPHATE
RIGHT D-FRUCTOSE_6-PHOSPHATE + D-ERYTHROSE-4-PHOSPHATE
LEFT_COMPARTMENT CYTOSOL
RIGHT_COMPARTMENT CYTOSOL
ENZYME_LEVEL 0.0
PATHWAY RUBISCO SHUNT, PENTOSE PHOSPHATE PATHWAY
k_FORWARD 0.0
k_REVERSE 0.0
//
REACTION         PGM
LEFT 3-PHOSPHO-D-GLYCERATE
RIGHT 2-PHOSPHO-D-GLYCERATE
LEFT_COMPARTMENT CYTOSOL
RIGHT_COMPARTMENT CYTOSOL
ENZYME_LEVEL 1.0
PATHWAY GLUCONEOGENESIS, RUBISCO SHUNT, GLYCOLYSIS I
k_FORWARD 0.0011932320055
k_REVERSE 0.0128837015343
```

```
//
REACTION         ENO
LEFT 2-PHOSPHO-D-GLYCERATE
RIGHT PHOSPHOENOLPYRUVATE + H2O
LEFT_COMPARTMENT CYTOSOL
RIGHT_COMPARTMENT CYTOSOL
ENZYME_LEVEL 1.0
PATHWAY GLUCONEOGENESIS, RUBISCO SHUNT, GLYCOLYSIS I
k_FORWARD 0.0791920214198
k_REVERSE 0.0199883948156
//
REACTION         GND
LEFT NADPH + D-RIBULOSE-5-PHOSPHATE + CO2
RIGHT NADP+ + 6-PHOSPHO-D-GLUCONATE
LEFT_COMPARTMENT CYTOSOL
RIGHT_COMPARTMENT CYTOSOL
ENZYME_LEVEL 0.0
PATHWAY PENTOSE PHOSPHATE PATHWAY
k_FORWARD 0.0
k_REVERSE 0.0
//
REACTION         PGL
LEFT 6-PHOSPHO-D-GLUCONATE
RIGHT D-Glucono-1,5-lactone_6-phosphate + H2O
LEFT_COMPARTMENT CYTOSOL
RIGHT_COMPARTMENT CYTOSOL
ENZYME_LEVEL 0.0
PATHWAY PENTOSE PHOSPHATE PATHWAY
k_FORWARD 0.0
k_REVERSE 0.0
//
REACTION         HEX1
LEFT BETA-D-GLUCOSE + ATP
RIGHT BETA-D-GLUCOSE-6-PHOSPHATE + ADP
LEFT_COMPARTMENT CYTOSOL
RIGHT_COMPARTMENT CYTOSOL
ENZYME_LEVEL 1.0
PATHWAY GLUCONEOGENESIS, (GLYCOLYSIS-YEAST), GLYCOLYSIS
NREGULATION ATP 2.80e-03 10
k_FORWARD 9.20996093059e-10
k_REVERSE 1.00184422035e-13
//
REACTION         PGI
LEFT BETA-D-GLUCOSE-6-PHOSPHATE
RIGHT D-FRUCTOSE_6-PHOSPHATE
LEFT_COMPARTMENT CYTOSOL
RIGHT_COMPARTMENT CYTOSOL
ENZYME_LEVEL 1.0
PATHWAY GLUCONEOGENESIS, (GLYCOLYSIS-YEAST), GLYCOLYSIS
k_FORWARD 9.94481490048e-08
k_REVERSE 3.98304496959e-08
//
REACTION         G6PDH2r
```

```
LEFT BETA-D-GLUCOSE-6-PHOSPHATE + NADP+
RIGHT D-Glucono-1,5-lactone_6-phosphate + NADPH
LEFT_COMPARTMENT CYTOSOL
RIGHT_COMPARTMENT CYTOSOL
ENZYME_LEVEL 0.0
PATHWAY GLUCONEOGENESIS
k_FORWARD 0.0
k_REVERSE 0.0
//
REACTION         PFK
LEFT D-FRUCTOSE_6-PHOSPHATE + ATP
RIGHT ADP + D-FRUCTOSE_1,6-BISPHOSPHATE
LEFT_COMPARTMENT CYTOSOL
RIGHT_COMPARTMENT CYTOSOL
ENZYME_LEVEL 1.0
PATHWAY GLYCOLYSIS
NREGULATION ATP 2.80e-03 10
COMMNENT From Equilibrator
k_FORWARD 1.45898263083e-09
k_REVERSE 1.68336626358e-13
//
REACTION         PYRt2m
LEFT PYRUVATE
RIGHT PYRUVATE
LEFT_COMPARTMENT CYTOSOL
RIGHT_COMPARTMENT MITOCHONDRIA
ENZYME_LEVEL 1.0
DGZERO -5.94
DGZERO-UNITS KJ/MOL
k_FORWARD 0.219582252566
k_REVERSE 0.0199938495337
//
REACTION         PDHm
LEFT COA + NAD+ + PYRUVATE
RIGHT ACETYL-COA + CO2 + NADH
LEFT_COMPARTMENT MITOCHONDRIA
RIGHT_COMPARTMENT MITOCHONDRIA
ENZYME_LEVEL 1.0
PATHWAY ACETYL COA BIOSYNTHESIS
NREGULATION ACETYL-COA 1.00e-03 20
k_FORWARD 5.97022735957e-10
k_REVERSE 2.66631445841e-14
//
REACTION         ICL
LEFT isocitrate
RIGHT glyoxylate + succinate
LEFT_COMPARTMENT GLYOXYSOME
RIGHT_COMPARTMENT GLYOXYSOME
ENZYME_LEVEL 0.0
PATHWAY glyoxylate shunt
k_FORWARD 0.0
k_REVERSE 0.0
//
```

```
REACTION         MAS
LEFT acetyl-CoA + glyoxylate + H2O
RIGHT (S)-MALATE + COA
LEFT_COMPARTMENT GLYOXYSOME
RIGHT_COMPARTMENT GLYOXYSOME
ENZYME_LEVEL 0.0
PATHWAY glyoxylate shunt
k_FORWARD 0.0
k_REVERSE 0.0
//
REACTION         PYRDC
LEFT pyruvate
RIGHT acetaldehyde + CO2
LEFT_COMPARTMENT CYTOSOL
RIGHT_COMPARTMENT CYTOSOL
ENZYME_LEVEL 0.0
PATHWAY fermentation
k_FORWARD 0.0
k_REVERSE 0.0
//
REACTION         ALDD2y
LEFT acetaldehyde + NADP+ + H2O
RIGHT acetate + NADPH
LEFT_COMPARTMENT CYTOSOL
RIGHT_COMPARTMENT CYTOSOL
ENZYME_LEVEL 0.0
PATHWAY fermentation
k_FORWARD 0.0
k_REVERSE 0.0
//
REACTION         ALCD2X_copy1
LEFT acetaldehyde + NADH
RIGHT ethanol + NAD+
LEFT_COMPARTMENT CYTOSOL
RIGHT_COMPARTMENT CYTOSOL
ENZYME_LEVEL 0.0
PATHWAY fermentation
k_FORWARD 0.0
k_REVERSE 0.0
//
REACTION         LactateDehydrogenase
LEFT pyruvate + NADH
RIGHT lactate + NAD+
LEFT_COMPARTMENT CYTOSOL
RIGHT_COMPARTMENT CYTOSOL
ENZYME_LEVEL 0.0
PATHWAY fermentation
k_FORWARD 0.0
k_REVERSE 0.0
//
```

Turning on Regulation and Setting Enzyme Levels

To use regulation in the simulation, this flag must be set in the input (*.in) file:

USE_REGULATION 1

If USE_REGULATION is set to 0, then the regulation information in the .dat file will be ignored.

Reactions can be turned on (1.0) or off (0.0) by setting the ENZYME_LEVEL feature in the .dat file for a reaction:

USE_ENZYME_LEVELS 1.0

or

USE_ENZYME_LEVELS 0.0

In addition, USE_ACTIVITY 1" must be set in the input (*.in):

In [17]:
```
cat neurospora_pentose_phos.glycolysis.tca_reg_rate.in
```

```
RXN_FILE neurospora_pentose_phos.glycolysis.tca.2_rate.dat
INIT_FILE neurospora_pentose_phos.glycolysis.tca.2_reg.rstrt
LOG_FILE neurospora_pentose_phos.glycolysis.tca.2_reg_rate.log
OUT_FILE neurospora_pentose_phos.glycolysis.tca.2_reg_rate.out
USE_DEQ 1
NO_ROUND_FROM_DEQ 1
ODE_T_FINAL 10000000
ODE_T_FINAL 10
ODE_T_FINAL 0.01
ODE_T_FINAL 1000.0
DELTA_CONCS_CHOICE 10
ODE_RXN_VIEW_FREQ 1000
ODE_RXN_VIEW_FREQ 1
DERIV_THRESH  5.0e-17
WARMUP_STEPS 00000000
RECORD_STEPS 000000
TEMP_KELVIN 298.15
PH 7.0
IONIC_STRENGTH 0.15
PRINT_OUTPUT 2
CONCS_OR_COUNTS 3
RXN_VIEW_FREQ 100
COUNT_VIEW_FREQ 100
LKLHD_VIEW_FREQ 100
USE_BULK_WATER 1
USE_REGULATION 1
USE_ENZYME_LEVELS 1
USE_PSEUDOISOMERS 1
USE_DGZERO 1
USE_ACTIVITIES 1
NUM_METABOLIC_GROUPS 1
```

Models and Simulations

Model With Regulation

Run Deterministic Simulation

In [18]:
```
#args = ("boltzmann", "neurospora_pentose_phos.glycolysis.tca_reg_rate
.in")
#Or just:
#args = "bin/bar -c somefile.xml -d text.txt -r aString -f anotherStri
ng".split()
#popen = subprocess.Popen(args, stdout=subprocess.PIPE)
#popen.wait()
#output = popen.stdout.read()
#print(output)
```

Derivatives from ODE kinetic rate law simulation

In [19]:
```
# Read boltzmann derivatives
ode_derivatives = pd.read_table('neurospora_pentose_phos.glycolysis.tc
a.2_reg_rate.ode_dconcs',header=0,index_col=0)
derivatives = np.abs(ode_derivatives.iloc[-1,:])
display(derivatives.sort_values())
plt.figure();ode_derivatives.plot(legend=False,logx=True,logy=False,fi
gsize=(10, 10))

plt.yscale('symlog')
#plt.legend(bbox_to_anchor=(1.35, 1.05),bbox_transform=plt.gcf().trans
Figure)
```

```
(S)-MALATE:CYTOSOL                              0.000000e+00
SEDOHEPTULOSE_7-PHOSPHATE:CYTOSOL               0.000000e+00
(S)-MALATE:GLYOXYSOME                           0.000000e+00
ACETYL-COA:GLYOXYSOME                           0.000000e+00
COA:GLYOXYSOME                                  0.000000e+00
GLYOXYLATE:GLYOXYSOME                           0.000000e+00
ISOCITRATE:GLYOXYSOME                           0.000000e+00
SUCCINATE:GLYOXYSOME                            0.000000e+00
(S)-MALATE:MITOCHONDRIA                         0.000000e+00
2-OXOGLUTARATE:MITOCHONDRIA                     0.000000e+00
ACETYL-COA:MITOCHONDRIA                         0.000000e+00
ADP:MITOCHONDRIA                                0.000000e+00
ATP:MITOCHONDRIA                                0.000000e+00
PYRUVATE:CYTOSOL                                0.000000e+00
CITRATE:MITOCHONDRIA                            0.000000e+00
COA:MITOCHONDRIA                                0.000000e+00
FUMARATE:MITOCHONDRIA                           0.000000e+00
ISOCITRATE:MITOCHONDRIA                         0.000000e+00
NAD+:MITOCHONDRIA                               0.000000e+00
NADH:MITOCHONDRIA                               0.000000e+00
NADP+:MITOCHONDRIA                              0.000000e+00
NADPH:MITOCHONDRIA                              0.000000e+00
ORTHOPHOSPHATE:MITOCHONDRIA                     0.000000e+00
OXALOACETATE:MITOCHONDRIA                       0.000000e+00
PYRUVATE:MITOCHONDRIA                           0.000000e+00
REDOX1:MITOCHONDRIA                             0.000000e+00
```

```
REDOX2:MITOCHONDRIA                              0.000000e+00
CO2:MITOCHONDRIA                                 0.000000e+00
PHOSPHOENOLPYRUVATE:CYTOSOL                      0.000000e+00
OXALOACETATE:CYTOSOL                             0.000000e+00
ORTHOPHOSPHATE:CYTOSOL                           0.000000e+00
3-PHOSPHO-D-GLYCERATE:CYTOSOL                    0.000000e+00
3-PHOSPHO-D-GLYCEROYL_PHOSPHATE:CYTOSOL          0.000000e+00
6-PHOSPHO-D-GLUCONATE:CYTOSOL                    0.000000e+00
ACETALDEHYDE:CYTOSOL                             0.000000e+00
ACETATE:CYTOSOL                                  0.000000e+00
ADP:CYTOSOL                                      0.000000e+00
ATP:CYTOSOL                                      0.000000e+00
BETA-D-GLUCOSE:CYTOSOL                           0.000000e+00
BETA-D-GLUCOSE-6-PHOSPHATE:CYTOSOL               0.000000e+00
CO2:CYTOSOL                                      0.000000e+00
D-ERYTHROSE-4-PHOSPHATE:CYTOSOL                  0.000000e+00
D-FRUCTOSE_1,6-BISPHOSPHATE:CYTOSOL              0.000000e+00
SUCCINATE:MITOCHONDRIA                           0.000000e+00
D-FRUCTOSE_6-PHOSPHATE:CYTOSOL                   0.000000e+00
D-GLYCERALDEHYDE-3-PHOSPHATE:CYTOSOL             0.000000e+00
D-RIBOSE-5-PHOSPHATE:CYTOSOL                     0.000000e+00
D-RIBULOSE-5-PHOSPHATE:CYTOSOL                   0.000000e+00
D-XYLULOSE:CYTOSOL                               0.000000e+00
D-XYLULOSE-5-PHOSPHATE:CYTOSOL                   0.000000e+00
ETHANOL:CYTOSOL                                  0.000000e+00
GLYCERONE_PHOSPHATE:CYTOSOL                      0.000000e+00
LACTATE:CYTOSOL                                  0.000000e+00
NAD+:CYTOSOL                                     0.000000e+00
NADH:CYTOSOL                                     0.000000e+00
NADP+:CYTOSOL                                    0.000000e+00
NADPH:CYTOSOL                                    0.000000e+00
D-GLUCONO-1,5-LACTONE_6-PHOSPHATE:CYTOSOL        0.000000e+00
SUCCINYL-COA:MITOCHONDRIA                        0.000000e+00
2-PHOSPHO-D-GLYCERATE:CYTOSOL                    5.551115e-17
Name: 999.31, dtype: float64
```

```
<matplotlib.figure.Figure at 0x1143427f0>
```

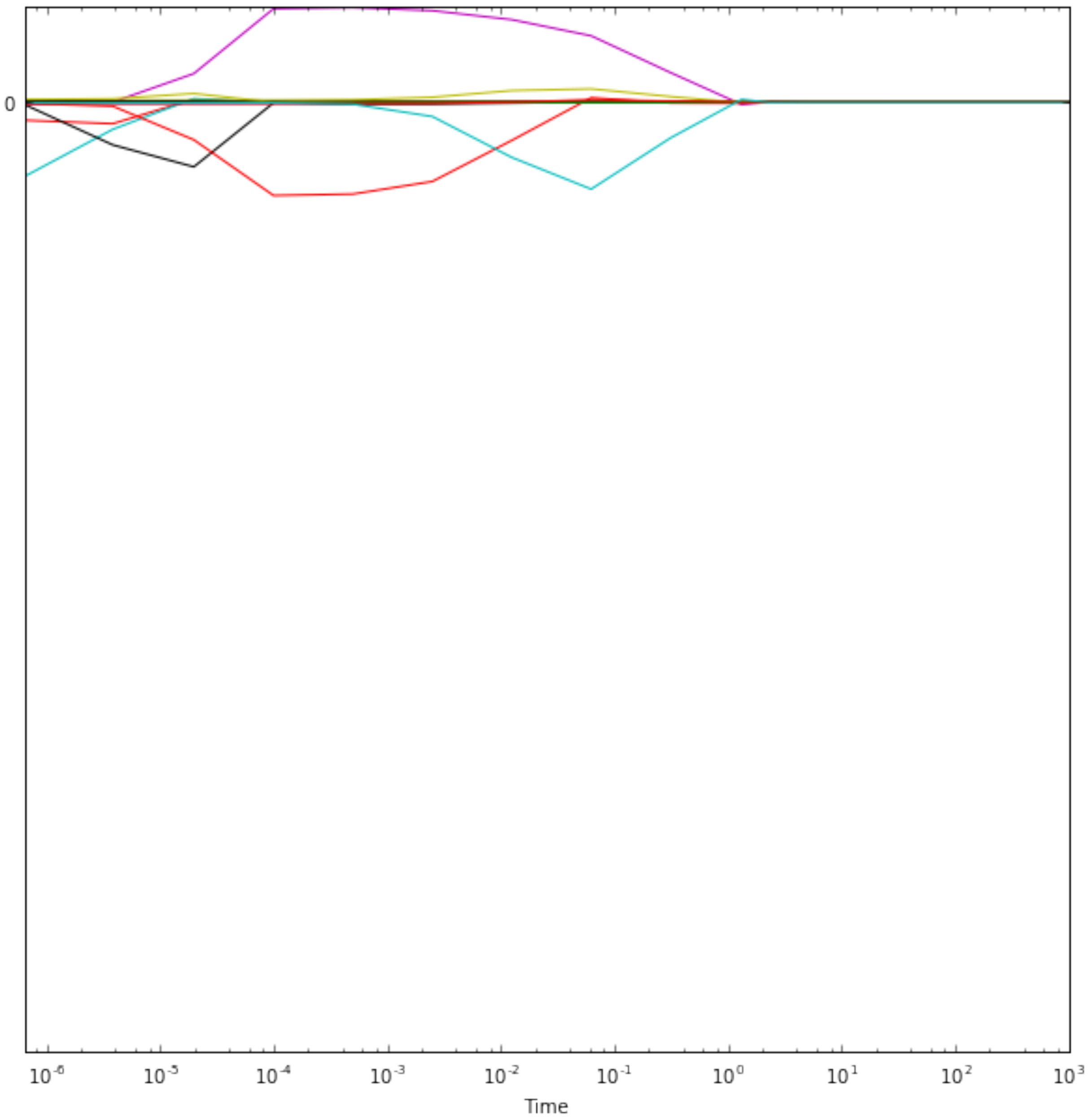

Forward and Reverse Rates from Deterministic Simulation

In [20]:

```
# Read boltzmann ODE reaction odds
ode_likelihoods_timeseries = pd.read_table('neurospora_pentose_phos.gl
ycolysis.tca.2_reg_rate.ode_lklhd',header=1, index_col = 0, quoting=2)

temp = [x for x in ode_likelihoods_timeseries.columns if 'f_' in x]
ode_fwd_likelihoods_timeseries = ode_likelihoods_timeseries[temp]
temp = [x for x in ode_likelihoods_timeseries.columns if 'r_' in x]
ode_rev_likelihoods_timeseries = ode_likelihoods_timeseries[temp]
ode_fwd_likelihoods_timeseries.columns = [x.split("f_")[-1] for x in o
de_fwd_likelihoods_timeseries.columns]
ode_rev_likelihoods_timeseries.columns = [x.split("r_")[-1] for x in o
de_rev_likelihoods_timeseries.columns]

plt.figure()
#ode_likelihoods.iloc[:,rev_column_idx].plot(legend=True,logx=True,fig
size=(12, 12))
ode_fwd_likelihoods_timeseries.plot(legend=False,logx=True,figsize=(12
, 12))
#plt.yscale('symlog')
#plt.legend(bbox_to_anchor=(1.02, 0.9),bbox_transform=plt.gcf().transF
igure)
```

Out[20]: <matplotlib.axes._subplots.AxesSubplot at 0x121a79ba8>

<matplotlib.figure.Figure at 0x121a9f1d0>

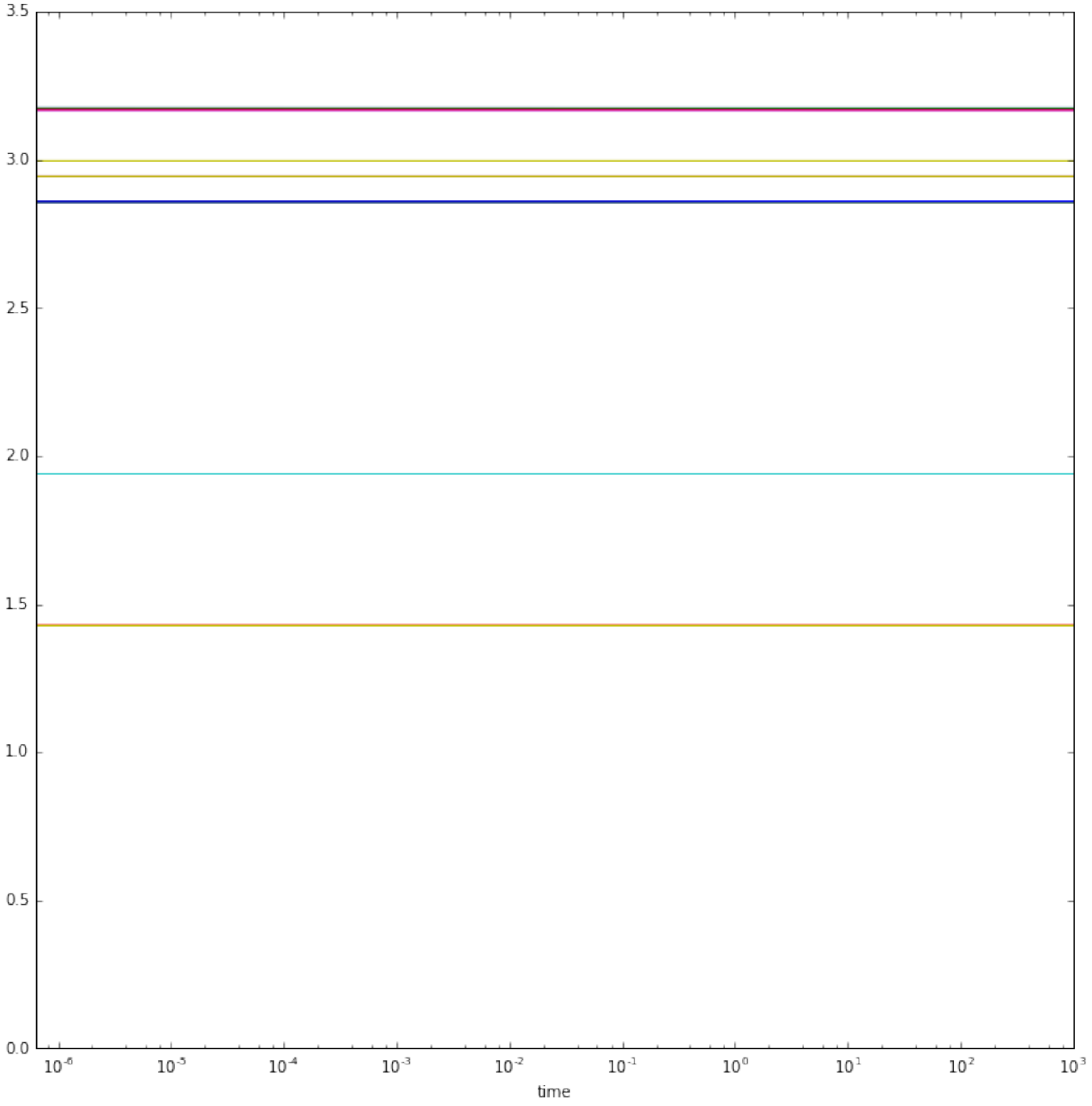

In [21]:
```
ode_likelihoods_steadystate = pd.DataFrame(data = ode_fwd_likelihoods_
timeseries.iloc[-20:].mean(),
                                          index = ode_fwd_likelihoods
_timeseries.columns,
                                          columns =['Forward'])
ode_likelihoods_steadystate['Reverse'] = ode_rev_likelihoods_timeserie
s.iloc[-20:].mean()
ode_likelihoods_steadystate['For-Rev'] = ode_likelihoods_steadystate['
Forward'] - ode_likelihoods_steadystate['Reverse']
ode_likelihoods_steadystate['Rxn Probabilities'] = ode_likelihoods_ste
adystate['For-Rev']/np.sum(abs(ode_likelihoods_steadystate['For-Rev'])
)
ode_likelihoods_steadystate
```

Out[21]:

Out[21]:

	Forward	Reverse	For-Rev	Rxn Probabilities
**ME1m**	0.000000	0.000000e+00	0.000000	0.000000
**ME2m**	0.000000	0.000000e+00	0.000000	0.000000
**CSm**	3.167759	3.099478e-01	2.857811	0.057144
**ACONTm**	3.172947	3.151333e-01	2.857814	0.057144
**ICDHxm**	2.943089	8.531388e-02	2.857775	0.057144
**AKGDm**	2.943124	8.532100e-02	2.857803	0.057144
**SUCOASm**	3.172931	3.151645e-01	2.857766	0.057143
**SUCD1m**	3.172834	3.151739e-01	2.857660	0.057141
**FUMm**	3.172531	3.152049e-01	2.857326	0.057135
**MDHm**	3.167089	3.108204e-01	2.856269	0.057113
**GAPD**	2.857815	7.730477e-07	2.857814	0.057144
**PGK**	3.165479	3.076658e-01	2.857813	0.057144
**TPI**	1.428909	1.511411e-06	1.428907	0.028572
**MDH**	0.000000	0.000000e+00	0.000000	0.000000
**PEP_Carboxylase**	0.000000	0.000000e+00	0.000000	0.000000
**PPCK**	0.000000	0.000000e+00	0.000000	0.000000
**FBA**	1.428908	1.511408e-06	1.428906	0.028572
**FBP**	0.000000	0.000000e+00	0.000000	0.000000
**TKT2**	0.000000	0.000000e+00	0.000000	0.000000
**RPE**	0.000000	0.000000e+00	0.000000	0.000000
**Xylulokinase**	0.000000	0.000000e+00	0.000000	0.000000
**PYK_org**	0.000000	0.000000e+00	0.000000	0.000000
**PYK**	2.857812	3.583479e-05	2.857776	0.057144
**RPI**	0.000000	0.000000e+00	0.000000	0.000000
**TKT1**	0.000000	0.000000e+00	0.000000	0.000000
**TALA**	0.000000	0.000000e+00	0.000000	0.000000
**PGM**	2.995261	1.374769e-01	2.857784	0.057144
**ENO**	2.857838	7.274040e-05	2.857765	0.057143
**GND**	0.000000	0.000000e+00	0.000000	0.000000
**PGL**	0.000000	0.000000e+00	0.000000	0.000000

**HEX1**	1.428907	1.389075e-11	1.428907	0.028572
**PGI**	1.943454	5.145463e-01	1.428908	0.028572
**G6PDH2r**	0.000000	0.000000e+00	0.000000	0.000000
**PFK**	1.428907	1.389073e-11	1.428907	0.028572
**PYRt2m**	2.857773	8.044882e-06	2.857765	0.057143
**PDHm**	2.859040	3.883754e-05	2.859001	0.057168
**ICL**	0.000000	0.000000e+00	0.000000	0.000000
**MAS**	0.000000	0.000000e+00	0.000000	0.000000
**PYRDC**	0.000000	0.000000e+00	0.000000	0.000000
**ALDD2y**	0.000000	0.000000e+00	0.000000	0.000000
**ALCD2X_copy1**	0.000000	0.000000e+00	0.000000	0.000000
**LactateDehydrogenase**	0.000000	0.000000e+00	0.000000	0.000000

In [22]:

```
from escher import Builder

reaction_data = ode_likelihoods_steadystate['For-Rev'].to_dict()
b = Builder(map_name="iMM904.compact_Glycolysis_TCA_PPP.json",reaction
_data=reaction_data)
b.display_in_notebook(menu='zoom')
#b.display_in_browser(menu='all')
```

Out[22]:

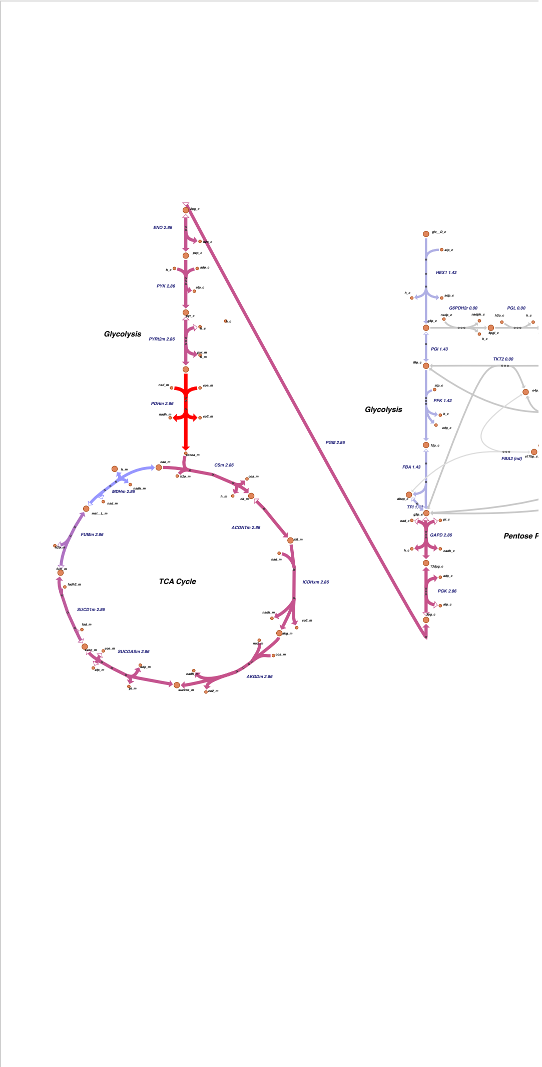

Analyze Metabolite Levels from Deterministic Simulation

In [23]:

```
# Read boltzmann stochastic reaction likelihoods
ode_metabolites = pd.read_table('neurospora_pentose_phos.glycolysis.tca.2_reg_rate.ode_concs',header=0,index_col=0)

plt.figure();ode_metabolites.plot(legend=False,logx=True,logy=True,figsize=(10, 10))
plt.legend(bbox_to_anchor=(1.35, 0.95),bbox_transform=plt.gcf().transFigure)
```

Out[23]: <matplotlib.legend.Legend at 0x121ebd978>

<matplotlib.figure.Figure at 0x115b395c0>

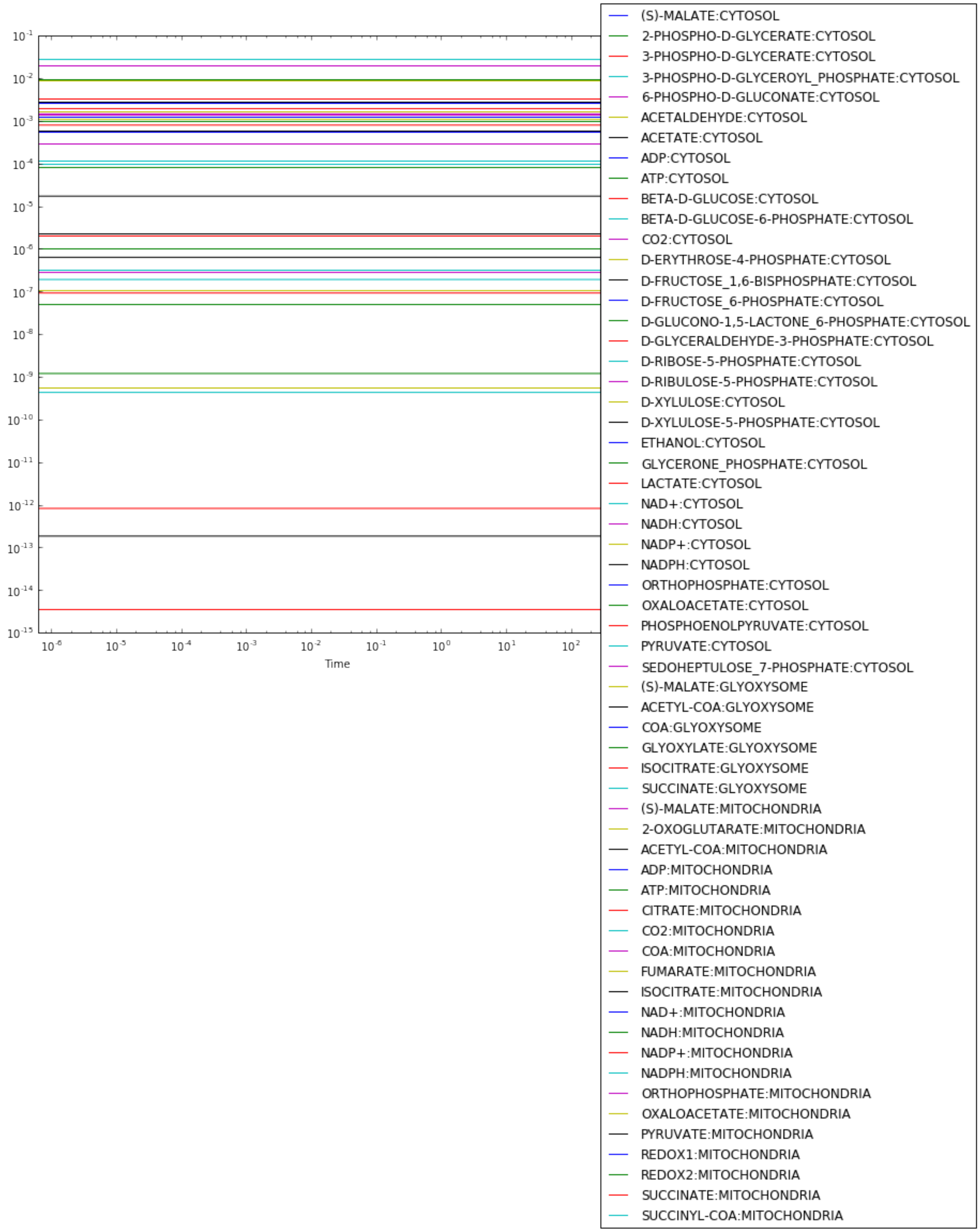

In [24]:

```
#ode_metabolites_steadystate = pd.DataFrame()
ode_metabolites_steadystate = \
  pd.DataFrame(data = ode_metabolites.iloc[-20:,:].mean(axis=0),column
s=['Mean'])
ode_metabolites_steadystate['CV'] = \
  pd.DataFrame(data = ode_metabolites.iloc[-20:,:].var(axis=0)/ode_met
abolites.iloc[-20:,:].mean(axis=0),columns=['ODE'])
ode_metabolites_steadystate['Ratio'] = \
  pd.DataFrame(data =(ode_metabolites.iloc[-1]/ode_metabolites.iloc[0]
))
ode_metabolites_steadystate['Counts'] = ode_metabolites_steadystate['M
ean']*Concentration2Count
display(ode_metabolites_steadystate)
```

	Mean	CV	Ratio	C
**(S)-MALATE:CYTOSOL**	1.700000e-03	2.911441e-35	1.000000	1.02376
**2-PHOSPHO-D-GLYCERATE:CYTOSOL**	1.198391e-09	1.502514e-40	0.999983	7.21687
**3-PHOSPHO-D-GLYCERATE:CYTOSOL**	9.246002e-08	0.000000e+00	0.999983	5.56807
**3-PHOSPHO-D-GLYCEROYL_PHOSPHATE:CYTOSOL**	1.915081e-07	0.000000e+00	0.999986	1.15328
**6-PHOSPHO-D-GLUCONATE:CYTOSOL**	0.000000e+00	NaN	NaN	0.00000
**ACETALDEHYDE:CYTOSOL**	0.000000e+00	NaN	NaN	0.00000
**ACETATE:CYTOSOL**	0.000000e+00	NaN	NaN	0.00000
**ADP:CYTOSOL**	5.600000e-04	2.209576e-35	1.000000	3.37239
**ATP:CYTOSOL**	9.600000e-03	0.000000e+00	1.000000	5.78125
**BETA-D-GLUCOSE:CYTOSOL**	2.000000e-03	3.959560e-34	1.000000	1.20442
**BETA-D-GLUCOSE-6-PHOSPHATE:CYTOSOL**	9.827226e-04	5.036467e-35	1.000000	5.91809
**CO2:CYTOSOL**	1.000000e-04	1.933379e-36	1.000000	6.02214
**D-ERYTHROSE-4-PHOSPHATE:CYTOSOL**	5.149502e-08	8.951418e-40	1.000000	3.10110
**D-FRUCTOSE_1,6-BISPHOSPHATE:CYTOSOL**	5.848601e-04	8.462622e-35	1.000000	3.52211
**D-FRUCTOSE_6-PHOSPHATE:CYTOSOL**	1.262518e-03	3.920300e-35	1.000000	7.60306
**D-GLUCONO-1,5-LACTONE_6-**				

**PHOSPHATE:CYTOSOL**	0.000000e+00	NaN	NaN	0.00000[illegible]
**D-GLYCERALDEHYDE-3-PHOSPHATE:CYTOSOL**	3.700402e-15	1.770225e-46	0.999995	2.22843[illegible]
**D-RIBOSE-5-PHOSPHATE:CYTOSOL**	3.189369e-07	0.000000e+00	1.000000	1.92068[illegible]
**D-RIBULOSE-5-PHOSPHATE:CYTOSOL**	2.890365e-07	0.000000e+00	1.000000	1.74061[illegible]
**D-XYLULOSE:CYTOSOL**	8.800000e-03	0.000000e+00	1.000000	5.29948[illegible]
**D-XYLULOSE-5-PHOSPHATE:CYTOSOL**	2.320598e-06	8.136115e-38	1.000000	1.39749[illegible]
**ETHANOL:CYTOSOL**	0.000000e+00	NaN	NaN	0.00000[illegible]
**GLYCERONE_PHOSPHATE:CYTOSOL**	5.215589e-08	8.837994e-40	1.000001	3.14090[illegible]
**LACTATE:CYTOSOL**	0.000000e+00	NaN	NaN	0.00000[illegible]
**NAD+:CYTOSOL**	2.600000e-03	3.045815e-34	1.000000	1.56575[illegible]
**NADH:CYTOSOL**	8.300000e-05	9.317489e-36	1.000000	4.99837[illegible]
**NADP+:CYTOSOL**	2.100000e-06	8.990787e-38	1.000000	1.26465[illegible]
**NADPH:CYTOSOL**	1.200000e-04	1.611149e-36	1.000000	7.22656[illegible]
**ORTHOPHOSPHATE:CYTOSOL**	2.000000e-02	6.335296e-34	1.000000	1.20442[illegible]
**OXALOACETATE:CYTOSOL**	1.000000e-03	1.979780e-34	1.000000	6.02214[illegible]
**PHOSPHOENOLPYRUVATE:CYTOSOL**	8.239156e-13	1.302610e-44	0.999983	4.96173[illegible]
**PYRUVATE:CYTOSOL**	4.321398e-10	6.510478e-42	0.999983	2.60240[illegible]
**SEDOHEPTULOSE_7-PHOSPHATE:CYTOSOL**	3.035266e-04	1.019155e-35	1.000000	1.82788[illegible]
**(S)-MALATE:GLYOXYSOME**	1.700000e-03	2.911441e-35	1.000000	1.02376[illegible]
**ACETYL-COA:GLYOXYSOME**	2.863050e-03	6.914933e-35	1.000000	1.72416[illegible]
**COA:GLYOXYSOME**	1.400000e-03	3.181789e-34	1.000000	8.43099[illegible]
**GLYOXYLATE:GLYOXYSOME**	1.000000e-06	4.720163e-38	1.000000	6.02214[illegible]
**ISOCITRATE:GLYOXYSOME**	1.000000e-03	1.979780e-34	1.000000	6.02214[illegible]
**SUCCINATE:GLYOXYSOME**	5.700000e-04	2.170811e-35	1.000000	3.43262[illegible]
**(S)-MALATE:MITOCHONDRIA**	1.459541e-03	3.051990e-34	1.000187	8.78956[illegible]
**2-OXOGLUTARATE:MITOCHONDRIA**	5.568240e-10	2.021060e-41	0.999985	3.35327[illegible]
**ACETYL-COA:MITOCHONDRIA**	6.333631e-07	0.000000e+00	0.999343	3.81420[illegible]
**ADP:MITOCHONDRIA**	5.600000e-04	2.209576e-35	1.000000	3.37239[illegible]

**ATP:MITOCHONDRIA**	9.600000e-03	0.000000e+00	1.000000	5.78125
**CITRATE:MITOCHONDRIA**	7.979793e-04	6.202479e-35	0.999983	4.80554
**CO2:MITOCHONDRIA**	1.000000e-04	1.933379e-36	1.000000	6.02214
**COA:MITOCHONDRIA**	1.400000e-03	3.181789e-34	1.000000	8.43099
**FUMARATE:MITOCHONDRIA**	1.078705e-03	4.588326e-35	1.000047	6.49611
**ISOCITRATE:MITOCHONDRIA**	1.720674e-05	0.000000e+00	0.999983	1.03621
**NAD+:MITOCHONDRIA**	2.600000e-03	3.045815e-34	1.000000	1.56575
**NADH:MITOCHONDRIA**	8.300000e-05	9.317489e-36	1.000000	4.99837
**NADP+:MITOCHONDRIA**	2.100000e-06	8.990787e-38	1.000000	1.26465
**NADPH:MITOCHONDRIA**	1.200000e-04	1.611149e-36	1.000000	7.22656
**ORTHOPHOSPHATE:MITOCHONDRIA**	2.000000e-02	6.335296e-34	1.000000	1.20442
**OXALOACETATE:MITOCHONDRIA**	1.049036e-07	0.000000e+00	1.000641	6.31744
**PYRUVATE:MITOCHONDRIA**	1.848941e-13	3.627890e-45	0.999759	1.11345
**REDOX1:MITOCHONDRIA**	1.000000e-03	1.979780e-34	1.000000	6.02214
**REDOX2:MITOCHONDRIA**	1.000000e-03	1.979780e-34	1.000000	6.02214
**SUCCINATE:MITOCHONDRIA**	3.422563e-03	5.206046e-34	1.000003	2.06111
**SUCCINYL-COA:MITOCHONDRIA**	2.915206e-02	4.346380e-34	0.999989	1.75557

Reaction Rates from Kinetic Rate Law

Check to see that the rates calculated from the usual mass action rate law using rate constants is the same as the rates calculated above from thermodynamic odds.

Since the rate constants were determined from the net reaction probabilities determined from the maximum entropy distribution modified by including regulation, the flux values from the kinetic rate law calculated below should be compared to those net reaction probabilities.

In [25]:
```
# Read in rates constants:
reaction_df = pd.DataFrame()
filename = 'neurospora_pentose_phos.glycolysis.tca.2_rate.dat'
with open(filename,'r') as f:
  for line in f:
     #print(line)
     if re.match('^REACTION', line):
        temp = re.split('\s',line,1)
        rxn_name = temp[1].strip()
```

```
        if not rxn_name:
          print("Error: Reaction name not found:\n", line)
     elif re.match('^COMMENT',line):
        continue
     elif re.match(r'//',line):
        continue
     elif re.match('^#',line):
        continue
     else:
        #print(line)
        rxn_pair = re.split('\s',line,1)
        reaction_df.loc[rxn_pair[0],rxn_name] = rxn_pair[1].strip()

     # end if
  # end for loop
# end with loop
reaction_df = reaction_df.T
reaction_df['k_FORWARD'] = reaction_df['k_FORWARD'].astype(np.float64)
reaction_df['k_REVERSE'] = reaction_df['k_REVERSE'].astype(np.float64)
del reaction_df['DGZERO-UNITS']
del reaction_df['DGZERO']
del reaction_df['COMMNENT']
del reaction_df['PATHWAY']

# Read in stoichiometric matrix and get reactant and product matrices
S = pd.read_table('neurospora_pentose_phos.glycolysis.tca.2_reg_rate.m
at',header=0, index_col = 0, quoting=2)
P = (S>0)
R = S < 0
del P['forward reaction']
del R['forward reaction']

P = P.astype(np.float64)
R = R.astype(np.float64)

# Calculate rates:
# v_{f} = exp(log(k_f) + R log C)
# v_{r} = exp(log(k_r) + P log C)
# v_{net} = v_{f} - v_{r}
reactant_concentrations = R.multiply(ode_metabolites_steadystate['Coun
ts'],axis=1)
reactant_concentrations[reactant_concentrations == 0] = 1
reactant_concentrations_rxns = (reactant_concentrations.T).product()

product_concentrations = P.multiply(ode_metabolites_steadystate['Count
s'],axis=1)
product_concentrations[product_concentrations == 0] = 1
product_concentrations_rxns = (product_concentrations.T).product()

reaction_df['Fwd Rate'] = reaction_df['k_FORWARD'].multiply(reactant_c
oncentrations_rxns)
reaction_df['Rev Rate'] = reaction_df['k_REVERSE'].multiply(product_co
ncentrations_rxns)
```

```
reaction_df['Net Rate'] = reaction_df['Fwd Rate']-reaction_df['Rev Rat
e']
display(reaction_df)
```

	LEFT	RIGHT	LEFT_(
**ME1m**	(S)-MALATE + NAD+	pyruvate + NADH + CO2	MITOCI
**ME2m**	(S)-MALATE + NADP+	PYRUVATE + NADPH + CO2	MITOCI
**CSm**	OXALOACETATE + ACETYL-COA + H2O	CITRATE + COA	MITOCI
**ACONTm**	CITRATE	ISOCITRATE	MITOCI
**ICDHxm**	ISOCITRATE + NAD+	2-OXOGLUTARATE + NADH + CO2	MITOCI
**AKGDm**	2-OXOGLUTARATE + COA + NAD+	SUCCINYL-COA + CO2 + NADH	MITOCI
**SUCOASm**	SUCCINYL-COA + ADP + Orthophosphate	SUCCINATE + ATP + COA	MITOCI
**SUCD1m**	SUCCINATE + redox1	FUMARATE + redox2	MITOCI
**FUMm**	FUMARATE + H2O	(S)-MALATE	MITOCI
**MDHm**	(S)-MALATE + NAD+	OXALOACETATE + NADH	MITOCI
**GAPD**	D-GLYCERALDEHYDE-3-PHOSPHATE + ORTHOPHOSPHATE ...	3-Phospho-D-glyceroyl_phosphate + NADH	CYTOS
**PGK**	3-Phospho-D-glyceroyl_phosphate + ADP	3-PHOSPHO-D-GLYCERATE + ATP	CYTOS
**TPI**	Glycerone_phosphate	D-GLYCERALDEHYDE-3-PHOSPHATE	CYTOS
**MDH**	(S)-MALATE + NAD+	OXALOACETATE + NADH	CYTOS
**PEP_Carboxylase**	oxaloacetate + orthophosphate	phosphoenolpyruvate + CO2 + H2O	CYTOS
**PPCK**	oxaloacetate + ATP	phosphoenolpyruvate + ADP + CO2	CYTOS
**FBA**	D-FRUCTOSE_1,6-BISPHOSPHATE	Glycerone_phosphate + D-GLYCERALDEHYDE-3-PHOSP...	CYTOS
**FBP**	D-FRUCTOSE_6-PHOSPHATE +	H2O + D-FRUCTOSE_1,6-BISPHOSPHATE	CYTOS

	Orthophosphate		
**TKT2**	D-FRUCTOSE_6-PHOSPHATE + D-GLYCERALDEHYDE-3-PH...	D-ERYTHROSE-4-PHOSPHATE + D-XYLULOSE-5-PHOSPHATE	CYTOS
**RPE**	D-XYLULOSE-5-PHOSPHATE	D-RIBULOSE-5-PHOSPHATE	CYTOS
**Xylulokinase**	D-XYLULOSE + ATP	D-XYLULOSE-5-PHOSPHATE + ADP	CYTOS
**PYK_org**	ADP + PHOSPHOENOLPYRUVATE	PYRUVATE + ATP	CYTOS
**PYK**	ADP + PHOSPHOENOLPYRUVATE	PYRUVATE + ATP	CYTOS
**RPI**	D-RIBOSE-5-PHOSPHATE	D-RIBULOSE-5-PHOSPHATE	CYTOS
**TKT1**	SEDOHEPTULOSE_7-PHOSPHATE + D-GLYCERALDEHYDE-3...	D-RIBOSE-5-PHOSPHATE + D-XYLULOSE-5-PHOSPHATE	CYTOS
**TALA**	D-GLYCERALDEHYDE-3-PHOSPHATE + SEDOHEPTULOSE_7...	D-FRUCTOSE_6-PHOSPHATE + D-ERYTHROSE-4-PHOSPHATE	CYTOS
**PGM**	3-PHOSPHO-D-GLYCERATE	2-PHOSPHO-D-GLYCERATE	CYTOS
**ENO**	2-PHOSPHO-D-GLYCERATE	PHOSPHOENOLPYRUVATE + H2O	CYTOS
**GND**	NADPH + D-RIBULOSE-5-PHOSPHATE + CO2	NADP+ + 6-PHOSPHO-D-GLUCONATE	CYTOS
**PGL**	6-PHOSPHO-D-GLUCONATE	D-Glucono-1,5-lactone_6-phosphate + H2O	CYTOS
**HEX1**	BETA-D-GLUCOSE + ATP	BETA-D-GLUCOSE-6-PHOSPHATE + ADP	CYTOS
**PGI**	BETA-D-GLUCOSE-6-PHOSPHATE	D-FRUCTOSE_6-PHOSPHATE	CYTOS
**G6PDH2r**	BETA-D-GLUCOSE-6-PHOSPHATE + NADP+	D-Glucono-1,5-lactone_6-phosphate + NADPH	CYTOS
**PFK**	D-FRUCTOSE_6-PHOSPHATE + ATP	ADP + D-FRUCTOSE_1,6-BISPHOSPHATE	CYTOS

**PYRt2m**	PYRUVATE	PYRUVATE	CYTOS
**PDHm**	COA + NAD+ + PYRUVATE	ACETYL-COA + CO2 + NADH	MITOCI
**ICL**	isocitrate	glyoxylate + succinate	GLYOX
**MAS**	acetyl-CoA + glyoxylate + H2O	(S)-MALATE + COA	GLYOX
**PYRDC**	pyruvate	acetaldehyde + CO2	CYTOS
**ALDD2y**	acetaldehyde + NADP+ + H2O	acetate + NADPH	CYTOS
**ALCD2X_copy1**	acetaldehyde + NADH	ethanol + NAD+	CYTOS
**LactateDehydrogenase**	pyruvate + NADH	lactate + NAD+	CYTOS

42 rows × 11 columns

Calculate Power

Power Characteristics

For complex systems such as biological systems, the power is the change in free energy with respect to time,

$$\begin{aligned} P &= -\frac{dG}{dt} \\ &= -\frac{dG}{d\xi}\frac{d\xi}{dt} \\ &= A\frac{d\xi}{dt} \end{aligned}$$

where A is the reaction affinity. The rate $d\xi/dt = \dot{\xi}$ is the change in the extent of the reaction ξ with time. The power generation of a reaction can then be expressed in terms of the reaction affinity A and the rate of the reaction $\dot{\xi}$. If the system is at steady state, $P_{avg} = A \cdot \dot{\xi}$ as well. This relationship is useful for comparing different chemical processes.

In [26]:
```
S = pd.read_table('neurospora_pentose_phos.glycolysis.tca.2_reg_rate.amat',header=0, index_col = 0, quoting=2)
R = (S<0)
P = S>0
del R['forward reaction']
del P['forward reaction']
del S['forward reaction']
#S['H2O:CYTOSOL'] =0
#S['H2O:GLYOXYSOME'] =0
```

```
#S['H2O:MITOCHONDRIA'] =0

volume_coeff = S.sum(axis=1)

R = R.astype(np.float64)
P = P.astype(np.float64)

steadyState_counts = ode_metabolites_steadystate['Mean']*Concentration
2Count

reactant_counts = R.multiply(steadyState_counts,axis=1)
reactant_concs =  R.multiply(ode_metabolites_steadystate['Mean'])
product_counts = P.multiply(steadyState_counts,axis=1)
product_concs = P.multiply(ode_metabolites_steadystate['Mean'])

product_counts_incremented = product_counts + P.multiply(1)
product_concs_incremented = product_concs + P.multiply(concentration_i
ncrement)
product_counts = product_counts #+ P.multiply(1)
product_concs = product_concs #+ P.multiply(concentration_increment)

# Take the product of counts of all reactants for a reaction
reactant_counts_rxns = (reactant_counts[reactant_counts != 0].T).produ
ct()
reactant_concs_rxns = (reactant_concs[reactant_concs != 0].T).product(
)

# Take the product of counts of all products for a reaction
product_counts_rxns = (product_counts[product_counts != 0].T).product(
)
product_concs_rxns = (product_concs[product_concs != 0].T).product()
product_counts_incremented_rxns = (product_counts_incremented[product_
counts_incremented != 0].T).product()
product_concs_incremented_rxns = (product_concs_incremented[product_co
ncs_incremented != 0].T).product()
#denominator = reactant_counts_rxns.multiply(1-ode_likelihoods_steadys
tate['Reverse'])

K_eq = pd.read_table('neurospora_pentose_phos.glycolysis.tca.2_reg_rat
e.dg0ke',header=0, index_col = 0, quoting=2)

result = pd.DataFrame()
result['Boltzmann Ke'] = K_eq['Ke']
# Notice that Computed L_fwd uses incremented product counts. That is
so we can compare to Boltzmann values
# when calculating the activities. That is, these need to use the same
formula.
result['Computed L_fwd'] = result['Boltzmann Ke'].multiply(reactant_co
unts_rxns.divide(product_counts_incremented_rxns))

idx = np.abs(ode_likelihoods_steadystate['For-Rev']) > 0
DeltaG = "$\Delta G$"
ode_likelihoods_steadystate[DeltaG] = np.nan
```

```
ode_likelihoods_steadystate['Power'] = np.nan
ode_likelihoods_steadystate[DeltaG][idx] = -RT*np.log(result['Computed L_fwd'][idx])
ode_likelihoods_steadystate['Power'][idx] = ode_likelihoods_steadystate['For-Rev'][idx]*np.log(result['Computed L_fwd'][idx])
display(ode_likelihoods_steadystate.loc[idx,:])
```

	Forward	Reverse	For-Rev	Rxn Probabilities	ΔG	Power
**CSm**	3.167759	3.099478e-01	2.857811	0.057144	-2.858297	3.295126
**ACONTm**	3.172947	3.151333e-01	2.857814	0.057144	-2.862354	3.299805
**ICDHxm**	2.943089	8.531388e-02	2.857775	0.057144	-2.675933	3.084853
**AKGDm**	2.943124	8.532100e-02	2.857803	0.057144	-2.675962	3.084917
**SUCOASm**	3.172931	3.151645e-01	2.857766	0.057143	-2.862340	3.299736
**SUCD1m**	3.172834	3.151739e-01	2.857660	0.057141	-2.862265	3.299526
**FUMm**	3.172531	3.152049e-01	2.857326	0.057135	-2.862029	3.298868
**MDHm**	3.167089	3.108204e-01	2.856269	0.057113	-2.857773	3.292744
**GAPD**	2.857815	7.730477e-07	2.857814	0.057144	-2.603046	3.000868
**PGK**	3.165479	3.076658e-01	2.857813	0.057144	-2.856512	3.293071
**TPI**	1.428909	1.511411e-06	1.428907	0.028572	-0.884768	0.509993
**FBA**	1.428908	1.511408e-06	1.428906	0.028572	-0.884766	0.509992
**PYK**	2.857812	3.583479e-05	2.857776	0.057144	-2.603043	3.000825
**PGM**	2.995261	1.374769e-01	2.857784	0.057144	-2.719493	3.135079
**ENO**	2.857838	7.274040e-05	2.857765	0.057143	-2.603066	3.000840

**HEX1**	1.428907	1.389075e-11	1.428907	0.028572	-31.429076	18.116184
**PGI**	1.943454	5.145463e-01	1.428908	0.028572	-1.647184	0.949462
**PFK**	1.428907	1.389073e-11	1.428907	0.028572	-31.429076	18.116184
**PYRt2m**	2.857773	8.044882e-06	2.857765	0.057143	-2.603009	3.000774
**PDHm**	2.859040	3.883754e-05	2.859001	0.057168	-2.604108	3.003339

In [27]:
```
idx = ode_likelihoods_steadystate.isnull()['Power']
ode_likelihoods_steadystate.loc[idx,'Power'] = 0.0
reaction_data = ode_likelihoods_steadystate['Power'].to_dict()
b = Builder(map_name="iMM904.compact_Glycolysis_TCA_PPP.json",reaction_data=reaction_data)
b.display_in_notebook(menu='zoom')
```

Out[27]:

+

−

↔

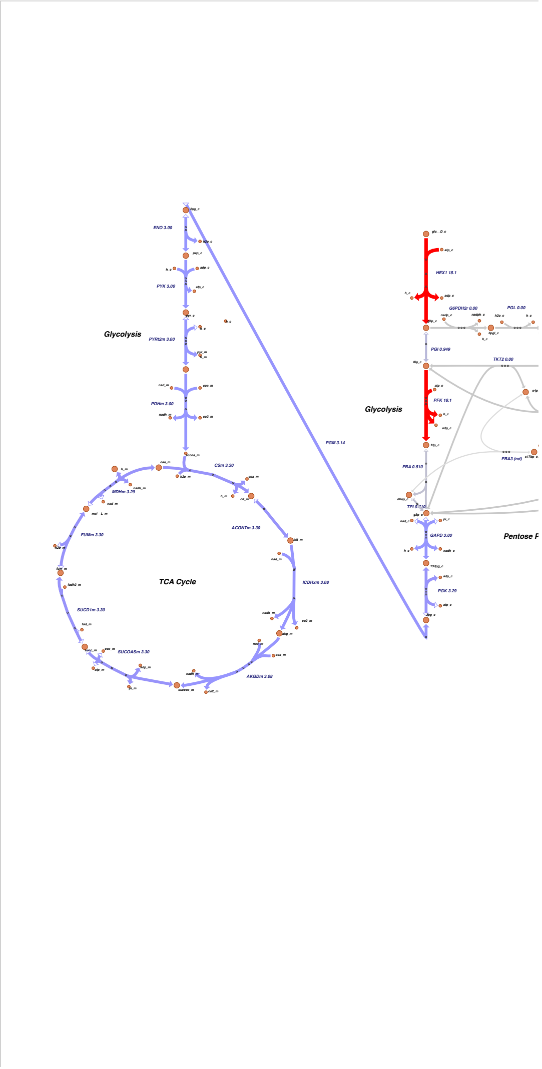

Since the free energy change is negative, each reaction will have a negative resistance. Each reaction is actually a small battery, in a sense.

A regulated reaction that has a large favorable free energy change relative to other reactions at steady state, will act as a potentiometer. That is, one could increase the flux by changing the extent of the regulation.

Calculate Resistance and Conductance

G = J/V or flux/ΔG.

In [28]:

```
idx = np.abs(ode_likelihoods_steadystate['For-Rev']) > 0
ode_likelihoods_steadystate['Resistance'] = np.nan
ode_likelihoods_steadystate['Conductance'] = np.nan
ode_likelihoods_steadystate['Conductance'][idx] = ode_likelihoods_stea
dystate['For-Rev'][idx]/((-1)*np.log(result['Computed L_fwd'][idx]))
ode_likelihoods_steadystate['Resistance'][idx] = ((1)*np.log(result['C
omputed L_fwd'][idx]))/ode_likelihoods_steadystate['For-Rev'][idx]

idx = ode_likelihoods_steadystate.notna()['Resistance']
#ode_likelihoods_steadystate.loc[idx,'Resistance'] = np.inf
reaction_data = ode_likelihoods_steadystate.loc[idx,'Resistance'].to_d
ict()

b = Builder(map_name="iMM904.compact_Glycolysis_TCA_PPP.json",reaction
_data=reaction_data)
b.display_in_notebook(menu='zoom')
```

Out[28]:

+

−

↔

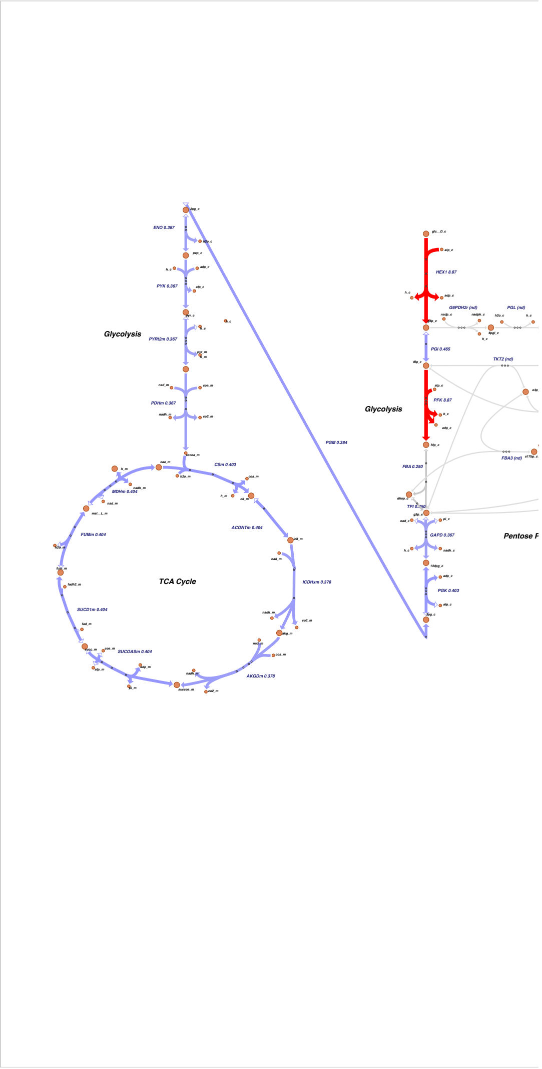

The next snippet of code generates the data needed for construction of Figure 4 in the manuscript, which is made using Pathway Tools from SRI.

In [29]:

```
biocyc_frames = pd.read_table('power_energy_characteristics.tab',heade
r=0, index_col = 0, quoting=2)
biocyc_frames.set_index(['Reaction'],append=True,inplace=True)
biocyc_frames = biocyc_frames.swaplevel(axis=0)
display(biocyc_frames)

biocyc_frames = biocyc_frames.swaplevel(axis=0)
biocyc_frames.to_csv('power_energy_characteristics2.txt', sep='\t', co
lumns = ['Energy','Power','Resistance','Flux'])
```

		Energy	Power	Resistance	Flux
Reaction	FrameId				
**CSm**	**CITSYN-RXN**	-1.153025	3.295129	0.403464	2.857812
**ACONTm**	**ACONITATEDEHYDR-RXN**	-1.154661	3.299805	0.404036	2.857814
	**ACONITATEHYDR-RXN**	-1.154661	3.299805	0.404036	2.857814
**ICDHxm**	**ISOCITRATE-DEHYDROGENASE-NAD+-RXN**	-1.079460	3.084853	0.377727	2.857775
**AKGDam**	**2OXOGLUTARATEDEH-RXN**	-1.079472	3.084917	0.377728	2.857803
**SUCOASm**	**SUCCCOASYN-RXN**	-1.154656	3.299736	0.404041	2.857766
**SUCD1m**	**SUCCINATE-DEHYDROGENASE-UBIQUINONE-RXN**	-1.154625	3.299526	0.404046	2.857660
**FUMm**	**FUMHYDR-RXN**	-1.154530	3.298868	0.404060	2.857326
**MDHm**	**MALATE-DEH-RXN**	-1.152814	3.292751	0.403608	2.856273
**GAPD**	**GAPOXNPHOSPHN-RXN**	-1.050057	3.000868	0.367434	2.857814
**PGK**	**PHOSGLYPHOS-RXN**	-1.152304	3.293071	0.403212	2.857813
**TPI**	**TRIOSPISOMERIZATION-RXN**	-0.356911	0.509993	0.249779	1.428907
**FBA**	**F16ALDOLASE-RXN**	-0.356911	0.509992	0.249779	1.428906
**PYK**	**PEPDEPHOS-RXN**	-1.050056	3.000825	0.367438	2.857776
**PGM**	**3PGAREARR-RXN**	-1.097031	3.135079	0.383875	2.857784
**ENO**	**2PGADEHYDRAT-RXN**	-1.050065	3.000840	0.367443	2.857765
**HEX1**	**GLUCOKIN-RXN**	-0.356910	18.116184	8.872761	1.428907
**PGI**	**PGLUCISOM-RXN**	-0.664467	0.949462	0.465017	1.428908
**PFK**	**6PFRUCTPHOS-RXN**	-0.356910	1.116184	8.872761	1.428907
**PYRt2m**	**TRANS-RXN2T-215**	-1.050043	3.000774	0.367435	2.857765
**PDHm**	**PYRUVDEHY-RXN**	-1.050485	3.003334	0.367431	2.858998
